# V Brazilian Consensus on Rhinitis – 2024

**DOI:** 10.1016/j.bjorl.2024.101500

**Published:** 2024-09-07

**Authors:** Dirceu Solé, Fábio Chigres Kuschnir, Antônio Carlos Pastorino, Clóvis F. Constantino, Clóvis Galvão, Débora Carla Chong e Silva, Eduardo Baptistella, Ekaterini Simões Goudouris, Eulália Sakano, Fábio Ejzenbaum, Fausto Yoshio Matsumoto, Flavio Massao Mizoguchi, Fernando Monteiro Aarestrup, Gustavo F. Wandalsen, Herberto José Chong Neto, João Vianney Brito de Oliveira, José Faibes Lubianca Neto, Maria Cândida V. Rizzo, Maria Letícia Freitas Silva Chavarria, Marilyn Urrutia-Pereira, Nelson Augusto Rosário Filho, Norma de Paula Motta Rubini, Olavo Mion, Otávio Bejzman Piltcher, Regina Terse Ramos, Renata Di Francesco, Renato Roithmann, Wilma Terezinha Anselmo-Lima, Fabrizio Ricci Romano, João Ferreira de Mello Júnior

**Affiliations:** a Universidade Federal de São Paulo, Escola Paulista de Medicina, São Paulo, SP, Brazil; b Sociedade Brasileira de Pediatria, Rio de Janeiro, RJ, Brazil; c Associação Brasileira de Alergia e Imunologia, São Paulo, SP, Brazil; d Universidade do Estado do Rio de Janeiro, Rio de Janeiro, RJ, Brazil; e Universidade de São Paulo, São Paulo, SP, Brazil; f Universidade de Santo Amaro, São Paulo, SP, Brazil; g Universidade Federal do Paraná́, Curitiba, PR, Brazil; h Associação Brasileira de Otorrinolaringologia e Cirurgia Cérvico-Facial, São Paulo, SP, Brazil; i Universidade Federal do Rio de Janeiro, Rio de Janeiro, RJ, Brazil; j Universidade Estadual de Campinas, Campinas, SP, Brazil; k Faculdade de Ciências Médicas da Santa Casa de São Paulo, São Paulo, SP, Brazil; l Universidade Federal de Juiz de Fora, Juiz de Fora, MG, Brazil; m Fundação Universidade Federal de Ciências da Saúde de Porto Alegre, Porto Alegre, RS, Brazil; n Universidade Federal do Pampa, Uruguaiana, RS, Brazil; o Universidade Federal do Estado do Rio de Janeiro, Rio de Janeiro, RJ, Brazil; p Universidade Federal do Rio Grande do Sul, Porto Alegre, RS, Brazi; q Universidade Federal da Bahia, Salvador, BA, Brazil; r Universidade Luterana do Brasil, Canos, RS, Brazil; s Universidade de São Paulo, Faculdade de Medicina de Ribeirão Preto, Ribeirão Preto, SP, Brazil

**Keywords:** Rhinitis, Allergic rhinitis, Consensus, Epidemiology, Evidence-based medicine, Phenotypes, Treatment

## Abstract

Since we published the “IV Brazilian Consensus on Rhinitis”, in 2017, several advances have been achieved and have enabled a further understanding of the different aspects of “Rhinitis”. This new guideline, developed jointly by ASBAI, SBP and SBORL, represents a relevant milestone in the updated and integrated management of the different forms of the disease, and it aims to unify evidence-based approaches to improve the diagnosis and treatment of this common and often underestimated condition. The document covers a wide range of topics, including clear definitions of the different phenotypes and endotypes of rhinitis, risk factors, updated diagnostic criteria, and recommended methods for clinical and laboratory investigation. We stress the importance of detailed clinical history and objective assessment, as well as tools for control and assessing severity tools an accurate diagnostic approach to the disease. Regarding treatment, it emphasizes the treatment customization, considering the severity of symptoms, the presence of comorbidities and the impact on the patient's quality of life. We discuss different drug treatment, in addition to non-pharmacological measures, such as environmental control and specific immunotherapy; and the possible role of immunobiological agents. Furthermore, the consensus addresses issues related to patient education, prevention and management of special situations, such as rhinitis in children, in pregnant women and in the elderly. In short, the “V Brazilian Consensus on Rhinitis” represents a comprehensive and updated guide for healthcare professionals involved in the diagnosis and management of rhinitis, aiming to improve patients' quality of life through an integrated and evidence-based approach.

## Highlights

• This consensus brings together the most up-to-date information on rhinitis.
• Anamnesis is the key point in the suspected diagnosis and phenotype identification.
• It is especially important to recognize comorbidities.
• One aspect that impacts adequate treatment is low patient compliance.

## Development of Guidelines for the diagnosis and treatment of rhinitis

Medicine has made large strides towards obtaining more assertive knowledge in recent decades. Previously unknown information has been increasingly acquired, enhancing and making hitherto unknown clinical situations clearer.

Since the publication of the IV Brazilian Consensus on Rhinitis, in 2017, several advances have been achieved and have brought about a further understanding of the various aspects of “Rhinitis”, as well as its treatment.

Since then, new forms of rhinitis have been established: mixed, dual; and their endotypes have been better understood.

The V Brazilian Consensus on Rhinitis addresses these topics, distributed in sections that range from nasal anatomy and physiology, phenotypes and endotypes of rhinitis, allergic rhinitis, risk and protective factors for allergic rhinitis, clinical picture, diagnostic evaluation, comorbidities in addition to the most recent achievements in the treatment of these conditions.

## Nasal anatomy and physiology

### Nose

The first structure of the upper respiratory tract is the nose, the most anterior segment just above the hard palate. The nose can be divided into “external nose” and “nasal cavities”. These cavities, right and left, are made up of four walls, separated by the nasal septum and called nasal fossae. This structure represents great relevance in facial aesthetics, as it serves as the focal point of the face and it is often the first structure to which our eyes are drawn.^2^

The nose is also extremely important in respiratory dynamics, promoting olfaction, filtering and humidifying inhaled air, as well as eliminating secretions from the paranasal cavities and nasal lacrimal ducts.^1^, ^3^

#### External nose

The external nose projects from the face in a pyramid shape. Its skeleton is osteo-fibrocartilaginous based on the frontal and alveolar processes of the maxilla and nasal bones.

The cartilaginous portion of the external nose is formed by the greater and lesser alar cartilages that are connected to the septal cartilage (medial branch of the greater alar cartilage) on each side of the midsagittal plane.^1^

The shape and dimensions of noses vary widely due to differences in nasal cartilage. Thus, nasal defects present a unique challenge to the surgeon, as he/she must balance nasal shape with function.^2^

The alar cartilages are “*U*” shaped, free and mobile. These dilate or contract the nostrils with the action of nasal muscles.^3^

The “nasal flapping” sign frequently appears when there is respiratory effort in children and infants, since the movement of the alar cartilages and consequently that of the fibroadipose alar tissue are physiological attempts to reduce the resistance of the upper airway and the respiratory effort, aiming to increase tidal volume.^4^

The nasal dorsum extends from its superior angle or root to the nasal apex. The lower nasal surface is pierced by the nostrils (two peripheral openings), laterally outlined by the nostril wings and separated by the nasal septum. The bony part of the nose consists of the nasal bones, the frontal processes of the maxilla, the nasal part of the frontal bone, and its nasal spine.

The upper bony part of the nose, including its root, is covered with thin skin and the cartilaginous part is covered with thicker skin, with a large number of sebaceous glands. The skin extends to the nasal vestibule, where it has a variable amount of rigid hairs called vibrissae that form the first filtration barrier for inhaled air.^5^

The area outlined between the cartilaginous septum and the larger alar cartilages is called the external nasal valve and it is responsible for around 50% of respiratory resistance, causing different pressure gradients between the airway and the environment, thus having great importance in nasal obstruction.^6^, ^7^

Over the years, nasal volume and shape change due to the natural weakening of the cartilages that form the nasal framework, making the organ lower and resulting in nasal obstruction.^6^, ^7^

### Nasal cavity

The nasal cavity has a wide lower base, called the nasal floor, and a narrow upper base, called the nasal roof. These bases are opened anteriorly through the nostrils (right and left) and separated medially by the nasal septum.

The posterior limit of the nasal cavity is determined by the choanae, which have an oval shape, delimited laterally and posteriorly by the sphenoid bone and inferiorly by the vomer bone. These limits with the rhinopharynx and consequently with the tubal ostia and pharyngeal tonsil may be factors that explain nasal obstruction processes, auricular dysfunctions and infections associated with nasal diseases such as allergic rhinitis.^1^, ^5^

The lateral walls of the cavity have irregularities due to three bony sheets that project inferiorly, called nasal turbinates.

The lining of the nasal cavity is made up of a mucous membrane. This tunic is rigidly attached to the periosteum of the bones and cartilage that support the nose. Its lower two thirds correspond to the respiratory area and the upper third to the olfactory area.^1^, ^3^

Physiologically, air inspired through the nose passes through the nasal cavity, where it is warmed and humidified before passing from the rest of the upper respiratory tract to the lungs. When inhaling, the nostrils capture countless molecules dispersed in the air and carry them to this upper posterior area of the cavity, bringing odor and fragrance particles into contact with the receptors of the olfactory cells. This generates neural and sensory responses to odors and aromas.^2^, ^5^

#### Septum

The nasal septum is formed by a bony part and a mobile and flexible cartilaginous part. It is made up superiorly and posteriorly by the perpendicular plate of the ethmoid, inferiorly by the vomer and anteriorly by the septal cartilage, quadrangular cartilage and anterior spine. The septum divides the nasal cavity into two, right and left, and it is lined by the periosteum, submucosa and mucosa. It has rich vascularization, mainly in the anteroinferior region (Kiesselbach's plexus), whose involvement by diseases such as rhinitis can cause anterior epistaxis.^1^, ^5^

#### Lateral wall

The lateral nasal wall is most complex and important, made up of protrusions and depressions, with three primary structures: inferior, middle and superior nasal turbinates.

In a coronal view, the nasal turbinates have a medial or septal face ‒ the nasal septum, and a lateral or meatal face for the respective meatuses. They increase the volume of the nasal mucosa, regulating respiratory flow, affecting humidification, conditioning and filtration of inhaled air, before it is taken to the pulmonary alveoli.^2^, ^3^

The lower and middle nasal turbinates originate from the maxillary and ethmoid bones, respectively; and they are the most important in nasal physiology. They are anatomically divided in the anteroposterior direction: into head, body and tail. The upper turbinates originate from the ethmoid and sphenoid bones.

The meatuses are found in the lateral wall and form the meatal ostium complex. In the middle meatus we find the opening of the frontal and maxillary sinuses and anterior ethmoidal cells, in addition to the unciform process, the semilunar hiatus and the ethmoidal bulla; in the inferior meatus is the opening of the nasolacrimal duct.^1^, ^3^

#### Olfactory area

The olfactory system forms in the 28th week of gestation, making it the first sense to develop in the human species.^4^

The olfactory epithelium is located in the roof of the nasal cavity, in the upper portions of the septum and lateral wall.

The olfactory region normally occupies an area of one cm^2^ in each nostril. The central processes of the cells that make up the olfactory nerve pass through the cribriform plate of the ethmoid bone to reach the olfactory bulb.^1^, ^10^

Coming from this structure, nerve impulses are sent to the cerebral cortex with connections to the thalamus interrelated with taste stimuli.

Chronic nasal congestion, nasal polyposis and naso-septal deformities may be related to changes in smell and taste, as they can physically impair the contact between olfactory receptor cells and the olfactory bulb.^5^, ^6^

The sense of smell reaches its peak in the third and fourth decades of life, decreasing thereafter. It is estimated that its function is reduced in more than 50% of individuals aged between 65 and 80 years, and in more than 70% of those over 80 years of age. Multiple factors contribute to this olfactory loss, such as: cumulative lesions of the olfactory epithelium resulting from environmental changes (such as intense heat and cold and retention of pollution in the atmosphere), reduction of mucosal enzymes, loss of receptors that capture odorous particles, replacement of the olfactory epithelium by the respiratory epithelium with advancing age, changes in neurotransmitters and neuromodulators, and neuronal losses in the olfactory bulb, among others.^6^, ^10^

### Vasculature and innervation

The nasal cavity has a rich vascular array that is bilaterally brought about by the external and internal carotid arteries and their branches from the maxillary and ophthalmic arteries, respectively. The arterial irrigation of the medial and lateral wall of the nose has five main origins: (1) Anterior ethmoidal artery, (2) Posterior ethmoidal artery, (3) Sphenopalatine artery, (4) Greater palatine artery and (5) Septal branch of the superior labial artery and facial artery.^1^, ^5^

In the middle and lower nasal turbinates, cavernous plexuses are formed, capable of periodically modifying the shape and volume of the turbinates, unilaterally. This change in blood volume is known as the nasal cycle phenomenon and is determined by extrinsic or intrinsic stimuli: physicochemical, inflammatory, neurogenic and even psychogenic.^2^, ^5^

Nasal lymphatic drainage is performed to the retropharyngeal and subdigastric regions.

The predominant innervation is provided by the ophthalmic and maxillary branches of the trigeminal nerve. There is parasympathetic predominance in relation to the Autonomic Nervous System (ANS). Parasympathetic, sympathetic and sensory fibers are responsible for conducting – responding to stimuli from the nasal mucosa with adrenergic and cholinergic receptors.^1^, ^3^

As we age, nasal vascularization also changes. The microvasculature has a smaller caliber, smaller distribution and greater fragility, which can increase the occurrence of epistaxis. These changes contribute to nasal mucosa atrophy, sensation of dryness and nasal obstruction.^6^, ^10^

### Nasal mucosa and mucociliary barrier

The nasal mucosa is covered by ciliated cylindrical pseudostratified epithelium with goblet cells (mucus producers) and other types of cells supported on the basal lamina, such as: ciliated columnar cells; goblet cells; brush cells (brush cells-sensory receptors), basal cells and granule cells. There are also inflammatory cells, such as T and B lymphocytes, mast cells, monocytes, neutrophils, eosinophils and basophils.^11^, ^12^

The respiratory lining is thicker and heavily irrigated, rich in ciliated cells and with a large number of mucous glands that secrete around one liter of mucus per day, which is made up of 95% water, glycoproteins (sialomucin, fucomucin and sulfomucin), of enzymes (lysozyme and lactoferrin), immunoglobulins (IgA, IgG, IgM and IgE), cellular debris and slightly acidic pH. This epithelium has a large number of goblet cells and serumucous glands. Serous cells are involved in the production of fucomucins that make up the aqueous phase of mucus, or “sol phase”; while mucous glands produce sialomucins and sulfomucins that form the thick fluid of the “gel phase”.^5^, ^11^, ^12^

Mucociliary transport in the respiratory system occurs due to the viscoelasticity of the mucus, the ciliary beat in metachronous waves and the coupling between the cilia and the mucus, with the integrity of the epithelium as a preponderant factor for the effectiveness of this mechanism.

The ciliary beating mechanism occurs in two stages: effective beating (fully extended cilia promoting the propulsion of the gel phase) and recovery beat (moment of return to the initial position, close to the cell surface).

The functioning of mucociliary transport is of fundamental importance in the pathophysiology of rhinitis. Changes in ciliary mobility can be caused by several factors such as: inflammatory processes, acute and chronic infections, severe dehydration, use of topical or systemic medication, ciliary dyskinesia, cystic fibrosis, among others.^5^, ^12^

In advanced age there is a reduction in the number of submucosal glands with a consequent decrease in mucus, which makes the epithelium thicker and with a loss in the effectiveness of the mucociliary system. This causes nasal congestion and predisposition to respiratory tract infections.^6^, ^10^

#### Ultrastructure

Intracellular connections stand out among ultrastructure elements such as: adhesion zonules, desmosomes, hemidesmosomes, gap junctions and tight junctions. The latter, also known as zonules of occlusion or tight junctions, play a semi-impermeabilizing role in the epithelial paracellular space, functioning as a barrier separating the intra- and extracellular compartments. This maintains integrity and controls exchanges between these environments. This mechanism is essential for protecting the body from harmful agents.^5^, ^11^

The occlusion zonules are formed by proteins, occludins and claudins, susceptible to the proteolytic action of dust mite allergens, such as Der p 1 and various pollens. In allergic patients, the zonules of occlusion become looser in the connections among goblet cells and between goblet cells and hair cells, thus enabling dendritic cells to reach antigens and allow extravasation of intracellular fluid.

This mechanism may occur in inflammatory and infectious processes, in exposure to toxic substances and in conditions of hyperosmolarity.^5^, ^11^

### Airway and orofacial development

One of the main symptoms of rhinitis is nasal obstruction. Chronic or intermittent, results in oral replacement breathing. Inhaling air, preferably through the mouth, is not a physiological situation but a pathological one. In children, it influences craniofacial growth and development in a negative way.

The correct diagnosis and treatment of nasal obstruction, rhinitis and other diseases that cause it have a direct role in preventing facial disharmony, as well as dental malocclusion. In addition to early diagnosis, multidisciplinary intervention and management is necessary, in addition to a doctor (pediatrician, family doctor, otorhinolaryngologist, allergologist), dentist, speech therapist, physical therapist.

It has long been known that nasal obstruction results in increased nasal resistance to the passage of air and consequent oral breathing; and that sixty percent of craniofacial growth occurs during the first four years, and 90% by age.^12^, ^13^ Breastfeeding is an important physiological stimulus for the establishment of nasal breathing, given that lip sealing is essential to maintain the intra-oral vacuum, necessary for the extraction of breast milk, thus stimulating nasal breathing. Children who were not breastfed are 38% more likely to develop mouth breathing.^14^

Narrowing of the airway may be common in mouth-breathing children.^15^ These changes can negatively impact the aesthetic appearance, functional occlusion and stability of the craniofacial complex.

Facial development results from the interaction of several factors. Both systemic: genetic, endocrine, metabolic and behavioral; and local: teething, inappropriate habits (pacifier, thumb sucking, among others), muscular changes and breathing. According to Enlow,^16^ airway patency is key to facial development; and to the growth and development of the nasomaxillary complex. Airflow promotes bone reabsorption in the internal part of the nasal cavities and bone deposition in the external part, contributing to the lowering of the hard palate.

Mouth breathing is associated with several dental malocclusions, including anterior open bite, posterior crossbite, overjet, and increased tooth protrusion. Altered tongue posture and inadequate nasal breathing contribute to these malocclusions and abnormal tongue positioning, affecting the transverse development of the dental arches and causing future maxillary narrowing.^17^

A recent literature review with meta-analysis concluded that mouth breathing can alter craniofacial growth patterns, leading to skeletal changes, such as an elongated face, posterior rotation of the mandible and increased vertical facial dimensions. Their results showed that the mandible and upper jaw grow backwards and downwards, altering the occlusal plane. Furthermore, mouth breathing can increase lip protrusion and upper anterior teeth tilt.^15^

## Rhinitis

### Definition

Rhinitis corresponds to an inflammation of the nasal mucosa, characterized by the presence of symptoms such as itching, sneezing, rhinorrhea (anterior or posterior) and nasal obstruction or congestion. In patients who present associated conjunctival symptoms, it is called rhinoconjunctivitis.^18^, ^19^

### Classification

Nasal symptoms are nonspecific, and they are found in different phenotypes associated with rhinitis (Table 1). It is classified as infectious, allergic and non-allergic.^18^, ^19^, ^20^

**Table 1:** Rhinitis classification.^3^

Allergic rhinitis	Infectious rhinitis	Non-allergic rhinitis
“Systemic”	Viral	Drug-induced
Local	Bacterial	Non-allergic eosinophilic rhinitis
Dual	Fungal	Drug-induced
Mixed		Occupational
		Tobacco-induced
		Gestational
		Hormonal
		Food-induced
		Alcohol-induced
		Age-induced rhinitis
		Atrophic
		Empty nose syndrome
		Autoimmune, granulomatous and vasculitis rhinitis

Infectious rhinitis can be of viral, bacterial or fungal etiology. In addition to nasal symptoms, patients may present with cough, fever and general malaise. The main agents in viral cases are rhinovirus, adenovirus, influenza virus and parainfluenza, while in bacterial cases Streptococcus pneumoniae, Hemophilus influenza and Moraxella catarrhalis predominate.^20^

Allergic Rhinitis (AR) is a type I hypersensitivity reaction, mediated by Immunoglobulin E (IgE), of the nasal mucosa resulting from exposure to allergens in a sensitized individual.^20^ Although it has already been classified into seasonal and perennial, according to the type of triggering antigen, currently subdivided into intermittent ‒ when symptoms occur for less than four days a week and less than four weeks a year; or persistent ‒ if they occur for more than four weeks and more than four days in a row, regardless of the associated allergen.^21^ Currently its severity is assessed using a visual analogue scale from 0 to 10. A score greater than 5 is considered an uncontrolled disease, 3–5 is considered partially controlled and less than 2 is considered controlled.^22^

Some phenotypes are observed in patients with AR. In local AR, specific IgE testing using the immediate hypersensitivity skin test and specific serum IgE quantification are negative. That is, these patients only present local (nasal) production of specific IgE. The gold standard for confirming this diagnosis is the nasal provocation test. Another phenotype is dual AR, where the patient simultaneously presents with local AR for a given antigen and “systemic” sensitivity to other antigens. Finally, some patients present mixed allergic rhinitis corresponding to the association of AR with non-allergic rhinitis.^3^, ^20^, ^24^

Around 30%–50% of allergic patients present nasal symptoms, both when exposed to allergens and non-allergic triggers, with their rhinitis being called mixed allergic rhinitis. In them, in addition to the IgE-mediated pathway, we have a hyperreactivity of neurogenic pathways. The main non-specific agents are temperature changes, cigarette smoke, perfumes and cleaning products.^25^

In non-allergic rhinitis we find several mechanisms that may or may not be related to inflammation of the nasal mucosa.^20^

In drug-induced rhinitis, one of the mechanisms is Non-Allergic Eosinophilic Rhinitis (NAER), which occurs with the use of non-steroidal anti-inflammatory drugs. Another is neurogenic rhinitis; as it occurs with alpha- and beta-adrenergic blockers, phosphodiesterase inhibitors, angiotensin converting enzyme inhibitors and illicit drugs, such as cocaine, which generate nasal symptoms without the presence of inflammation. Finally, we have drug -induced rhinitis, which is a consequence of the regular and continuous use of topical nasal vasoconstrictors.^20^

Inflammatory rhinitis related to the workplace is considered occupational. They may or may not be of allergic etiology. Proteins with a molecular weight greater than 5 kDa can cause immunological sensitization, while those with a lower molecular weight cannot. Non-allergic mechanisms are irritation or inhalation of corrosive products that can lead to ulcers and septal perforation. Occupational rhinitis deserves special attention, as it precedes the appearance of occupational-related lower airway symptoms.^20^

Irritating factors such as exposure to cigarette smoke, cigar smoke, pipe smoke, electronic cigarette smoke can trigger nasal symptoms.^20^

Gestational rhinitis is one which symptoms appear during pregnancy and disappear when it ends. It should not be confused with hormonal rhinitis such as that which occurs in hypothyroidism.^20^

Some cases of rhinitis are associated with food and alcohol, but not due to an allergic mechanism. Gustatory rhinitis is characterized by unilateral or bilateral watery rhinorrhea after the patient consumes foods containing pepper-based seasonings (capsaicin), when neurogenic receptors are stimulated, triggering a runny nose. Alcohol intake can be a trigger for nasal symptoms, especially in patients with Aspirin-Exacerbated Respiratory Disease (AERD).^20^

With the population aging, rhinitis must be considered in this population. Hormonal, neurogenic and histological changes are physiological in this age group and can generate symptoms, such as rhinorrhea, nasal congestion, changes in smell, dry nose and formation of local crusts.^20^

Other causes of nasal symptoms are atrophic rhinitis, empty nose syndrome, rhinosinusitis, granulomatous rhinitis, vasculitis and autoimmune diseases.^20^

Several conditions present nasal symptoms that are not considered rhinitis. Anatomical changes such as septal deviation, turbinate hypertrophy and nasal valve changes, tumors, hemangiomas, mucoceles, encephaloceles, among others, stand out.^20^

## Phenotypes and endotypes of rhinitis

### Pathophysiology

#### IgE-mediated rhinitis

AR is an inflammatory condition of the nasal mucosa in genetically predisposed individuals, as a response to IgE-mediated reactions to environmental allergens, activating type 2 cells. AR is a chronic inflammation, characterized by sneezing, itching, nasal congestion and rhinorrhea. Clinically it is classified according to severity, frequency and seasonality.^26^

The inflammatory reaction is triggered by allergens from house dust mites, cockroaches, animal hair, fungi and pollens, being associated with changes in the epithelial barrier of the nasal mucosa, activating dendritic cells that present the peptides from the allergens to naïve CD4 T cells, initiating the sensitization response to the antigen, characterized by the production of IL-4, IL-5, IL-13 and IL-31. These cytokines promote the differentiation of B lymphocytes into plasma cells, producing memory allergen-specific IgE, which will bind to high-affinity IgE receptors (FcεRI), located on the membrane of mast cells and basophils. These processes form a pool of Th2 memory cells and allergen-specific B cells.

**Fig. 1:**
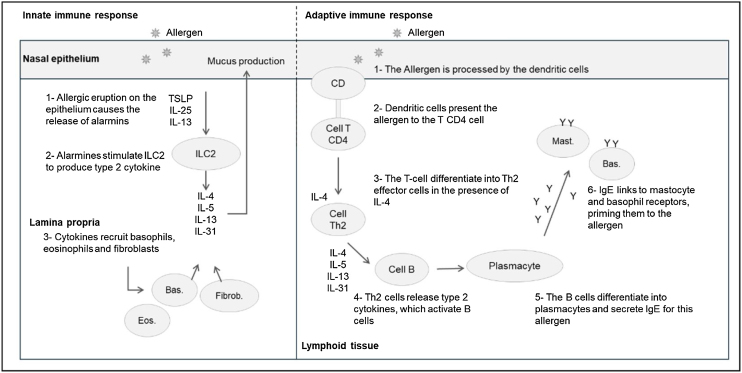
Mechanisms of the innate and adaptive allergic rhinitis response activation. TSLP, Thymic Stromal Lymphopoietin; IL, Interleukin; ILC2, Group 2 innate lymphoid cells; Eos, Eosinophil; Bas., Basophil; CD, Dendritic Cells. Adapted from Berstein JA.^28^

Disruption of the epithelial barrier of the nasal mucosa results in the production of alarmins (IL-25, IL-33, TSLP ‒ Thymic Stromal Lymphopoietin) that activate type 2 Innate Cells (ILC2); which will also contribute to the production of pro-inflammatory agents (IL-4, IL-5, IL-13, IL-31) (Fig. 1).^27^

**Fig. 2:**
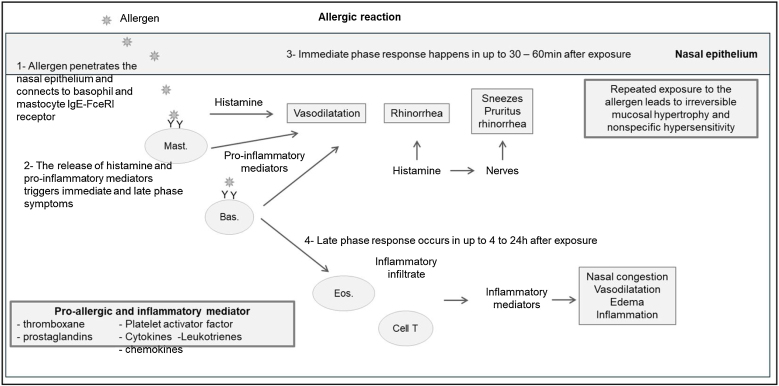
Systemic mechanisms; allergic rhinitis comorbidities and clinical manifestations. Mast., Mastocytes; Bas, basophil; Eos, eosinophil; Cell T, T-Cells. Adapted from Berstein JA.^28^

Re-exposure to the allergen promotes cross-linking between specific IgE molecules and high-affinity receptors (FcεRI) that result in the degranulation of mast cells and basophils, release of pre-formed mediators (histamine) and newly formed mediators (prostaglandins, leukotrienes, platelet activating factor) promoting the allergic reaction (mediate and late phase), with vasodilation and inflammatory infiltrate of mast cells, eosinophils and basophils^28^, ^29^ (Fig. 2).

#### Local IgE production

Local AR involves a nasal allergic response in patients with negative immediate hypersensitivity skin tests and no specific serum IgE to aeroallergens.

The characteristic pathogenesis is the production of antigen-specific IgE in the nasal mucosa, with a T2 pattern in the inflammatory infiltrate of eosinophils, basophils, mast cells, CD3 and CD4 lymphocytes, as well as the release of inflammatory mediators (tryptase and eosinophil cationic protein) during natural exposure to aeroallergens and as a response to the nasal provocation test with allergens (house dust mites and pollens).^30^, ^31^

Mast cells and eosinophils from patients with local allergic rhinitis show immediate activation in the nasal mucosa, releasing inflammatory mediators such as tryptase and eosinophilic cationic protein.^32^

Nasal lavage assessment by flow cytometry confirms the presence of Th2 IgE-mediated inflammation.^33^

#### Non-IgE-mediated inflammation

Non-Allergic Rhinitis (NAR) involves a variety of pathophysiological conditions, and it can be broken down into a classical inflammatory pathway, a neurogenic pathway, and other, unknown, pathways. It is possible that these pathways also contribute to inflammation of the nasal mucosa in AR.^34^, ^35^, ^36^

NAR is defined as a rhinitis with a non-IgE-mediated mechanism that includes idiopathic rhinitis (previously also called vasomotor rhinitis), infectious rhinitis, food-induced rhinitis, hormonal rhinitis, drug-induced rhinitis, non-allergic occupational rhinitis, atrophic, Eosinophilic Non-Allergic Rhinitis (ENAR) and rhinitis in elderly patients.^37^

ENAR, is a term used to describe a series of cases of non-asthmatic patients who reported perennial and intermittent nasal symptoms, and who have elevated nasal eosinophils (>20%), but with an absence of specific IgE in skin or serum tests. Some speculate that ENAR may precede the onset of Chronic Rhinosinusitis (CRS), asthma, or respiratory disease exacerbated by Nonsteroidal Anti-Inflammatory Drugs (NSAIDs).^38^

The ENAR syndrome continues to be highlighted in the North American guideline, updated in 2020, particularly due to its good response to treatment with intranasal topical corticosteroids.^38^

Some rhinitis phenotypes are based on a relatively simple regulatory disorder, such as rhinitis in the elderly, which appears to result mainly from parasympathetic/sympathetic neural imbalance, with a predominance of the first system, leading to significant rhinorrhea and nasal obstruction.^34^, ^35^, ^36^, ^37^, ^38^, ^39^

Idiopathic rhinitis is believed to be a disorder of the Non-Adrenergic and Non-Cholinergic system (NANC), also called the peptidergic neural system. Sensory nerve fibers contain neuropeptides, including Vasoactive Intestinal Peptide (VIP), P Substance (PS), and Calcitonin Gene-Related Peptide (CGRP).^34^, ^35^, ^36^, ^37^, ^38^, ^39^

There is a subgroup of patients with ENAR that have a mucosal inflammatory pattern. However, several patients with ENAR do not present influx of cells to the nasal mucosa and it is believed that a neurogenic mechanism is involved.

**Fig. 3:**
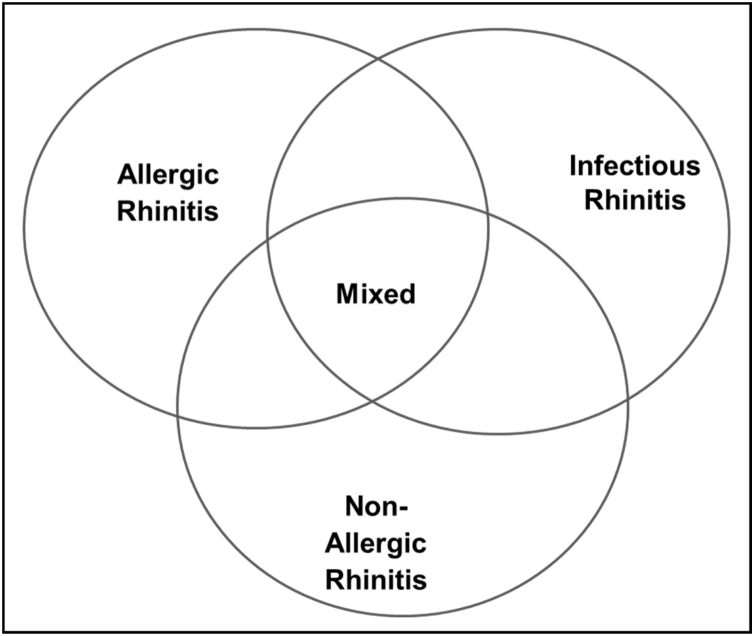
Types of rhinitis according to etiology.

Predominant rhinorrhea (sometimes called cholinergic rhinitis) indicates increased glandular secretory activity that can be effectively reduced with the use of atropine and ipratropium bromide.^40^ Patients with predominant symptoms of nasal congestion appear to have nociceptive neurons that show increased sensitivity to non-specific stimuli, such as changes in temperature, odors, irritants and ingestion of alcoholic beverages.^41^, ^42^ Fig. 3 shows the different types of rhinitis according to its etiology.

#### Inflammatory cellular infiltrate

Various approaches have been used to objectively monitor nasal inflammation, investigate disease mechanisms, and evaluate the effect of treatment interventions. These include nasal lavage, nasal cytology and nasal biopsy, together with the measurement of the nasal concentration of Nitric Oxide (NO).^43^

Nasal lavage is simple and quick to perform, it is well tolerated, and a sample of this material can provide information about the recruitment of luminal cells, cell activation and the extravasation of plasma proteins.^44^

The nasal mucosa, due to the ease of obtaining samples, allows the study of cellular changes during the allergic reaction. The extent of epithelial damage in several types of rhinitis and its correlation with inflammatory cells and mediators are not yet fully understood. The presence of eosinophils is associated with loss of epithelial integrity in patients with AR or ENAR.^45^, ^46^ The increase in the number of eosinophils in the nasal mucosa is the parameter that best correlates with the symptom of nasal obstruction.^47^

Surgical samples of nasal mucosa from patients diagnosed with RA and ENAR who underwent allergic evaluation, nasal lavage and histopathological analysis of tissues showed a cut-off point for the number of eosinophils in the nasal lavage of 4% for atopy differentiation. Some degree of epithelial injury was more frequent in patients with AR (94%) than in patients with ENAR.^48^

Activated eosinophils cause epithelial damage and loss of epithelial integrity in patients with rhinitis. Although there are some differences in the inflammatory process of the nasal mucosa, structural changes may be similar among patients with rhinitis, regardless of allergic status.^49^

AR is an excellent model for studying allergic inflammation, where triggering factors can be clearly identified, particularly in individuals with seasonality; and nasal symptoms can be monitored during and outside the pollen season.^37^, ^50^

Given the contribution of airway remodeling to the development and persistence of symptoms in airway disease, redirecting remodeling is an important treatment consideration. Tissue repair is driven by the migration of epithelial cells to the site of inflammation and activation of eosinophils.^51^, ^52^

#### Network of cytokines and soluble mediators

The key cell membrane receptors that define the unique biology of eosinophils include CCR3, which binds eotaxins, lectin (carbohydrate binding protein), Siglec-8, which can trigger eosinophil apoptosis when activated, and IL-5RA. Eosinophils also express receptors for multiple other cytokines and growth factors, including IL-4, IL-13, IL-33, TSLP, and TGF-β.^53^, ^54^, ^55^

Th2 and ILC2 immune cells produce cytokines, which cause a series of pro-inflammatory reactions, but also reactions that have the opposite, anti-inflammatory effect. The resulting inflammatory process will depend on the balance of these cytokines and biomolecules. A study involving adolescents with AR analyzed nasal secretions collected and tested for 13 cytokines using multiplex flow cytometry. The study identified that IL-1β, IL-6, as well as severe clinical manifestations, were predictors of lower quality of life in these patients, illustrating how cytokines of the allergic immunological reaction, in addition to providing inflammation, have other repercussions in individuals with AR.^56^

Evidence extrapolated from studies of the bronchial epithelium in asthma suggests that epithelial cells secrete TSLP, IL-33, IL-25 and other cytokines and chemokines that affect ILC2 and Th2 lymphocytes directly or via interaction with Antigen-Presenting Cells (APCs) located within and under the nasal epithelium.^57^, ^58^, ^59^

ILC2 express CRTh2, CD127 (IL-7 receptor) and ST-2, the receptor for IL-33. These cells preferentially express Th2 cytokines, particularly IL-5 and IL-13, and have the potential to enhance local allergy induced by T2 inflammation. T-cells activated during allergic inflammation differentiate into allergen-specific effector memory Th2 cells that release IL-4, IL-5, IL-9, and IL-13.^60^

The Nasal Provocation Test (NPT) with allergen represents an in vivo experimental model that has contributed immensely to the understanding of the AR mechanisms, enabling the evaluation of the two phases of the allergic reaction (immediate and late), by collecting nasal secretion with determination of mediator chemicals originating from activated mast cells (tryptase, histamine, prostaglandins, etc.).^61^, ^62^, ^63^ Nasal provocation with inhalable allergens can be safely conducted in specialists' offices.^64^

Based on a systematic review on challenge with nasal allergens, the European Academy of Allergy & Clinical Immunology (EAACI) reviewed evidence, variations in subjective and objective evaluation parameters to propose a standardized way of performing this procedure in clinical practice.^65^

An index that combines specific IgE activity for rPhl p 5 and nCyn d 1, visual analogue scale and skin prick tests, enabled predicting, with moderate sensitivity and high specificity, the result of NPT with grass pollen extract in complex pediatric patients and polysensitized with seasonal AR.^66^

#### Neural mechanisms

Neural mechanisms, including sensory nerves, sympathetic and parasympathetic nervous systems, play a crucial role in AR pathophysiology. These mechanisms work together to form a protective barrier in the upper airway mucosa, regulating the epithelium, glandular secretion and vascular processes.^20^

Recent studies on the neuro-immunological axis in the airway reveal the importance of the interaction between the immune system and the nervous system in regulating inflammation in the nasal mucosa. Cytokines produced in response to allergens transmit signals to the central nervous system, which induce the release of neuropeptides and neurotransmitters, stimulating neurons through axonal reflexes. Subsequently, neuropeptides and neurotransmitters impact various immune cells, resulting in the generation and release of inflammatory mediators, including cytokines, lipid mediators, and histamine. Furthermore, inflammatory mediators reduce the threshold for neuronal activation in response to stimuli. This neuro-immunological interaction establishes a positive feedback cycle, which results in the activation of neurons in response to low thresholds or non-harmful stimuli, intensifying neuronal activity at the inflammatory site.^35^

Nociceptors are specialized sensory neurons that detect and respond to harmful or potentially harmful stimuli, such as thermal, mechanical and chemical stimuli. Additionally, nociceptors are afferent nerves highly susceptible to stimulation during an acute allergic reaction. These nerve endings located in the nasal cavity are the peripheral processes of primary sensory neurons based in the trigeminal ganglion. In general, human nasal nociceptors are C fibers, which are typically sensitive to chemical and physical stimulation. These nerve endings express several receptors and ion channels, including the Transient Receptor Potential (TRP) ion channels – TRP subfamily V member 1 (TRPV1) and TRP subfamily A member 1 (TRPA1), receptors G Protein-Coupled Channels (GPCRs), acid-sensitive ion channels, mechanosensitive channels, voltage-gated ion channels and purinergic receptors, which convert environmental signals into electrical signals.^36^

Depolarization of nociceptive channels in sensory nerves leads to the release of neuropeptides, including substance P, CGRP, and neurokinin-A. Substance P receptors are located in the nasal epithelium, glands, venous, arterial and sinusoid vessels, leading to glandular secretion, increased vascular permeability, edema, vasodilation and additional activation of inflammatory cells. Substance P is a short-acting vasodilator, while CGRP is a long-acting arterial vasodilator. Neurokinin-A has similar effects, causing an increase in vascular permeability and vasodilation.^1^ Substance P, CGRP and neurokinin-A activate mast cell degranulation, leading to the enhancement of the release of inflammation mediators.

Evidence extrapolated from studies of the bronchial epithelium in asthma suggests that epithelial cells secrete TSLP, IL-33, IL-25 and other cytokines and chemokines that affect ILC2 and Th2 lymphocytes directly or via interaction with Antigen-Presenting Cells (APCs) located within and under the nasal epithelium.^57^, ^58^, ^59^

ILC2 express CRTh2, CD127 (IL-7 receptor) and ST-2, the receptor for IL-33. These cells preferentially express Th2 cytokines, particularly IL-5 and IL-13, and have the potential to enhance local allergy induced by T2 inflammation. T-cells activated during allergic inflammation differentiate into allergen-specific effector memory Th2 cells that release IL-4, IL-5, IL-9, and IL-13.^60^

The Nasal Provocation Test (NPT) with allergen represents an in vivo experimental model that has contributed immensely to the understanding of AR mechanisms, enabling the evaluation of the two phases of the allergic reaction (immediate and late), by collecting nasal secretion with determination of mediator chemicals originating from activated mast cells (tryptase, histamine, prostaglandins, etc.).^61^, ^62^, ^63^ Nasal provocation with inhalable allergens can be safely conducted in specialists' offices.^64^

Based on a systematic review on challenge with nasal allergens, the European Academy of Allergy & Clinical Immunology (EAACI) reviewed evidence, variations in subjective and objective evaluation parameters to propose a standardized way of performing this procedure in clinical practice.^65^

An index that combines specific IgE activity for rPhl p 5 and nCyn d 1, visual analogue scale and skin prick tests, allowed predicting with moderate sensitivity and high specificity the result of NPT with grass pollen extract in complex pediatric patients and polysensitized with seasonal AR.^66^

#### Neural mechanisms

Neural mechanisms, including sensory nerves, sympathetic and parasympathetic nervous systems, play a crucial role in the pathophysiology of AR. These mechanisms work together to form a protective barrier in the upper airway mucosa, regulating the epithelium, glandular secretion and vascular processes.^20^

Recent studies on the neuro-immunological axis in the airway reveal the importance of the interaction between the immune system and the nervous system in regulating inflammation in the nasal mucosa. Cytokines produced in response to allergens transmit signals to the central nervous system, which induce the release of neuropeptides and neurotransmitters, stimulating neurons through axonal reflexes. Subsequently, neuropeptides and neurotransmitters impact various immune cells, resulting in the generation and release of inflammatory mediators, including cytokines, lipid mediators, and histamine. Furthermore, inflammatory mediators reduce the threshold for neuronal activation in response to stimuli. This neuro-immunological interaction establishes a positive feedback cycle, which results in the activation of neurons in response to low thresholds or non-harmful stimuli, intensifying neuronal activity at the inflammation site.^35^

Nociceptors are specialized sensory neurons that detect and respond to harmful or potentially harmful stimuli, such as thermal, mechanical and chemical stimuli. Additionally, nociceptors are afferent nerves highly susceptible to stimulation during an acute allergic reaction. These nerve endings located in the nasal cavity are the peripheral processes of primary sensory neurons based in the trigeminal ganglion. In general, human nasal nociceptors are C fibers, which are typically sensitive to chemical and physical stimulation. These nerve endings express several receptors and ion channels, including the Transient Receptor Potential (TRP) ion channels – TRP subfamily V member 1 (TRPV1) and TRP subfamily A member 1 (TRPA1), G-Protein Coupled Channel-Receptors (GPCRs), acid-sensitive ion channels, mechanosensitive channels, voltage-gated ion channels and purinergic receptors, convert environmental signals into electrical signals.^36^

Depolarization of nociceptive channels in sensory nerves leads to the release of neuropeptides, including substance P, CGRP, and neurokinin-A. P-substance receptors are located in the nasal epithelium, glands, venous, arterial and sinusoid vessels, leading to glandular secretion, increased vascular permeability, edema, vasodilation and additional activation of inflammatory cells. The P-substance is a short-acting vasodilator; while CGRP is a long-acting arterial vasodilator. Neurokinin-A has similar effects, causing an increase in vascular permeability and vasodilation.^1^ P-substance, CGRP and neurokinin-A activate mast cell degranulation, leading to enhanced release of inflammatory mediators and amplification of the hypersensitivity reaction. Animal studies have demonstrated that CGRP can induce Th2 differentiation through direct effects on ILC2.^36^

The sympathetic nervous system releases norepinephrine, which is a potent vasoconstrictor, and Neuropeptide Y (NPY). NPY-secreting nerve fibers are located in arteries and veins, with a small number located in epithelium and glands. This distribution suggests that NPY acts in the regulation of blood flow, enhancing the vasoconstrictive effects of norepinephrine. The sympathetic nervous system maintains vascular tone under normal conditions and counterbalances the vasodilatory and pro-inflammatory effects of neurotransmitters and neuropeptides released by the parasympathetic and sensory nerves.^35^

The parasympathetic nervous system releases acetylcholine and VIP, which lead to increased mucous secretion, vasodilation and activation of epithelial cells.^20^ Acetylcholine exerts its actions via muscarinic receptors and VIP via two subtypes of receptors – VIP type 1 receptor (VPAC1) and VIP type 2 receptor (VPAC2). Recent studies have identified a new neuro-immunological axis – the VIP-prostaglandin D2 receptor axis, which is called Chemoattractant Receptor-Homologous molecule expressed on TH2 cells (CRTH2), involved in the recruitment of eosinophils. Studies in patients with allergic rhinitis have documented that stimulation of the nasal cavity leads to increased VIP content and recruitment of eosinophils via the CRTH2 receptor.^67^

Neuromedin U (NMU), derived from cholinergic neurons, acts as a regulator of type 2 cytokines. Its receptor is expressed on T-cells, dendritic cells, eosinophils and mast cells. NMU promotes mast cell degranulation and plasma extravasation. A recent study in patients with AR demonstrated that NMU activates ILC2, triggering the type 2 inflammatory response. These findings indicate that NMU can exacerbate the allergic inflammatory response and induce nasal hyperresponsiveness.^68^, ^69^

Nerve Growth Factor (NGF) was the first member of the neurotrophin family to be discovered. The main function of NGF is to promote the growth, differentiation and survival of peripheral and central nerves. NGF is expressed in the nasal epithelium, glandular epithelium and peripheral nerves of the nasal mucosa, playing a critical role in bidirectional signaling mechanisms between the network of neurosensory structures and immune cells. In patients with AR, NGF levels are elevated in the nasal submucosa, submucosal glands, nasal fluids, and serum. In addition to neurons, immune cells including mast cells and eosinophils are also sources of NGF, which can act on nasal hyperresponsiveness in allergic rhinitis, increasing the concentration of sensory nerves and neuropeptides, resulting in an increase in the inflammatory response.^68^

Table 2 summarizes the neural mechanisms involved in the pathophysiology of allergic rhinitis.^67^

**Table 2:** Neural mechanisms in the pathophysiology of allergic rhinitis.^67^

Nerves involved	Mediators	Effects
Sympathetic Nervous System	Noradrenalin Y	Vasoconstriction
	Neuropeptide Y	
Parasympathetic nervous system	Acetylcholine	Vasodilation
	VIP	Eosinophils recruitment
Sensorial nerves and neuroendocrine cells	P Substance	Vasodilatation
	CGRP	Plasma overflow
	Neurokinin A	Glandular secretion
		Release of pro-inflammatory cytokines
		Immune cells recruitment

VIP, Vasoactive Intestinal Peptide; CGRP, Calcitonin Gene-Related Peptide.

Nasal hyperresponsiveness, observed in 60%–70% of patients with AR is associated with changes in nasal innervation, including afferent nerves (somatosensory system) and efferent nerves (sympathetic or parasympathetic). Nasal symptoms, including sneezing, rhinorrhea, itching and obstruction, can occur in nasal hyperresponsiveness in response to various stimuli, such as cold air, capsaicin and hyperosmolar saline.^68^, ^69^, ^70^, ^71^

#### Epithelial and histological changes

The nasal mucosa acts as an air conditioner that regulates the temperature of the inhaled air, humidifying and cleaning the inspired air. The epithelium of the healthy nasal mucosa is made up of ciliated and non-ciliated columnar cells, mucus-secreting goblet cells, and basal cells that represent 50%–90% of the epithelial cell population. The epithelium rests in the nasal membrane area and covers the submucosal structures, forming a link between environmental exposure and the host's immune system. The nasal submucosa comprises serous, mucous and seromucous glands, extensive vascular and neural networks and components of the cellular and extracellular matrix.^20^, ^28^, ^72^

The nasal mucosa acts as a barrier against external pathogens and has antimicrobial, antioxidant and antiprotease properties. The nasal epithelium is covered by a mucus blanket, which consists of water, mucin glycoproteins, and antimicrobial peptides (lactoferrin, lysozyme, and defensins). The mucus blanket forms a double layer, composed of an inner serous layer and an outer viscous layer. The main components of nasal mucus are mucins, which play a significant role in anti-inflammatory and antimicrobial defense processes, as well as mucociliary clearance. During inflammation, mucociliary clearance is compromised, leading to excessive mucus collection that manifests as increased nasal and post-nasal secretion. Parasympathetic stimulation results in increased mucus production by the glandular cells of the nasal mucosa and leads to increased nasal secretion and nasal obstruction.^20^, ^28^

Inflammation of the nasal epithelium in response to allergens is the hallmark of AR. The histological characteristics of airway inflammation are goblet cell hyperplasia, mucus hypersecretion, thickening of the basement membrane and smooth muscle hyperplasia. In AR, this inflammatory response translates into mucosal edema, increased nasal secretion and nasal hyperresponsiveness. Exposure to inhalable allergens triggers increased production of chemical mediators and cytokines in the nasal epithelium. Studies in patients with AR demonstrate increased expression of pro-inflammatory cytokines and type 2 cytokines in the nasal mucosa.^20^, ^72^, ^73^, ^74^

As previously mentioned, AR is an excellent model for studying allergic inflammation, as triggering factors can be clearly identified, particularly in intermittent forms related to pollen, which can be monitored and studied during and outside the pollen season. Nasal secretion and mucous tissue are easily accessible for invasive and non-invasive procedures, enabling the analysis of the immunological and clinical response to allergens. Allergen challenge results in tissue eosinophilia and an increase in cells expressing Th2 cytokines. Studies have documented an increase in Th2 transcriptional factors, such as STAT6+ and GATA3+, in the nasal mucosa of patients with allergic rhinitis when compared to healthy controls.^28^

Studies of nasal wash samples from patients with pollen allergy have demonstrated distinct patterns of gene expression profiles and functional genetic pathways that indicate anatomical and functional origins. Mucin production, regulated by the MUC5AC and MUC5B genes, is upregulated upon exposure to allergens. Goblet cell hyperplasia is particularly related to the high expression of CD44v3, which is a marker of intermediate progenitor cells of the basal layer.^20^

Immunohistochemistry of nasal turbinate biopsies obtained from patients with AR six hours after challenge or during natural exposure to allergens reveals increased expression of the lymphocyte chemokine receptors CCR3, CCR4, eosinophil infiltration, and elevated levels of cells expressing mRNA for IL-4 and IL-5. Cytokines released by mast cells, basophils and Th2 cells such as IL-4, IL-5, IL-9 and IL-13 play a key role in the late phase of AR. Studies have shown that there is an inverse correlation between IL-5 and IL-13 with post-challenge nasal patency. Eosinophilic mediators, such as Major Basic Protein (MBP), Eosinophilic Cationic Protein (ECP) and Eosinophilic Peroxidase (EPO) are toxic to the respiratory epithelium, promoting increased oxidative stress and causing tissue injury and damage. This epithelial damage, in turn, leads to the release of cytokines derived from the epithelium, the so-called alarmins, cytokines and growth factors that maintain and amplify the allergic inflammatory response.^28^, ^72^

The amplification and chronicity of the inflammatory response in the nasal mucosa has the potential to lead to structural histological changes. Studies have investigated the mesenchyme-epithelium transition in allergic rhinitis since changes in this process are related to remodeling in asthma. However, no changes in epithelial differentiation were detected in terms of expression of E-cadherin, cytokeratin, MUC5A+, goblet cells and p63+ basal cells. Studies that explored immunohistochemical evidence of remodeling in patients with severe persistent AR did not identify morphological changes in the epithelium and basement membrane, and some changes in relation to glandular changes, angiogenesis and lymphangiogenesis.^20^, ^28^, ^75^

#### Changes in the epithelial barrier

The epithelial barrier is the first line of defense in the nasal mucosa, and it is essential that this barrier is intact to protect against pathogens, harmful agents and allergens. The epithelium plays a vital role in regulating the innate and adaptive immune response by activating functional molecules that participate in the inflammatory response (e.g., pro-inflammatory cytokines, growth factors and chemokines). Epithelial cells secrete antimicrobial substances, known as antimicrobial peptides, including lysozyme, defensins (α e β), lactoferrin and S-100 proteins.^76^

Epithelial barrier dysfunction has been related to chronic inflammatory diseases in multiple organs and systems, with emphasis on allergic diseases such as atopic dermatitis, asthma and chronic rhinosinusitis. Several studies have investigated the role of the epithelial barrier in allergic rhinitis. The defective epithelial barrier can facilitate the entry of allergens and pathogens into the nasal mucosa, perpetuating the inflammation of allergic rhinitis.^20^, ^77^

The barrier between sinonasal epithelial cells is formed via interactions of apical transmembrane and cytoskeletal proteins, including tight junction proteins, such as Zonula Occlusion-1 (ZO-1), members of the Claudin (CLDN), Occludin (OCLN), and Junctional Adhesion Molecules-A (JAM-A) families. Adherens junction proteins, such as cadherins, create intercellular interactions. Desmosomes and hemidesmosomes also participate in apical junctional complexes. Taken together, the function of these junction proteins is to limit the passage of intercellular fluid and protect the underlying tissue against exposure to harmful agents and allergens.^78^, ^79^

Tight junctions are more apically located and include more than 40 proteins composed of transmembrane proteins or cytoplasmic actin-binding proteins, which function is to regulate the homeostasis of ions, water and some macromolecules. Adherens junctions are essential for cell proliferation, differentiation and adhesion. Desmosomes are intricately connected to adherens junctions and play a key role in maintaining cellular integrity and intercellular cohesion. Finally, hemidesmosomes are responsible for facilitating the stable adhesion of basal epithelial cells to the basement membrane.^76^

Several mechanisms of epithelial barrier disruption in AR have been described, including the proteolytic activity of allergens, environmental factors, and dysfunction mediated by inflammatory cytokines. Household dust mites are the most common etiological allergens in AR in our country and, among the main species, are Dermatophagoides pteronyssinus (Der p) and Dermatophagoides farinae (Der f). It has been described that Der p1 has the property of cleaving sites in the extracellular domains of CLDN1 and OCLN proteins, resulting in increased cellular permeability that allows Der p1 to pass through the epithelial barrier. Breakdown of the epithelial barrier can also be caused by proteases via exposure to fungi, facilitating the access of pathogens and direct activation of immune cells. Serine proteases from fungi such as Alternaria spp. have the ability to reduce the expression of tight junction proteins such as ZO-1, OCLN and CLDN1.^20^, ^76^, ^78^

Environmental factors such as living in urban regions, air pollution and indoor pollutants can also contribute to the disruption of tight junctions in AR. A study that evaluated patients with AR documented low levels of OCLN and CLDN7 expression; associated, respectively, with residence in urban regions and exposure to passive smoking. Air pollution represents a risk factor for the onset of AR, due to the compromise of the epithelial barrier by Diesel Exhaust Particles (DEPs) and fine Particulate Matter (PM 2.5). Particulate matter contains redox-active chemicals and transition metals and can have disruptive effects through the generation of reactive oxygen species. Study in animal models (mice) demonstrated that PM 2.5 reduces the expression of ZO-1.^20^, ^76^, ^77^, ^78^

The onset of AR can also be triggered by aggression and disruption of the sinonasal epithelium, through the production of alarmins (TSLP), IL-25 and IL-33. These cytokines are key regulatory factors in epithelial-mesenchymal communication and elicit pathological changes in the airway. Receptors expressed on the surface of epithelial cells, Toll-Like Receptors (TLRs) and Nucleotide-binding Oligomerization Domain (NOD)-Like Receptors (NLRs) have the ability to identify Pathogen-Associated Molecular Patterns (PAMPs) in microorganisms and induce innate and adaptive response.^76^, ^79^

On the other hand, the type 2 cytokines IL-4 and IL-13 not only participate in the allergic inflammatory response, but also regulate the epithelial barrier in allergic diseases, resulting in a reduction in the expression of tight junction proteins in epithelial cells.^76^, ^79^

Studies conducted in patients with AR that evaluated mRNA expression in nasal biopsy demonstrated reduced expression of CLDN 1, 4, 7, 8, 12, 13 and 14. In summary, there are several changes in the epithelial barrier of patients with AR that can contribute to the amplification and maintenance of the nasal inflammation cycle.^20^, ^76^

#### Nitric oxide

In the era of precision medicine, certain molecules are identified as biomarkers in the pathogenesis of AR, providing information for the identification of endotypes and guiding precise treatment interventions. Nitric oxide, detected by a non-invasive method in exhaled air and by the exhaled fraction of NO (FeNO), has been considered a biomarker of type 2,^80^ inflammation with high concentrations in eosinophilic inflammation in the airway.^81^ In addition to being a good diagnostic tool, the determination of exhaled NO generates information about the phenotype/endotype of the disease and the response to treatments with steroids or biological drugs.^82^

The basal production of tiny amounts of NO (phentomolar to picomolar), produced by the bronchial epithelium, is fundamental for respiratory physiology.^83^ NO regulates the tone of the bronchial muscles, blood flow, stimulates lung development, promotes ciliary motility and stimulates surfactant production. NO also has antimicrobial activity against bacteria and viruses.^84^ In the upper airway, NO acts as a vasodilator and relaxes smooth muscles, stimulates mucus secretion and regulates the frequency of ciliary beating.^85^

In the respiratory tract, NO is synthesized from L-arginine by NO synthase (NOS), which has three isoforms: neuronal NOS (nNOS), inducible NOS (iNOS) and endothelial NOS (eNOS).^86^ iNOS, induced by cytokines and/or endotoxins, is independent on calcium and can produce NO at nanomolar levels, for prolonged periods and contributes to the pathophysiological effects of NO, detectable in exhaled air.^87^

In the paranasal sinuses, iNOS acts constitutively, producing large amounts of NO under the stimulus of pro-inflammatory cytokines (e.g., TNF and IL-1), possibly stimulated by biofilms.^88^ However, during Th2 inflammation in the upper airway, the primary sources of NO are epithelial cells and macrophages.^89^ The expression of iNOS in the allergic response is observed under the stimulation of several Th2-type cytokines, such as IL-4 and IL-13, acting through the STAT pathway.^6^, ^90^, ^91^

Prominent levels of NO participate in airway inflammation, the production of free radicals, bronchial hyperreactivity, mucus hypersecretion, increased vascular permeability, reduced ciliary beat and tissue damage.^80^

There are two different methods for measuring nasal NO concentration: nasal FeNO, obtained by nasal exhalation, and a second when the measurement is obtained by serial transnasal flows, called nNO.^91^ nNO oxide measurements are non-invasive procedures that can be easily applied in the pediatric population. The preferred method is chemiluminescence, which can be applied to children over four years of age and adults who can collaborate by maintaining palate closure.^92^ This method uses air exhaled through the nose against resistance, using apnea, while the electrochemical analysis employs tidal breathing. Between the two methods, electrochemical analysis is less studied and has a lower degree of reproducibility.^93^

The upper airway comprise a complex system of communicating cavities (nasal cavity, paranasal sinuses, middle ear and nasopharynx) and each of these areas can contribute to measured NO.^94^ The primary origin of intrinsic NO production in the airway is the paranasal cavities.^95^ Nasal mucosa edema can lead to occlusion of the sinus ostium, without distribution of NO to the nasal cavity, which may explain the absence of exhaled NO in severe cases of ostium obstruction.^96^ Therefore, the severity of inflammation of the nasal mucosa does not correlate with detectable levels of nNO. On the other hand, nNO levels tend to decrease after treatment.^97^ It is important to emphasize that nNO is not a determination included in routine practice due to heterogeneous results and a lack of consensus regarding the best technique to be used. An exception can be made in cases of primary ciliary dyskinesia, where nNO levels are 90%–98% lower than in control patients.^95^ In cystic fibrosis and primary ciliary dyskinesia, there is a reduction in NO synthesis with consequent changes in mucociliary clearance.^98^

In general, interpretation of nNO levels should be done in conjunction with clinical findings and other diagnostic tests.

#### Microbiome

Microbiome refers to the set of genetic material of microorganisms that occupy a given system. Changes in the microbiome of human beings have been implicated in the pathophysiology of several immune-mediated conditions^99^ and is considered one of the factors involved in the epithelial barrier theory.^100^ This theory postulates that immune-mediated diseases result from persistent epithelial inflammation (epithelitis) caused by various substances that damage the epithelial barrier, and genetic susceptibility.^101^ A damaged barrier promotes changes and loss of biodiversity that generate instability and dysfunction of the microbiome, with a predominance of one or a few microorganisms, what we call dysbiosis. This, in turn, can impair homeostasis, promoting immune systemdysregulation.^100^, ^101^, ^102^ The complex interaction between the human microbiome and the innate and adaptive immune system is beyond the scope of this text.

The relationship between the microbiome and AR can be approached from two main aspects: environmental exposure and the composition of epithelial barriers.

Since the publication of the hygiene hypothesis in 1989,^103^ much evidence has accumulated that exposure to microbial diversity exerts a protective effect on the development of respiratory allergy.^20^, ^104^ The interrelationship between microbial diversity/composition and the immune system, and between different organs, pointing out the role of dysbiosis in promoting a pro-inflammatory environment, as well as the interference of inflammation in the microbial constitution, has been extensively explored in recent decades.^105^

The environmental microbiome includes the microorganisms and their metabolites that we come into contact within the environment. During the first year of life, the interaction between the immune system and the environmental microbiome influences the human microbiome composition and directs the immune response towards tolerance. Over time, the environmental microbiome becomes a source of immunological stimulation and infection.^106^

In 2003, it was identified that children in Finnish Karelia had much more allergy symptoms and much higher levels of IgE than those in Russian Karelia. Karelia is a region in northern Europe divided between Finland and Russia, which have the same climatic conditions, similar ancestry, but a great socioeconomic contrast, with the Russian side being rural. Ten years later, a new study with these populations identified continued disparity in the prevalence of allergic diseases, as well as significant differences in the skin and nasal microbiome between them, with greater microbial diversity, with the Acinetobacter genus being more abundant and diverse, in the population on Russian side. What the authors suggest is that early exposure to the environmental microbiome may be biologically related to the development of allergic manifestations at an early age.^107^

However, it became clear over the years that microbial exposure alone does not define the development of allergy or not, which is not surprising in the case of a multifactorial clinical condition. Results obtained by Sun and collaborators in the analysis of household dust suggest that metabolites and intra-household chemicals are better indicators of respiratory allergy than the intra-household microbiome.^108^

Regarding the composition of the microbiome of epithelial barriers, many studies have explored the diversity of the composition of the intestinal microbiome between healthy people and those with different manifestations of respiratory allergy.

There are factors considered crucial to the development of the intestinal microbiome: type of birth; type of breastfeeding; gestational age; maternal vaginal, skin and intestinal microbiota; maternal lifestyle (particularly type of diet); use of antibiotics; environmental exposure to pollutants, animals and others.^109^ Food components are capable of interfering with the immune response and the composition of the intestinal microbiome. A significant risk of allergic diseases arises from a diet rich in calories, proteins and animal fat, saturated and medium-chain fatty acids, simple sugar and processed foods, and low in fiber, iron, zinc and vitamins A, D and E.^110^

The term “gut-organ axis” points to the complex relationship between the intestinal microbiome and the development and functioning of the immune system in the skin, respiratory tract and central nervous system.^109^

Reduction in the diversity of microorganisms and reduction in the presence of bacteria of the Firmicutes genus (producers of butyrate, a recognized regulator of the epithelial barrier integrity with immune system actions, such as promoting macrophage differentiation and development of regulatory T-cells) have been reported in several publications involving allergic diseases in children and adults.^109^, ^111^ Lower gut microbiome diversity in infants has been associated with a higher risk of allergic sensitization, allergic rhinitis and peripheral eosinophilia at school age.^112^

In adults with AR, reduced diversity α was demonstrated in the intestinal microbiome, characterized by an increase in the phylum Bacteroidetes and the genera Escherichia-Shigella, Prevotella and Parabacteroides,^113^, ^114^ as well as a reduction in Firmicutes, Oxalobacter and Clostridioides.^114^

A study of the intestinal microbiome in individuals with allergic rhinitis revealed that bacteria from the Coriobacteriaceae family had a protective effect, while those from the Victivallaceae family were identified as a risk factor for allergic rhinitis.^115^

Further, research sought to identify differences in the microbial composition of the nasal mucosa between healthy individuals, with rhinitis, chronic rhinosinusitis without or with polyposis, with or without asthma.^105^ It is important to highlight that the material collection site, from the anterior nasal cavity, middle or lower meatus may interfere with the profile of microorganisms identified in different studies. No significant differences were found regarding microbial diversity between AR patients and healthy controls, but some differences were found regarding their composition.^116^, ^117^

Under normal conditions, the nasopharyngeal microbiome is composed of Proteobacteria (*Moraxella* spp. and *Hemophilus* spp.), Firmicutes (*Staphylococcus* and *Dolosigranulum* spp.), and Actinobacteria (*Corynebacterium* spp.).^116^, ^118^ Propionibacterium acnes has been identified as a bacterium important in healthy nasal mucosa.^119^

Individuals with AR more frequently presented bacteria of the Spirochaetae genus in the nasal mucosa, as well as greater amounts of Pseudomonas and Peptostreptococcaceae and lower amounts of Lactobacillus, according to Gan and collaborators.^120^ On the other hand, Yuan and collaborators identified a greater abundance of Actinobacteria in people with AR a higher amount of Actinobacteria, Staphylococcus, Prevotella and Klebsiella, and less Pelomonas, compared to healthy controls.^116^ No significant differences were identified in nasal microbial composition in patients with rhinitis in relation to patients with allergic rhinoconjunctivitis.^117^

Staphylococcus, particularly S. aureus, colonizes individuals with AR more frequently than controls (44% vs. 20%) and is associated with higher clinical scores in patients with rhinitis.^116^, ^121^

Persistent inflammation and infection characterize chronic rhinosinusitis, with the presence of S. aureus being particularly important.^105^ It has been reported that no type of bacteria appears to be specifically related to chronic rhinosinusitis, but rather changes in its composition with functional changes in the microbial community.^122^ However, Huntley and colleagues found that Citrobacter was found only in patients with chronic rhinosinusitis with polyposis and not in healthy controls or patients with rhinosinusitis without polyposis. S. aureus has been found more frequently in individuals with chronic rhinosinusitis with polyposis and eosinophilia.^123^

There were no differences in microbial diversity between patients with allergic rhinitis and non-polyposis rhinosinusitis, but rather differences in composition, with greater abundance of Pseudomonas and Peptostreptococcaceae, and lower abundance of Lactobacillus in allergic rhinitis, and greater abundance of Hemophilus and lower abundance of Moraxella in chronic non-polyposis rhinosinusitis.^120^

A study analyzing the nasal microbiome of healthy controls, patients with rhinitis, rhinosinusitis with and without polyposis showed lower diversity α in patients with chronic rhinosinusitis without polyposis than in the other groups, in addition to a higher abundance of anaerobic bacteria.^124^

Involuntary exposure to cigarette smoke has been identified as a factor capable of promoting nasal dysbiosis.^125^

Viral respiratory infections caused by influenza A, rhinovirus A and C, adenovirus and respiratory syncytial virus promote changes in the nasal microbiome such as the proliferation of S. pneumoniae. On the other hand, the predominance of some bacteria in the nasal microbiome increases susceptibility to some viruses, such as the predominance of hemophiles in relation to rhinovirus A and *Moraxella* sp. in relation to rhinovirus C.^126^

Dysbiosis in the mucosa of the inferior nasal turbinate, with an increase in S. aureus and a decrease in Propionibacterium acnes, was related to higher IgE values in adult individuals with AR.^127^

In individuals with rhinitis and wheezing, an increase in Proteobacteria and Firmicutes was identified, with a reduction in Corynebacteriaceae.^128^

Patients with AR or asthma, with or without AR, showed reduced diversity α (number of taxonomic groups and relative abundance of each group) of the nasal bacterial microbiome compared to healthy controls, but with a greater influence on diversity in patients with asthma, with or without rhinitis. In relation to phyla and genera of bacteria (diversity β or measure of similarity between two communities of microorganisms), the composition was similar in healthy controls, people with rhinitis and people with asthma without and with rhinitis, being more prevalent, in descending order, Firmicutes, Bacteroidetes, Proteobacteria and Actinobacteria. Firmicutes was more abundant in patients with allergic rhinitis and less abundant in patients with asthma (with and without rhinitis). More relevant differences in relation to gender and species were detected between healthy controls and patients with respiratory allergy and less relevant differences between patients with rhinitis and asthma (with or without rhinitis). No significant differences were identified in the taxonomic distribution of patients with asthma without and with rhinitis. Similarly, no significant differences were identified in α diversity between patients with controlled, partially or uncontrolled asthma. However, diversity β clearly separates two groups: uncontrolled asthma and controlled/partially controlled asthma (both with or without rhinitis). Patients with uncontrolled asthma had a higher proportion of Cyanobacteria and a lower proportion of Firmicutes.^129^

Another relevant fact is that the nasal microbiome composition in children with AR differs from that of adult patients. The microbiome composition changes over time and the early-life profile is related to stability and patterns of change throughout life.^129^ The diversity of the nasal microbiome increases over time in normal individuals, while it tends to reduce in children with rhinitis, with or without wheezing.^128^

Studies on the nasal microbiome in the pediatric population are few and reveal that Acinetobacter, Corynebacterium, Dolosigranulum, Hemophilus, Moraxella, Staphylococcus and Streptococcus genera are dominant in any pediatric age group. Acinetobacter and Pseudomonas are more abundant in the nasal cavity, while Streptococcus and Moraxella were predominant in the hypopharynx of children with allergic rhinitis. Abundance of *Staphylococcus* spp. has been described in the anterior nasal cavity and hypopharynx of children and adolescents with allergic rhinitis, cigarette exposure and rhinoconjunctivitis.^130^

Studies that correlated epithelial microbial composition and metabolomic analysis in blood in search of biomarkers for AR demonstrated that there is a correlation between the microbiome and metabolism in animal^129^ and human models.^116^ Ma and collaborators identified the porphyrin, arachidonic acid and purine pathways as the most commonly altered in AR.^131^ On the other hand, according to Yuan and collaborators, the most frequently altered metabolic pathways in people with AR were the linoleic acid, arachidonic acid and caffeine pathways.^116^ Therefore, there is still no consensus recommendation for biomarkers that can be applied in clinical practice.

Despite the growing number of publications on the topic, there are still relevant divergences between studies on the relationship between microbiome diversity/composition and allergic diseases, particularly AR. Much remains to be known. There are conflicting results, poor data on the pediatric population, on the composition of viruses and fungi, or the interference of treatment with medications or allergen-specific immunotherapy.

#### Joint airway

Since the beginning of the 20th century, it has been recognized that allergic rhinitis, asthma and rhinosinusitis often occur as comorbidities. From the end of the 20th century and the beginning of the 21st century, evidence accumulated that these diseases were manifestations related to an inflammatory process occurring in a continuous airway, giving rise to the integrated airway hypothesis, or joint airway disease, chronic inflammatory respiratory syndrome or rhinosinobronchitis.^132^, ^133^

The concept of a joint or continuous airway encompasses the nose, middle ear, paranasal sinuses, bronchi and lungs.^134^

There is epidemiological, pathophysiological and clinical evidence that inflammation is a central process in this concept of Joint Airway (JA), which are related from an anatomical, histological and immunological point of view, functioning as a morphological and functional unit.^33^, ^135^, ^136^

Between 20% and 40% of patients diagnosed with AR have asthma, on the other hand, more than 80% of individuals diagnosed with asthma have symptoms of AR.^132^, ^133^, ^136^, ^137^, ^138^ Chronic rhinosinusitis with polyposis is associated with asthma, particularly in individuals with respiratory disease exacerbated by acetyl salicylic acid.^139^ The prevalence of asthma in patients with non-allergic eosinophilic rhinitis, respiratory disease exacerbated by aspirin, allergic fungal rhinosinusitis, chronic rhinosinusitis with polyps and atopic disease of the central compartment is described, respectively in 23.8%; 100%; 19%–73%; 20%–60% and 9.8%–17.1% of cases.^140^

Pediatric chronic rhinosinusitis is related to adenoid disease and asthma, as well as cystic fibrosis and primary ciliary dyskinesia.^141^

Furthermore, AR and non-allergic rhinitis are risk factors for developing asthma or persistence of asthma.^142^

The epithelial lining of the Upper Airway (UA) and Lower Airway (LA) have characteristics in common (pseudostratified ciliated epithelium, basement membrane, lamina propria, goblet cells) and some differences, such as the rich vascularization in the upper airway and the presence of musculature smooth in the lower airway.^136^

JA presents an allergic and a non-allergic phenotype. The majority of children and approximately 50% of adults present the allergic phenotype, with T2-type inflammation, produced from changes in the epithelial barrier, innate lymphoid cells and specific IgE for aeroallergens. The pathophysiology involved in the non-allergic phenotype is less known.^20^, ^136^

The mechanisms proposed to explain the interaction between UAW and LAW diseases are changes in ventilation patterns and the action of distant inflammatory mediators. Nose dysfunction causes oral breathing and poor air conditioning that reaches the airway, increasing the risk of asthma.^136^, ^143^ Systemic absorption of inflammatory mediators produced in the upper airway and lower airway promotes a distant inflammatory response at other points in the respiratory tract. There is no satisfactory evidence on aspiration of nasal contents and nasobronchial reflex mechanisms, despite the recognized stimulation of nasopharyngeal receptors as a cause of cough.^20^, ^143^

In relation to clinical evidence, it has been demonstrated that the treatment of AR produces a reduction in symptoms, exacerbations and hospitalizations due to asthma.^134^, ^144^, ^145^ Just as there is evidence that the treatment of non-allergic eosinophilic rhinitis, respiratory disease exacerbated by aspirin, allergic fungal rhinosinusitis, rhinosinusitis chronic disease with polyps and atopic disease of the central compartment also promotes clinical and objective improvement (spirometry) of asthma.^140^

Studies on the pathophysiology of JA inflammation are still needed and could lead to the discovery of biomarkers and new treatments. Leukotriene E4 is a promising biomarker. The relationship between JA diseases and the microbiome and its changes is an uncharted field.^146^

## Allergic rhinitis

### Epidemiology of allergic rhinitis in Brazil

Population studies are essential for understanding the extent of a disease in a given population. However, in Brazil they are scarce. The International Study on Asthma and Allergies in Childhood (ISAAC), started in the 1990s, enabled initial data to be obtained on the prevalence of rhinitis among children and adolescents in various parts of the world, as well as evaluating whether or not it has increased with time.^147^, ^148^

In the 2010s, ISAAC was discontinued and replaced in 2012 by the Global Asthma Network (GAN), which uses a protocol and assessment instrument similar to those of ISAAC, which has enabled comparisons between the two studies, in addition to having a version for adults.^149^ Another important point to comment concerns the way patients are studied: symptoms, previous diagnosis by a doctor, physical examination, among others.

#### In adults

Epidemiological studies in adults are scarce and most often limited to small population groups. In 2010, Allergies in Latin America (AILA) assessed in an unprecedented way the prevalence and burden of allergic rhinitis symptoms among individuals over 4 years of age, living in eight Latin American countries, by telephone interview. The average prevalence of allergic rhinitis was 7%, being higher among adults.^150^

The GAN study was carried out in 17 countries (Europe, Latin America) and the prevalence of hay fever was 14.4% among parents of children and adolescents who participated in the study.^151^ In Brazil, the same study was carried out in a single center in the city of Uruguaiana, Rio Grande do Sul, and the prevalence of allergic rhinitis observed was 31.7% among parents of adolescents, slightly higher than that observed among their children (28.0%)^152^ and much higher than that obtained in the previous study.

The use of different definitions to identify “cases” (clinical history, medical diagnosis, medication consumption, among others) may be one of the reasons for the observed differences. A study carried out on a representative sample of adults living in the city of São Paulo revealed that the prevalence of allergic rhinitis (clinical history and allergic confirmation) was 25.2%.^153^

#### In children and adolescents

After ISAAC was established and more robust data was obtained, many researchers used it to determine the prevalence of asthma and allergic diseases in various locations across the country. Initial data in Brazil indicated that the prevalence of allergic rhinitis was 12.8% and 18.0% for children (6–7 years) and adolescents (13–14 years), respectively.^154^

In subsequent reevaluations, especially among adolescents and in the same locations, variations in the observed rates were documented with a general upward trend in most cities, with the average prevalence of rhinoconjunctivitis remaining at 16.2%.^155^, ^156^ In Brasília, the ISAAC study carried out 6 years apart showed a variation in the prevalence of allergic rhinitis from 12.2% to 20%.^157^ In Santo Ângelo, in the interior of Rio Grande do Sul, during a seven-year follow-up period there was a reduction in rhinoconjunctivitis rates from 48.9% to 38.8% in the general population and with higher rates among adults.^158^

Studies in infants are scarce. In the first years of life, diagnosing allergic rhinitis is not an easy task since viral infections of the upper airway, most often characterized by sneezing, runny nose and nasal obstruction, are common. Adding questions to the International Infant Wheezing Study (EISL) questionnaire, Chong Neto and collaborators documented, among infants in their first year of life, the prevalence of nasal symptoms (an episode of sneezing, runny nose or nasal obstruction) without having a cold 48, 3% among the infants they assessed.^159^ On the other hand, in a birth cohort, the prevalence rates of chronic rhinitis and rhinoconjunctivitis among children assessed at six years of age were 36.9% and 23.5%,^160^ respectively, and similar to the data obtained by ISAAC in Brazil.

### Geographic variations and climate effects on AR prevalence

The impacts of climate change on allergens and allergic diseases are complex, and the multifactorial health effects of air pollutants depend on a wide range of exogenous and endogenous factors, including the physical and chemical characteristics of the pollutants and the anatomical or physiological condition of individuals, such as breathing patterns or activity level.^161^, ^162^

Components of air pollution can interact with airborne allergens and increase the risk of atopic sensitization, duration of the pollen season and exacerbation of symptoms in sensitized individuals, as has been observed in relation to pollens.^161^, ^162^, ^163^, ^164^

Global or regional changes in temperature, humidity, air pollution, or other environmental conditions can modify the growth, survival, and allergen production of House Dust Mites (HDM) and natural molds.^164^ Consequently, sensitization to HDM and molds has increased in some regions of the world, especially in subtropical and tropical areas.^165^, ^166^ The synergistic effects between extreme heat and aeroallergens intensify the toxic effect of air pollutants, which in turn increases the allergenicity of aeroallergens.^166^

According to international data from ISAAC, the prevalence of allergic rhinitis was more pronounced in centers located close to the Equator.^148^, ^167^

## Allergic rhinitis ‒ risk and protective factors

### Genetics

The similarity between the phenotypes of atopic diseases indicates that biological and etiological factors overlap between these conditions. Common genetic risk variants affecting many atopic diseases demonstrate that they share common pathogenic characteristics, providing information for the development of new treatments.^168^

As with other allergic diseases, studies with twins provide evidence of genetic inheritance in AR. The concordance rate for identical twins is 45%–60%, higher than that for fraternal twins, where it is no more than 25%. The calculated inheritance for AR is estimated between 33% and 91%. Many genes are involved in various diseases related to the immune system, including allergic and autoimmune disorders.^168^

The BCAP gene on chromosome 10q24.1 and the MRPL4 gene on chromosome 19p13.2 have been associated with AR and atopy in a genome-wide association study.^169^ Linkages within known regions, such as HLA-DQ and NPSR1 loci, have also been replicated. Single Nucleotide Polymorphisms (SNPs) in the TNF-α gene are also known as a high-risk factor for RA.^170^, ^171^

### Gene-environment interactions and epigenetic effects

There is evidence that changes in genetic functions without changes in DNA sequences are important in the pathophysiology of chronic diseases, including allergies. Many studies have elucidated the role of epigenetics in the pathogenesis of AR, showing that many modifications in DNA methylation and histone acetylation can occur in response to allergens.^172^, ^173^ In AR, it has recently been observed that the severity of allergic responses can be predicted by the level of DNA methylation in the SLFN12 gene when exposed to grass pollen. It has been shown that histone modification and miRNA level changes are different in the candidate gene in AR patients.^174^

Studies performed on AR patients and immune cells isolated from AR patients have shown that Histone Deacetylase (HDAC) is increased in immune cells and that inhibiting HDAC can help improve AR. A study of AR patients showed that HDAC1 is upregulated in nasal epithelial cells compared to healthy controls.^175^ IL-4 can increase HDAC1 expression, producing nasal epithelial barrier dysfunction. HDAC1 inhibitors, such as trichostatin A and sodium butyrate, can inhibit nasal epithelial dysfunction in mice.^176^ Many studies have reported that the expression of TWIK-Related Potassium Channel 1 (TREK-1) is downregulated in AR patients. TREK1 expression is upregulated and HDAC1 is downregulated in the nasal mucosa by allergen-specific immunotherapy.^175^ Thus, increased HDAC expression in nasal epithelial cells may reduce TREK1 expression, producing inhibitory effects in AR. Inhibition of HDAC1 promotes IL-10 and Foxp3, and blocks excessive activation of immune cells. HDAC inhibitors can decrease TNF-α expression. These results indicate that an increase in HDAC activity may contribute to the pathogenesis of AR by increasing pro-inflammatory cytokines and decreasing anti-inflammatory cytokines.^177^

Another study also demonstrated the correlation between changes in miRNA expression, particularly with the decrease in the expression of miR-21 and miR-126 in neonatal mononuclear leukocytes, and the development of AR.^178^, ^179^ In childhood AR, miR-181a levels may be a predictor of disease severity.^180^ MiRNA alterations in AR patients include upregulation of miR-498, miR-187, miR-874, miR-143, and miR-886 -3p and downregulation of miR-18a, miR-126, let-7e, miR -155 and miR-224.^180^ Others have also shown that miR-221 and miR-142-3p highly expressed in the nasal mucosa may be biomarkers for AR, promoting and reinforcing mast cell degranulation, respectively.^181^, ^182^

### Risk factors

#### Inhalant allergens – intrauterine and early childhood exposure

Although the predisposition to AR is genetic, environmental influences play an important role in its onset and persistence. Given that genetic factors cannot be modified, a detailed assessment of environmental influences is necessary to identify the most relevant modifiable risk factors. It is not possible to explain the increase in the incidence and prevalence of allergies over several decades solely by changes in the genetic basis, which makes clear the contribution of environmental modifying factors, which have gained even more support in epidemiological observations.^182^, ^183^

Environmental determinants, such as exposure to allergens, air pollution, climate change, ozone, smoking, viral infections and environmental toxic substances, may be responsible for much of the increase in the prevalence of AR. Furthermore, specific epigenetic changes caused by environmental exposure can contribute to cellular homeostasis and the development of allergic diseases.^20^ Many researchers direct their studies to better understand the role of exposure to aeroallergens as a risk factor for the development of AR.

Allergic sensitization develops during childhood as a result of the interaction between genetic determinants and environmental exposures, which can be either inhibitory or promotive. Environmental exposure to allergens is believed to play a key role in the development of sensitization, but the level of exposure is not necessarily related to the risk of sensitization. It is possible that certain adjuvant effects are necessary for mild exposure to promote sensitization.

Previous studies have shown ambiguous results that may reflect the importance of the timing and amount of allergen exposure during childhood or the need for exposome-related adjuvant effects.^184^, ^185^

The first few years of life have been identified as a critical period for intra- and extra-home environmental exposure associated with childhood allergies.^186^ Although increasing evidence demonstrates a significant role for early postnatal exposure to indoor environments in the development of respiratory allergy, little information has been obtained from preconception or prenatal exposure. Limited available evidence has explored associations between indoor tobacco smoke, modern furniture and decorations, mold or damp, and pets (dogs) in pregnancy and the development of childhood rhinitis.^187^

Retrospective cohort study involving 8,689 preschool-aged children conducted in China during 2019–2020 analyzed household environmental exposures during one year before conception, during pregnancy, in the first year of life, and in the year before the study. The authors concluded that exposure to indoor pollution represented by cigarette smoke and renovations, as well as allergens associated with humidity, furry pets and pollen during all time windows evaluated, especially before birth, were significantly associated with a risk increased AR in children.^187^

#### Mites

Most studies have failed to demonstrate an association between early exposure to dust mites and the development of AR. There are still discrepant data, in which studies show that early exposure to house dust mites is a protective factor for AR,^188^ while others propose exposure to dust mites as a risk factor for AR.^189^ Studies on early exposure to dust mites and the development of AR are conflicting and additional studies are needed.

#### Pollens

Exposure to pollen is an environmental factor that is known to influence allergic diseases. Study on Seasonal AR (SAR) combined the use of DNA methylation and gene expression arrays. Methylation patterns observed in isolated and in vitro-cultured CD4+ T-cells (but not in mRNA expression profiles) made it possible to clearly distinguish between samples obtained from SAR patients and controls.^190^ An additional interesting feature of the study was that the samples were collected from each participant inside and outside the pollen season and therefore also the environmental influence on DNA methylation was directly investigated. Furthermore, methylation profiles were found to be significantly associated with disease severity during the pollen season.^190^

#### Animal fur

There is inconsistency in the data related to the maintenance of furry pets in the home environment during the postnatal period in AR in children. Dogs, e.g., have been previously identified in the literature as both a risk factor^191^ and a protective factor.^192^ In a single-center prospective cohort in the greater Copenhagen region, Denmark, the authors followed^411^ children and found no association between exposure to dogs or cats during the third trimester of pregnancy or the first year of life and the development of allergic sensitization to dogs or cats during childhood. Likewise, they did not observe an association between the levels of dog, cat or house dust mite allergens in bed dust samples collected in one year and allergic sensitization to these allergens during childhood.^185^

#### Fungi

Exposure to indoor mold and humidity is associated with AR; and it is an environmental risk factor for it. Studies have found that self-reported problems with damp or mold in buildings where people live or work are associated with respiratory and/or allergic illnesses.^193^

A Swedish study showed that exposure to moisture/mold during childhood increased the risk of asthma and rhinitis up to 16 years of age, particularly for diseases without IgE sensitization. Early exposure to mold or moisture may be particularly associated with persistent asthma into adolescence.^194^ Another previous study indicated a stronger effect of exposure to mold/moisture variables during early life compared to current exposure on later development of AR in children.^195^

#### Environmental pollution

Epidemiological studies demonstrate a positive association between air pollution and AR. The mechanisms by which air pollution affects rhinitis are dependent on the type of pollutant, the rhinitis phenotype studied and, in particular, allergy sensitization.^196^

Systematic review of studies carried out in Latin America confirmed that the chance of a person exposed to air pollutants having AR is 43% higher than that of a person not exposed.^197^

Geographic area and socioeconomic status were identified as potential modifiers of this association, with the effects of air pollutants being significant in developing countries.^198^

Prolonged exposure to Particulate Matter (PM) with a diameter of up to 10 μm (PM_10_) and PM_2,5_ (up to 2.5 μm in diameter) is associated with an increase in the prevalence of AR by 14%–25% and 6%–9% for Nitrogen dioxide (NO_2_).^199^ A 10 μg/m^3^ increase in concentrations of PM_2.5_, PM_10_, NO_2_, and Sulfur dioxide (SO_2_) has been associated with increases in outpatient care for patients with AR of 1, 24%, 0.79%, 3.05% and 5.01%, respectively. Stronger associations were observed among men than women, as well as in young adults (18–44 years of age).^200^

Short-term exposures to PM_2.5_, PM_10_, NO_2_, SO_2_ ozone (O_3_) and Carbon Monoxide (CO) were also significantly associated with an increased risk of outpatient visits due to AR.^201^ Other authors also observed similar results.^202^

There is an important relationship between environmental air pollution and forest fires with the development of Chronic Rhinosinusitis (CRS) in healthy individuals, as well as an increase in the severity of symptoms in patients with CRS.^203^

Review article demonstrated that early exposure to air pollution in the prenatal period and in early childhood may be associated with the development of long-term AR and that chronic exposure to air pollution is associated with the risk of increased exacerbations and urgent hospital visits.^204^

Lu et al. evaluated the effects of intrauterine and early postnatal exposure to outdoor air pollution in children with Medically Diagnosed AR (MDAR). The authors demonstrated that MDAR was associated with intrauterine exposure to CO, at different moments of exposure. Early postnatal exposure to PM_2.5_, PM_10_, and in the first year of life has also been associated with the development of MDAR.^205^

Environmental, domestic and occupational irritants and pollutants also stimulate the nasal mucosa to release inflammatory mediators that increase nasal hyperreactivity that overlaps with rhinitis symptoms.^206^

Internal pollution caused by the burning of cooking fuel (biomass, wood, liquefied petroleum gas), tobacco smoke, exposure to air pollutants from traffic and the burning of fossil fuels, as well as bioparticles, such as aeroallergens, are factors known to cause respiratory diseases. Other relevant components are chemical air pollutants, such as gases, particulate matter, formaldehyde and Volatile Organic Compounds (VOCs).^206^, ^207^

Prenatal exposure to indoor pollution, emitted by contemporary furniture or redecoration, as well as moisture-related allergens for a period of one year before conception and pregnancy was significantly associated with increased AR.^187^

Indoor environments in homes, schools, daycare centers and social recreation environments can have a high concentration of particulate matter from motor vehicle roads.^208^

#### Tobacco smoke

Exposure to cigarette smoke increases allergic airway inflammation. However, some studies have shown a controversial association between this pathological finding and current exposure to cigarette smoke.^20^

Observational studies on smoking and risk of hay fever and asthma have shown inconsistent results, as they may be influenced by confounding and reverse causality. Skaaby and colleagues suggest that smoking may be causally related to a higher risk of asthma and a lower risk of hay fever.^209^

It is known that maternal exposure to tobacco during pregnancy poses a potential health risk to children. Zhou and colleagues demonstrated that maternal exposure to smoking during pregnancy can increase the risk of AR in the offspring.^210^

Pooled analysis of five European birth cohorts found that children with high early exposure were more likely than unexposed children to have transient asthma and persistent rhinoconjunctivitis.^194^

Although pediatric data show a trend towards a higher prevalence of rhinitis in association with higher levels of exposure to secondhand smoke, adult data are less consistent. The exact biological mechanism for these associations is multifactorial but does not appear to be driven by an IgE-mediated allergic reaction.^211^, ^212^

The effects of new tobacco products such as Electronic Cigarettes (EC) and Heated Tobacco Products (HTP) on AR and asthma are not well known. The use of EC and/or HTP increased the risk of AR and asthma in Korean adolescents.^213^

Among Swiss adolescents who combined EC and hookah use with conventional cigarettes, current respiratory symptoms such as rhinitis, dyspnea, and wheezing were more common among frequent smokers (44%, 30%, 12%, respectively) than among those who never smoked.^214^

A cohort study of patients treated at an otorhinolaryngology clinic with known use of EC found that chronic otitis media (17.4%) and AR (13.0%) were the most common inflammatory diagnoses.^215^

### Socioeconomic factors

The role of socioeconomic factors in the development of AR has complex underpinnings. They include housing conditions, air quality, water supply, education and access to healthcare.^20^

Recent studies have proposed that excessive handwashing may lead to the development of allergic diseases by reducing the integrity of the skin's epithelial barrier. Wee and colleagues demonstrated that among Korean adolescents, having a high socioeconomic status, better father and mother education were associated with a high hygiene score, which was associated with the presence of AR.^216^

A cross-sectional study analyzed the association between socioeconomic level and hay fever in adolescents and observed that parental education level was a socioeconomic factor associated with an increased risk of hay fever, but not family income.^217^

Penaranda and collaborators in a study in Colombia, found that the factors associated with AR in children/adolescents were a family history of paracetamol consumption and high socioeconomic level.^218^ Lee and collaborators identified as risk factors among Korean adolescents, having a good socioeconomic level and high academic performance.^219^

A study on the prevalence of AR in Brazilian adolescents, as part of the Global Asthma Network (GAN), demonstrated that lower education among parents/guardians was a factor negatively associated with the occurrence of AR.^152^

Although available evidence indicates that higher socioeconomic status is associated with an increased risk of AR, the data are conflicting.

Not all studies demonstrated a positive relationship between AR and higher socioeconomic status. A cross-sectional study carried out in Turkey found that poor living conditions and income were associated with a higher risk of AR.^220^

A cohort study carried out by Grabenhenrich and colleagues in Germany found no relationship between socioeconomic status, lifestyle and the development of AR.^221^

On the other hand, Barreto and collaborators found that a higher educational level, better socioeconomic level, enable patients to have better knowledge and better understanding of the symptoms and the disease, better opportunities for diagnosis and access to adequate medical treatment.^222^

Tan and collaborators found a sad reality, especially among the poorest populations, as 70% of patients who purchased nasal treatment at a community pharmacy self-administered their AR with medications sold without a prescription. Of all the patients they analyzed with AR symptoms, only 44.3% had a medical diagnosis, due to the difficulty in accessing healthcare.^223^

### Protective factors

#### Breastfeeding

Although exclusive Breastfeeding (BF) is known to bring numerous benefits to the child's health, there is still no consensus that it is capable of preventing the development of AR.^224^

Some prospective cohort studies carried out between 2016 and 2022 demonstrated that longer BF (over 6 or 12 months) was associated with a lower risk of developing AR.^225^, ^226^, ^227^ However, a population-based cohort study, on the contrary, pointed out to an increased risk of hay fever associated with MA,^228^ while another study demonstrated only protection for recurrent rhinitis.^152^

A 2022 systematic review concluded that breastfeeding for at least six months is capable of protecting against the development of allergic rhinitis up to 18 years of age.^229^

The mechanisms involved are not exactly known, but it is known that breast milk has a series of elements that stimulate the immune system and have a beneficial effect on the intestinal microbiota of infants.^230^, ^231^, ^232^

It is necessary to consider that the studies carried out and those included in the systematic reviews are observational and it is quite possible that, due to the difficulty in distinguishing viral infections of the upper airway from allergic rhinitis in young children, the prevention of infections promoted by breastfeeding has been confused with rhinitis prevention.^20^

What can be concluded so far is that exclusive breastfeeding should be encouraged due to its numerous beneficial effects for breastfeeding women and infants and that it possibly has a protective effect on the development of AR.^20^

#### Exposure to animals in childhood

The association between exposure to animals, allergic sensitization and the presence of allergic diseases, such as rhinitis, is complex and not yet fully understood. There is solid evidence that sensitization to domestic animals, particularly dogs and cats, is a risk factor for the development of allergic rhinitis and asthma, and exposure to domestic animals is recognized as an important trigger for symptoms and exacerbations in sensitized patients.^233^ Cross-sectional studies have found a positive association between exposure to domestic animal allergens and the presence of allergic sensitization and/or rhinitis in adults, but not in children.^234^ The age of exposure to domestic animals appears to be decisive in the direction of its association with development of allergic diseases. Despite some conflicting results, several studies have documented that early exposure to domestic animals, in the first years of life and before the appearance of symptoms, can help prevent the development of allergic diseases.^235^, ^236^ The mechanisms involved in this prevention are not completely known, with evidence of modification of the Th2 response with high rates of exposure to allergens and modification of the Th1/Th2 balance by microbiological products, such as endotoxins, in line with the hygiene theory.^233^

#### Biodiversity/Barrier theory/Hygiene hypothesis

In 1989, David Strachan presented the “hygiene hypothesis” to try and explain the increase in allergic diseases that had been occurring and hypothesized that fewer infections would cause a shift towards allergic responses. Recurrent microbial exposure would initiate a Th1 response rather than a Th2-mediated immune response associated with elevated levels of interleukin IL-4 and IL-5 and eosinophilia.^102^

Later came the “biodiversity theory” which postulates that reduced environmental biodiversity, resulting from decreased diversity of environmental and human microbiota, would increase the risk of allergic, autoimmune, and other inflammatory, immunological, and metabolic conditions.^237^

The microbiota hypothesis considers that the balance of the intestinal microbiota (eubiosis) with adequate biodiversity becomes a dominant factor of epigenetic adaptation and prevents the development of allergies and allows resilience to change arising from environmental challenges.^113^, ^162^

Adaptation to modern life, characterized by accentuated and disordered population growth; increased urbanization, industrialization and environmental pollution; increased use of monocultures, pesticides and antimicrobials; increased consumption of processed, energy-dense, nutrient-poor and less diverse diets; and decreased physical activity, may result in substantial dysregulation of the environmental and human microbiota in many countries, potentially explaining (at least partially) the current global pandemic of chronic Non-Communicable Diseases (NCDs), including allergic diseases.^238^

Increasing levels of air pollutants are associated with a lower abundance of Corynebacterium and increasing levels of colonization by pathogens such as Hemophilus influenzae, Moraxella catarrhalis, Streptococcus pneumoniae and Pseudomonas aeruginosa and Acinetobacter baumannii, altering the incidence and clinical course of respiratory infectious diseases, leading to excess morbidity and mortality due to antimicrobial resistance.^239^

Indoor microbiome exposure is associated with asthma, rhinitis and eczema. Increased exposure to Aspergillus subversicolor, Collinsella, and Cutibacterium were positively associated with rhinitis.^240^ Abundance of *Staphylococcus* spp. has also been reported in the anterior portion of the nostrils and in the hypopharyngeal region of children and adolescents who have AR, passive exposure to tobacco smoke and allergic rhinoconjunctivitis.^241^

In recent decades, several shortcomings of the hygiene and biodiversity hypotheses have been discussed, suggesting that these hypotheses do not fully explain the increase in allergic diseases.^130^

Recent publications highlight that climate change, air pollution, microplastics, tobacco smoke, changes and loss of biodiversity, changes in eating habits and the microbiome, are factors that disrupt the epithelial barriers of the skin and mucosal surfaces. These ruptures have been associated in recent decades with an increase in the prevalence and severity of allergic and inflammatory diseases, including AR and CRS.^242^

The epithelial barrier, with its physical, chemical and immunological properties, is the first line of defense of the innate immune system. It covers the gastrointestinal system, the skin, the urogenital system and the respiratory tract. Epithelial cells are tightly adhered to each other by tight junctions and are well-organized with the contribution of mucus and microbiota. Its immunological functions include eliminating particles and activating immune cells through the production of antimicrobial peptides and cytokines.^243^

In addition to its antimicrobial action, the epithelial barrier is also essential for prompt tissue repair. Once compromised, in addition to tissue damage, an inflammatory state occurs that worsens epithelial damage. Healthy tight junctions prevent the entry of foreign substances, while the broken barrier allows passage to both sides, whether through the entry of foreign substances, the exit of immune cells from the subepithelium to the surface or the translocation of microbiota to deeper tissues, favoring the inflammation.^244^

Consequently, an inflammatory microenvironment disrupts the epithelial barrier and epithelial stem cell regeneration. This sequence of events determines a local or systemic inflammatory state that can be the cause of many disorders related to the immune system. Local epithelial damage to the skin and mucosa can determine the development of type 2 inflammation, which manifests as atopic dermatitis, asthma, AR and eosinophilic esophagitis.^245^, ^246^

Several features of the epithelial barrier are impaired in AR and may contribute to the inflammatory cycle at distinct levels of the epithelium. Understanding the underlying factors that affect the integrity of epithelial barriers is essential to determine preventative measures or effective treatments to restore their function and thus contribute to reducing the prevalence of allergic diseases worldwide.

#### Vitamin D

Vitamin D (VD) is a fat-soluble prohormone with endocrine, autocrine and paracrine functions, being paramount in bone metabolism.^247^ It increases the absorption of phosphorus and calcium from the intestine, reducing their excretion by the kidneys and promoting osteogenesis.^248^ The National Institutes of Health and Nutrition Research (NIHNR) revealed that the average serum 25(OH)D3 level fell from 30 ng/mL in 1988–1994 to 24 ng/mL in 2001–2004. Population studies have observed that both VD deficiency and allergic diseases have increased year after year, due to greater Westernization,^248^ with a sedentary lifestyle, with less sun exposure and lower skin metabolism of VD.^249^

The term VD refers to the presence of two types of vitamin, D2 (ergocalciferol) and D3 (cholecalciferol). Both forms can be obtained through food, but insufficiently. VD is present in small quantities in butter, peanuts and eggs, and is present in copious quantities in fish and fish oil. Additionally, both cow's milk and human milk are deficient in VD.^250^

The first step in the endogenous production of VD involves the absorption of Ultraviolet radiation (UVB) by 7-dehydrocholesterol under the skin, producing previtamin D3, which is converted into vitamin D3.^251^ This suggests that in addition to being a vitamin, VD acts as a prohormone. The metabolism of vitamin D3 depends on the ability of UVB photons to penetrate the skin. It was observed that the entry of photons into the skin is significantly reduced when there is dark pigmentation in the skin,^252^ senescence and obesity.^253^

The portion of the VD that is directly dosed to measure vitamin D3 in the blood is 25(OH)D3, with a half-life of two weeks.^252^

In circulation, cholecalciferol and ergocalciferol are converted in the liver by the action of vitamin D-25-hydroxylase (CYP2R1) to 25-hydroxyvitamin D or calcifidiol [25(OH)D]; subsequently, 25(OH)D undergoes a second hydroxylation, by the enzyme 25-hydroxyvitamin D-1α-hydroxylase (CYP27B1), into the active and bioavailable form of vitamin D (1,25-dihydroxyvitamin or Calcitriol ‒ CT) [1,25 (OH)2 D].^254^ This reaction occurs mainly in renal tissue. 1,25 (OH)2 D exerts its function by binding to the Vitamin D Receptor (VDR), expressed in the cytoplasm of cells, forming the hormonal complex VDR-RXR (retinoid × receptor).^255^ Once in the nucleus, the complex regulates the expression of several genes, increasing or decreasing their functions.^256^ A 1.25 (OH)2 D has d affinity and binding to the VDR receptor 1000 times greater than 25(OH). CYP27B1 hydroxylase and VDR are expressed in various tissues such as the pancreas, kidneys, muscles, liver, parathyroid glands, breasts, colon, among others, and cells, such as activated macrophages, microglia, and keratinocytes, hence their actions beyond the skeletal tissues, including the immune system.^257^

Several subtypes of allelic polymorphisms are identified in the gene that originates VDR, and their association with different allergic conditions is being investigated.^258^, ^259^ However, studies regarding the role of VDR polymorphisms in AR are limited.

VD plays a key role in innate immunity, stimulating the production of Pattern Recognition Receptors (PRRs), antimicrobial peptides and cytokines. It can also prevent the maturation and activation of dendritic cells, as well as the differentiation of monocytes to macrophages. The conversion of 25-OH-D3 to 1,25(OH)2-D3 is stimulated during infection as a result of increased CYP27B1expression in activated macrophages. There is then greater antibacterial activity of macrophages and monocytes via VDR-RXR. Through the action of VDR receptors, 1,25 (OH)2 D3 increases the cytotoxic activity of NK cells and Lymphoid Cells (ILCs).

Regarding acquired immunological responses, VD induces Th2 type responses and the inhibition of Th17 and regulatory T lymphocytes.^260^ 1,25-(OH)2 D3 inhibits the synthesis of pro-inflammatory cytokines IL-2 and IFNγ, as well as Th1-mediated responses. 1,25-(OH)2 D3 also causes an increase in the production of Th2-type anti-inflammatory cytokines (including IL-3, IL-4, IL-5, and IL-10), while there is less production of cytokines pro-inflammatory IL-9 (Th9) and IL-22 (Th22).261 IL-9 is essential for chemotaxis mainly of mast cells and is produced by regulatory T cells. B lymphocyte homeostasis is directly impacted by membrane VDR expression. Clinical significance can be seen in the reduction of autoimmune diseases in patients with normal vitamin D levels.^262^ In fact, 1,25(OH)2 D3 suppresses the proliferation of B lymphocytes, modulating their response. The vitamin also suppresses and modulates T lymphocyte responses by lower expression of CD40, CD80, CD86, and MHC class-II on antigen-presenting cells.^263^

Furthermore, by participating in the regulation of the function and composition of the intestinal microbiome, VD and its levels play a relevant role in the pathogenic processes of immunological diseases related to the dysbiosis of this system.^264^

Although many studies show a strong correlation between VD and the innate and acquired immune systems, suggesting that low levels of VD may contribute to immune dysregulation, more clinical studies are needed to determine the precise impact of VD supplementation on the pathophysiology of AR.

## Clinical picture

### Diagnostic features

#### Etiological diagnosis

The clinical suspicion of AR begins with the medical interview, with the main symptoms being runny nose, sneezing, itching and periodic nasal obstruction, in the absence of viral infections. Eye symptoms of itching, hyperemia and tearing may also occur. Other potential symptoms include itching on the palate, in the ear canal, post-nasal secretion and cough.^265^ Anamnesis is essential for the suspected diagnosis of AR and the identification of the phenotype involved, enabling an appropriate treatment plan.^266^, ^267^

Most patients with AR have their onset in childhood. When they occur in adulthood, other rhinitis phenotypes should be investigated. Patients with AR often have a family history of allergies and, when they present symptoms upon exposure to domestic animals, sensitization to them may be observed (prick tests or determination of specific IgE in vitro). In contrast, patients with non-allergic rhinitis are older, mostly middle-aged women with obstructive complaints and posterior rhinorrhea, often accompanied by recurrent headache and olfactory dysfunction.^268^

Symptoms of AR appear quickly (within the first hour) after allergen exposure.^269^ Late manifestations of AR include nasal congestion, hyposmia, nasal hyperreactivity, and post-nasal drainage. The time after exposure, location of symptoms, factors that alleviate or exacerbate them (smoke, inhalation of chemical elements) and triggers such as pollens, household dust, fungi, dust mites and pets are key elements.

AR is often associated with other allergic conditions such as asthma, conjunctivitis and atopic dermatitis, with characteristic symptoms. The identification of comorbidities is essential for simultaneous treatment, for example, of asthma, allergic conjunctivitis, atopic dermatitis, mouth breathing and consequent sleep disorders. Patients with RA and mouth breathing may experience learning and concentration disorders, often accompanied by changes in sleep architecture. Hearing changes (conduction) may occur due to chronic rhinosinusitis, secretory otitis media and adenoid hypertrophy.^266^

Additionally, AR is a risk factor for the onset of asthma, and on the other hand, poorly controlled severe AR affects asthma control.^270^

The physical examination must include anthropometric measurements and a complete clinical assessment with examination of the skin, ears, nose, mouth, the oropharynx and chest. Typical findings include the double infrapalpebral line of Dennie–Morgan, the horizontal line on the nasal dorsum (nasal groove, allergic greeting), the presence of infrapalpebral darkening (vasodilation and venous congestion, allergic dark circles), conjunctival hyperemia and mouth breathing, with changes to specific skeletal muscles on the face (mouth breathing).^271^

Anterior rhinoscopy can be performed with the help of a focused headlight, with visualization of the head of the inferior turbinates, which may be enlarged, pale (chronicity of the inflammatory process) and in cases of acute infection, hyperemic. There may be clear or dense and dark secretion in cases of infection in addition to the visualization of large nasal polyps (grade IV), foreign bodies, deviated nasal septum (anterior), valve collapse and mucosal edema.^271^

Endoscopic visualization is of great value, especially in cases of symptoms refractory to treatment. In these cases, several anatomical abnormalities can be seen, such as the presence of nasal polyps, the volume of adenoids and the presence of nasal lesions. In some cases, academically, functional assessment (rhinometry and rhinomanometry) of bilateral nasal patency complements and guides the best management.^272^

Regarding etiology, anamnesis often guides the search for sensitizations, as in the case of seasonal symptoms or symptoms caused by inhalation of dog or cat allergens. In these cases, the patient reports a direct and rapid relationship between contact and the appearance of symptoms, which does not occur, for example, with chronic inhalation and in small quantities of house dust mite allergens.^273^ As studies point to the importance of sensitization to mites in patients with AR, this studied must be carried out, regardless of clinical history. It is also common for the patient to complain of symptoms when inhaling airway irritants, and this occurs due to the direct stimulation of autonomic nervous system fibers, present in the nasal cavity.^274^

When investigating the etiology of AR, the patient with rhinitis must undergo verification of allergic sensitization using immediate hypersensitivity skin tests (IHST or skin prick test, SPT) or laboratory methods for in vitro measurements of specific serum IgE (e.g., ImmunoCap®) to aeroallergens. The determination of specific IgE to inhalant allergens is necessary to define the patient's atopic condition and, once associated with anamnesis and physical examination, the diagnosis of AR is inferred.^275^ The diagnosis of sensitization to aeroallergens allows for targeted therapeutic options, such as specific immunotherapy.

Some patients with symptoms characteristic of AR may present negative SPT to aeroallergens. Ruling out the possibility that the patient has received antihistamines, is under five years of age and is sensitized to other aeroallergens not present in the chosen battery of extracts, other rhinitis phenotypes should be investigated: non-allergic or local AR.^276^

If the IHST is negative, but the clinical history suggests the involvement of aeroallergens, Intracutaneous Testing (ICT) can be performed. However, in practice this does not happen, since ICTs have high sensitivity, but low specificity, compared to IHST.^276^

Regarding differential diagnoses, it is essential to rule out other diseases with symptoms similar to those of AR. Infectious rhinitis often occurs on the background of viral infections of the upper respiratory tract. The spectrum of non-allergic and Non-Infectious Rhinitis (NAR) comprises conditions that manifest as rhinitis due to exposure to irritants such as cigarette smoke, environmental pollutants, hormonal dysfunction (subclinical hypothyroidism), exposure to medications and idiopathic rhinitis. The differential diagnosis may include Local AR (LAR), dual AR (with systemic sensitization to one allergen and only local to another), mixed rhinitis (allergic and non-allergic), anatomical abnormalities, adenoid hypertrophy and other conditions, which can present similar manifestations. These include inborn errors of immunity, cystic fibrosis and primary ciliary dyskinesia.^21^ The presence of unilateral symptoms, nasal obstruction as an isolated symptom, mucopurulent posterior nasal discharge, pain, epistaxis and isolated anosmia directs the differential diagnosis to other clinical entities.

In conclusion, patients with AR require a proactive and individualized assessment, combining accurate etiological diagnosis with individualized therapy. Etiological diagnosis, identifying the allergen or allergens that are clinically relevant or causing allergic symptoms, is essential for the prescription of personalized treatment with allergen-specific immunotherapy.

#### Immediate hypersensitivity skin tests

The Immediate Hypersensitivity Skin Test (IHST) is the main in vivo method recommended by international guidelines for diagnosing atopy.^277^, ^278^ It has high sensitivity and specificity. The IHST modified by Pepys is used as a reference to this day.^279^

In these tests, small portions of natural or synthetically prepared allergens are deposited on the patient's skin (on the ventral surface of the forearm). Next, a superficial puncture is made, sufficient for the penetration of allergens into the subcutaneous tissue, where in contact with mast cells, if sensitized, inflammatory mediators will be quickly released, with the formation of erythema and formation of local papules.^280^ Controls must be applied. positive (histamine 0.1%) and negative (saline), to rule out false positive and negative results. The size of the papule can be measured after 15 min, and the result is positive when the mean diameter of the papules greater than 3 mm of that produced by the negative control.^281^

The choice of allergenic extracts to be tested must be selective; In the case of AR, aeroallergens related to the patient's history will be chosen (e.g., domestic animal allergens) or if there is no clinical suspicion, the allergens of the main house dust mites found in Brazil must be evaluated: Dermatophagoides pteronyssinus (Dp), Dermatophagoides farinae (Df) and Blomia tropicalis (Bt).^282^ In addition to these, cockroach, fungus and pollen allergens can be tested, depending on the region of Brazil. Therefore, the allergens used for IHST must be individualized and selected, based on the prevalence in the geographic area where the patient lives and goes to.

Mites of the genus Dermatophagoides represent the most important sources of allergens in allergic respiratory diseases, with great similarity between their allergens, leading to high cross-reactivity between specific IgEs for Dp and Df.^283^ Der p 1 and Der p 2 are considered major Dp allergens, that is, 80%–85% of individuals sensitized to Dp have specific IgE to at least one of these allergens.^284^ In 2013, Weghofer and colleagues identified Der p 23 as a new major Dp allergen located on the surface of dust mite fecal particles.^285^

Although IHST can be performed at any age, children under one year of age may not show positive reactions to aeroallergens. Children with seasonal respiratory allergies only test positive after two seasons of exposure. To avoid false negative results, first generation antihistamines must be suspended between 36 and 48 h before the test. On the other hand, second-generation antihistamines should be suspended one week before the test, always observing the reactivity obtained with histamine (positive control). Positive IHST mean that the patient is sensitized to the allergens in question, is an atopic patient and does not necessarily offer a diagnosis of allergic disease.^286^ For the diagnosis of allergic disease it is necessary to combine a) Positive history; b) The presence of specific IgE antibodies and c) Demonstration that the symptoms are the result of IgE-mediated inflammation. For the diagnosis of AR, it is necessary to have a medical history consistent with the results of IHST and/or specific serum IgE.^287^

#### Determination of total and specific serum IgE

Total serum IgE is increased in atopy but it is not specific for any of the atopic diseases. Additionally, except for allergic bronchopulmonary aspergillosis, there is no relationship between the IgE value and disease severity. Therefore, total IgE measurement is not necessary for the diagnosis or monitoring of AR. It may, however, be useful to point out the possibility of atopy in the face of a suggestive respiratory condition.^20^, ^274^

The determination of specific serum IgE by serological immunoassays is relevant for the diagnosis of atopic diseases, even if it is not specific to AR. The use of purified and recombinant allergens promotes greater sensitivity, specificity and accuracy of this dosage.^288^

Serum quantification shows good correlation with in vivo skin testing for the diagnosis of AR; however, it is accepted that skin tests are more sensitive and, therefore, first-line for diagnosis.^20^, ^274^

Advantages in relation to skin tests are the non-interference of medications and clinical conditions such as dermographism, as well as greater safety, without the risk of anaphylaxis. However, measuring specific IgE is more costly and requires blood collection.^20^

There are five types of assays for identifying specific IgE, some qualitative and semiquantitative, rarely used. Second generation quantitative assays (ImmunoCAP® and Immunolite®) are the most commonly used. There is no good correlation between the results obtained by the different quantification methods, and it is essential to pay attention to the method used when interpreting the results.^289^

There are single-platform (quantitative) assays, which allow the physician to choose the allergens to be tested according to the patient's history, as well as multiple-platform (semi-quantitative) assays that provide results for several pre-selected allergens.^20^, ^289^

However, it is important to emphasize that the presence of positive specific IgE means the presence of sensitization and not necessarily the presence of an allergic disease. The presence of characteristic symptoms of AR when identifying allergen sensitization allows the diagnosis to be confirmed and treatment to be directed.^289^

The relationship between specific serum IgE and total serum IgE has been proposed as a marker of good response to allergen-specific immunotherapy. A cutoff value of 16.2% was able to predict success in immunotherapy with sensitivity of 97.2% and specificity of 88.1%.^290^ However, these results could not be reproduced and it is still necessary to explore and validate this finding.^291^

#### Diagnosis by allergenic molecular components (microarray)

In the early 2000s, proteomic microarray platforms (chips) for the diagnosis of allergic sensitization emerged. The ImmunoCAP ISAC platform initially composed of 74 proteins (components), currently consists of 112 allergens. There is also a platform (ALEX) made up of 282 proteins, in addition to a platform based on immunoblotting (MAST). Highly purified natural or recombinant molecules are used, allowing the identification of specific IgE with a small amount of blood, but without being able to select the allergens to be tested according to the patient's condition. Another disadvantage is the cost, which is still high in our country, in addition to the production of a large amount of information that may not be easy to interpret. There is no good correlation with the IgE dosage for allergenic extracts, and the sensitivity of single platforms is considered greater than that of multiple platforms.^274^, ^289^

In relation to mites, most patients are sensitized to Der p 1 and/or Der p 2, and the response to immunotherapy seems to be better when there is sensitization to just one of them.^292^ Sensitization to Der p 23 is implicated in greater risk of developing asthma.^293^ Der p 10 is a tropomyosin and indicates the possibility of cross-reaction with crustaceans and molluscs.^294^

Dog and cat allergen extracts for testing and immunotherapy are heterogeneous and may not contain the most relevant allergens for some patients with animal allergies. In this sense, knowing the sensitization to specific components can help predict the response to specific immunotherapy.^295^, ^296^

Therefore, the investigation of specific IgE for the components enables us to better define sensitization (differentiating cross-reactions from co-sensitization), predict the severity of symptoms, better select the indication for specific allergen immunotherapy, as well as better predict the response to it, being particularly relevant in polysensitized individuals. However, due to the cost and difficulty of access, the wide use of this method in clinical practice is currently not recommended.^20^

#### Nasal provocation test

The Nasal Provocation Test (NPT) is considered a simple and safe procedure, capable of objectively evaluating, in a safe and controlled environment, the responses of the nasal mucosa after contact with substances and/or external stimuli applied locally.^297^ It is considered a one of the only tools capable of establishing a direct correlation between exposure to suspected allergens and clinical symptoms, that is, defining the diagnosis of allergy to a substance and not just sensitization to it. Despite being simple, NPT must be carried out in an appropriate environment, with a well-trained technical team and using well-established protocols.^64^, ^298^

We can classify NPT as nonspecific or specific, depending on the characteristic of the stimulus that will be used. The first use pharmacological agents such as histamine, methacholine or physical stimuli (e.g., cold air), capable of triggering a nasal response in all individuals (allergic and non-allergic) in some concentration. When positive responses are triggered at low concentrations, nonspecific NPT only helps in the diagnosis of nasal hyperreactivity, a common feature in patients with rhinitis (approximately 60%),^69^ but not pathognomonic of the disease.^299^

Specific NPT (sNPT) use stimuli such as medications, pollens and mites, triggering a positive response only in patients allergic to the antigen used. The main indications and contraindications of sNPT are presented respectively in Table 3, Table 4.^64^, ^298^ Validated and individualized protocols for each allergenic extract are essential for carrying out and correctly interpreting the procedure,^64^, ^298^ as some allergens can act irritatingly on the nasal mucosa, not making it possible to diagnose allergy as they would trigger a response in all patients (sensitized and non-sensitized).^64^, ^298^

**Table 3:** Main indications for specific Provocation Tests.^64^, ^298^

Help understand the pathophysiological mechanisms of rhinitis;
Investigation of drug efficiency and mechanism of action;
Confirmation of the AR etiology;
Confirming the relevance of specific allergens in polysensitized patients;
Follow-up of treatment with specific immunotherapy; and
Diagnostic confirmation of local and occupational allergic rhinitis

**Table 4:** Main contraindications of specific Provocation Tests.^64^, ^298^

Local infectious processes (rhinitis or sinusitis);
Worsening of chronic cardiopulmonary conditions;
Anaphylaxis prior to the substance being tested;
Gestation;
Nasal surgery less than 6–8 weeks ago;
Use of medication to control rhinitis in the last two weeks (antihistamines or nasal corticosteroids);
Recent vaccination
Children under 5years of age

To date, NPTs have remained as tools for laboratory investigation and research, but with the description of Local Allergic Rhinitis (LAR), the development of multiple NPT protocols (in which a series of different allergens are applied sequentially on the same day) may simplify and improve screening of patients with this specific rhinitis phenotype.

### Nasal cavity assessment

#### Nasal cytology

Simple and easy-to-perform exam that detects the cellularity of the nasal mucosa at a given moment. It allows the identification of normal ciliated and mucinous and inflammatory cells (eosinophils, neutrophils, lymphocytes, mast cells), fungi and bacteria, thus facilitating the differentiation of numerous pathological conditions of the nasal mucosa or the effects of different stimuli on it. They should be requested when there is a need to evaluate specific inflammatory or biological markers.^300^ It can help in cases of non-allergic rhinitis to check for local allergic rhinitis or even in cases of allergic rhinitis to rule out mixed rhinitis. To define Non-Allergic Rhinitis with Eosinophilic syndrome (NAER), the cutoff point values have not yet been defined.^20^

The normal nasal mucosa is made up of ciliated cells, mucinous cells, striated basal cells and rare neutrophils.

Pathological aspects: The correlation of findings and the clinical aspects are fundamental.^300^

A Infectious rhinitis: substantial number of neutrophils, with intra or extracellular bacteria and reduction of hair cells;
B Allergic rhinitis: Intense infiltrate of eosinophils and mast cells, related to allergen exposure;
C Non-infectious non-allergic rhinitis:
D With Eosinophilic Syndrome (NAER): eosinophilic infiltration (50–70%), which may be associated with nasal polyposis, asthma or sensitivity to Acetylsalicylic Acid (ASA) or Non-Steroidal Anti-Inflammatory Drugs (NSAIDs);
E 2 - Without eosinophilic syndrome: pseudo-allergic symptoms in the presence of non-specific stimuli (cold air, humidity, cigarette smoke, nasal irrigation, smells, etc.) with the presence of neutrophils, mast cells or associated
F Mixed rhinitis: cytology is important for understanding cell predominance and directing treatment in the most appropriate way.

#### Bacteriological and bacterioscopic examination

The purpose of bacteriological and bacterioscopic examination is to identify pathological bacteria in the nose or paranasal sinuses, with consequent appropriate antimicrobial treatment.^3^

Nasal secretion collection for culture exams can be performed by several methods: blowing, swab, nasal wash, middle meatus swab under endoscopic vision for sinus secretion, direct collection from the maxillary sinus. When blowing the nose with secretion, there is high agreement with nasal swab collection (>90%). However, if there is no secretion, this rate reduces to <50% agreement. Comparing direct sinus collection with swabs under nasal endoscopy, high agreement was observed between the two methods. Studies that evaluated nasal lavage with a middle meatus swab, guided by an endoscope, demonstrated a greater assessment of bacteria and anaerobic pathogens, appearing to be more sensitive for detection in cases of less bacterial contamination.

In patients with chronic rhinosinusitis who do not respond to the established treatment, a microbiological study may be requested.

Nasal cytology is not effective in distinguishing non-bacterial infections from bacterial rhinosinusitis, and positive cultures for bacteria from the nose or sinuses often represent colonization rather than infection.^36^

#### Specific nasal permeability tests

Various aspects of nasal obstruction can be assessed using functional nose exams such as peak inspiratory flow, computerized rhinomanometry and acoustic rhinometry. In addition to evaluating nasal airflow, they can be used to monitor the response to medical or surgical treatment.^301^

• Computerized rhinomanometry

The simultaneous measurement of nasal airflow and the pressure required for flow defines nasal resistance to air passage.^301^ Types of measurements: anterior or posterior, active or passive, with active anterior rhinomanometry being the most frequently used. Nasal resistance (R = P/F) is measured in Pa/cm^3^ per sec. The more obstructed the airway is, the greater pressure is necessary to generate a certain flow.

Initially measured at a standard of 150 Pa, the latest consensus recommended the use of four-phase rhinomanometry in which resistance is calculated using hundreds of resistances recorded continuously throughout the respiratory cycle.^302^ Despite the differences between measurement techniques, there does not appear to be significant differences between the results.^303^

Reference values are defined for children and adults, as well as normal values for four-phase rhinomanometry.^20^

• Acoustic rhinometry

Evaluates the geometry of the nasal cavity. Through the acoustic reflection of sound waves that pass through the nasal cavity, it measures areas and volumes at different points, which provides information about the structure and dimensions of the nasal cavity.^301^

Acoustic rhinometry is more useful in research than as a clinical diagnostic tool.^20^

• Peak nasal flow (PNF)

Cheap, fast, portable and simple method for nasal assessment. Performed with three deep and quick breaths, through the nose and with the mouth closed. The measure with the highest value will be considered the PNF. Normal values for adults and children, as well as unilateral PNF values for adults, are already defined.^20^

Although they evaluate various aspects of nasal function, a correlation between the tests is observed. Thus, PNF and four-phase Rhinomanometry showed a moderately significant correlation, but PNF showed a significant correlation with nasal symptom scores.^304^ Thus, nasal patency tests can be used individually or combined to provide objective information about nasal function.

#### Image exams

Imaging exams, except for the diagnosis of sinonasal diseases (Computed Tomography – CT ‒ in rhinosinusitis/polyposis and magnetic resonance imaging in tumors) and associated rhinopharynx diseases (simple cavum radiography for adenoid hyperplasia), are not useful in AR.^305^ Today, experience with third-generation cone-beam CT technologies provides precise information about bones, making it possible to demonstrate anatomical variants involving bony sinonasal structures. However, it is not yet possible to make a qualitative assessment of soft tissues, as there are no Hounsfield levels (relative quantitative measure of radiodensity, derived from the absorption/attenuation coefficient within the tissue to produce a gray scale in the image) in the Cone-beam CT, as we have in conventional CT techniques. However, this is a new area of research and its application is developing in an interesting way, especially for inflammatory-allergic soft tissue diseases. An initial study used Micro-CT to quantify the edema of the sinonasal mucosa after nasal provocation with methacholine in rats. A dose-dependent edema of the nasal mucosa was observed with the use of methacholine in genetically allergic rats, which did not happen in control rats, and this edema was completely abolished with the use of corticosteroids pre-nasal challenge.^306^

### Complementary tests

#### Nasal mucosa biopsy

Regarding nasal biopsy, although informative, it is not clinically indicated. However, initial evidence points to a high correlation between brushing the nasal mucosa and biopsy of the inferior nasal turbinate in order to detect allergen-specific IgE, being a less invasive procedure. In the future, brushing the anterior third of the nasal fossa (lateral surface of the nasal turbinate and juxtaposed septum) could be indicated as an alternative method of obtaining a sample of the nasal epithelium, which could be useful, for example, in the diagnosis of local allergic rhinitis.^307^

#### Smell assessment tests

Olfactory changes are quite common symptoms in sinonasal and neurodegenerative diseases and, when present, have a significant impact on quality of life. The recognition of such changes, whether qualitatively or quantitatively, is fundamental for the diagnosis, treatment and follow-up of the underlying disease causing these olfactory disorders.

Old studies have shown that AR affects the sense of smell in many patients, including children. The global prevalence of olfactory dysfunction due to AR has been reported to be 21.5%–23%.^308^, ^309^, ^310^ A national study retrospectively analyzed outpatients with primary complaints of olfactory disorders and found 19% of AR as the cause.^311^ In studies using According to the most recent ARIA classification, patients with persistent moderate/severe AR were found to have a higher prevalence of hyposmia.^312^, ^313^ Although olfactory dysfunction does not appear to be very severe in patients with AR, its presence appears to increase with the severity of the disease.^314^

Given the above, it is clear that documentation of olfactory function is of fundamental importance in cases of patients with AR. The systematic application of a test to evaluate the olfactory thresholds of patients with AR who complain of hyposmia, or anosmia is mandatory not only as a definitive diagnosis, but also to monitor and verify improvement after treatment.

### Subjective assessment instruments

#### Visual analogue scale (VAS)

The application of VAS can be useful for how the patient perceives the loss of smell, quantifying it between 0 and 10, with “0” being absence of smell and “10” normal smell or as before the disease. It is not always possible to rely on a subjective self-report from the patient, but rather, to prove the patient's complaint objectively when performing the test. It should always be done before nasofibroscopy, either due to possible local irritation generated by the exam, or due to the anesthetic used by some otorhinolaryngologists before this procedure.

#### Tests validated in Brazil

(1) UPSIT test: Currently, the most widely used test globally is the UPSIT (University of Pennsylvania Smell Identification Test), already validated in Brazil.^315^

UPSIT consists of four cards with 10 odors each. The patient uses an object to scrape a silver strip that contains the odor to be detected, brings the block one centimeter from the nose and responds, obligatorily, among four alternatives, choosing a forced option among four possibilities. The score ranges from 0 to 40 and the olfactory function is classified as anosmia, microsmia or hyposmia (mild, moderate and severe). A score below 5 indicates possible simulated olfactory loss.

The patient can perform it himself or with the help of an examiner and can detect simulators and classify the degree of olfactory loss, important advantages. The price still makes it difficult to use in our country, especially in medical residency services that operate through the Public Health Care System and in other services with financial limitations, but it is currently more accessible.

(2) Olfactory threshold and odor identification test developed by CCCRC (Connecticut Chemosensory Clinical Research Center): The Connecticut or CCCRC test includes a threshold test and an odor identification test and has been validated for our population.^316^ The threshold test is performed by presenting the individual with two identical 60 mL bottles simultaneously. One bottle contains only distilled water and the other contains a solution of butanol and distilled water. Individuals are instructed to occlude one nostril and position the vials, one at a time, in the opposite nostril, thus choosing which vial has something other than water. If the individual does not get it right, bottles with the most concentrated butanol solution are presented consecutively together with the bottle of water until the individual gets it right. Two consecutive hits of the bottle containing the odorant determines the minimum concentration defined as the individual's olfactory threshold for the tested nostril. The other nostril is tested separately. The score ranges from 0 to 7 points, with 0 being the individual who was unable to identify the bottle containing the odorant, and 7 being the individual who identified the odorant in the bottle with the lowest concentration. The odor identification test is also carried out unilaterally by presenting 7 bottles with odors present in the daily lives of the Brazilian population: coffee, cinnamon, Johnson & Johnson® talc, peanut paste, chocolate, Palmolive® soap and mothballs.

The individuals receive a list of substances contained in the test and distracting substances (peanut paste, pepper, talc, cigarettes, soap, tires, coffee, mothballs, cinnamon, wood, garlic, chocolate, tomato sauce, sardines, onions and Vick®) and mark the one they found correct for each bottle presented. Each odor can be presented more than once to the individual, reducing errors due to cortical issues. The function of the trigeminal nerve is tested at the end of the test by presenting Vick® to the individuals, but the identification of this substance was not included in the final score. The final score of the identification test is obtained by scoring from 0 to 7, according to the number of correct odors. The final score composed of the threshold test and the identification test is obtained by the arithmetic mean of the final score of both tests and varies from 0 to 7, classifying each nostril separately: normal: 6.0–7.0; mild hyposmia: 5.0–5.75; moderate hyposmia: 4.0–4.75; severe hyposmia: 2.0–3.75 and anosmia: 0–1.75.

The cost of raw materials to create a kit is approximately 30 reais. Considering that each kit can be used for multiple patients, with reagents being replaced ideally every 3 months to renew the odor dispersion capacity of the substances present in the test, the cost per test is extremely low. The ease of transport, combined with the possibility of it being applied in any environment, make the CCCRC test a practical and easy-to-install assessment. A relevant advantage is the fact that it measures both the threshold and the identification of odors. One of the disadvantages in relation to UPSIT is that the CCCRC cannot be self-applied, it requires a trained person, taking on average 15 min to apply.

(3) Multiscent-20 digital odor identification test: Multiscent-20^317^, ^318^ is a tablet that has an odor storage and release system. It is a portable hardware device with a touch screen, integrated with a system of microcapsules capable of presenting up to 20 different odors, through a flow of dry air released at the opening of the odor dispenser. Capsules are loaded through an insertion port on the back of the device. The software application presents, controls and records responses in an automated way. The digital odor identification test uses the mandatory response paradigm among four alternatives. The olfactory assessment begins with the individual receiving instructions presented by an “avatar”, as described below: (1) This assessment consists of a test containing 20 odors. (2) Sit in a comfortable position and when releasing the smell, placing the device 10 cm away from one’s face. (3) The next screen will show the phrase “This smell looks like” and with four answer alternatives. Read all options before pressing the “Release smell” button. (4) When pressing the “Release smell” button, a small opening on the top front of the device will release the odor for five seconds. You can press the “Release smell” button up to two times. (5) After noticing the odor, select the option corresponding to the odor you smelled, and press the “NEXT” button to continue. If the odor you smell is not among the alternatives, select the alternative that comes closest. (6) The number of correct answers will be displayed after completing the test.

The number of correct answers corresponds to the identification test score and the olfactory function is classified as normosmia (≥15 points), hyposmia (14 to 11 points) and anosmia (≤10 points).^318^

(4) Assessment of smell in children: Although this form of objective assessment of smell is also important in children, we encounter major obstacles, especially in children under five years of age: the tests are too complex for the pediatric age group; children may not yet have been introduced to the odors presented in the tests; the assessment is very long, making the test very tiring for children in certain age groups; the tests were not validated for the proposed age range.^319^ As the identification test includes other skills, such as attention, memory and linguistic processing, it can be understood and performed in older children, from the age of five. Discrimination and threshold tests are not as dependent on age as they do not involve olfactory memory.^319^

In 2015, the Sniffin’ Sticks, a 16-odor identification test, was cross-culturally adapted for the pediatric population in Brazil. Children between three and 18 years old were selected and it was observed that older children performed better on the test, and that preschool-aged children had difficulty understanding the exam.^320^

(5) Assessment of interference with daily activities: Although AR does not expose the patient to a risk of death, regardless of its intensity, it can compromise Health-Related Quality of Life (HRQoL), either due to the presence of comorbidities, or by causing school/work absenteeism, school/work presenteeism, impairment quality of sleep and quality of life.^19^, ^20^, ^153^

Over the years, several instruments and scores have been developed to obtain an objective measure capable of quantifying the repercussions of a given disease or its treatment on the patient's life. HRQOL is assessed by physical, psychological and social components that can be affected by the individual's perception of their disease and clinical conditions.^321^, ^322^ Individual experiences with the disease can influence HRQOL more than its severity.^323^

In patients with AR, quality of life and sleep have been the most evaluated.

(a) Health-related quality of life: High-quality evidence is increasingly accumulating that confirms the impact of AR on patients' Quality of Life (QOL), whether assessed by generic instruments (Short Form 12 [SF-12] and 36 [SF-36]),^324^, ^325^ whether by those specific to the disease,^326^, ^327^, ^328^ as well as in evaluating the benefit of established treatments (intranasal corticosteroids, oral antihistamines, allergen immunotherapy, among others).^329^, ^330^, ^331^

QoL questionnaires are short, easy to understand, self-administered and simple to execute.^332^ As mentioned previously, disease-specific instruments have increasingly been used to assess QoL in AR. The most used instrument is the Rhinoconjunctivitis Quality of Life Questionnaire (RQLQ), developed by Juniper and collaborators.^333^ This version was followed by one intended for adults,^334^ a reduced version: the MiniRQLQ,^335^ as well as versions for adolescents (12–17 years).^336^ and children (6–12 years old) the PRQLQ (Pediatric Rhinoconjunctivitis Quality of Life Questionnaire).^337^

In Brazil, the pediatric version of the RQLQ was translated, adapted and validated into Portuguese (Brazilian culture) and used to evaluate children and adolescents with AR, before and after treatment with intranasal corticosteroids.^338^

Most questionnaires that assess QoL in patients with AR do so in association with conjunctivitis, however few do it for more severe forms.^339^, ^340^, ^341^ Ferreira and collaborators translated, adapted and validated the Quality of life in children with vernal keratoconjunctivitis questionnaire (QUICK) into Portuguese (Brazilian culture) and applied it to adolescent patients with different severities of allergic conjunctivitis. Worse quality of life was observed among patients with more severe forms.^341^

(b) Quality of sleep: Sleep-related Disorders (SD) are common, with a prevalence of between 25% and 40% in children, especially among patients with nasal problems. Because they are not frequently reported, they are not frequently treated. Normally, parents do not value the symptoms and/or are unaware of them and doctors fail to investigate them.^20^, ^342^, ^343^ As it is an objective measure, polysomnography is the gold standard in SD evaluation; however, its cost is high and it is difficult to perform, which limits its use in population studies. Sleep diaries are inexpensive but require time to complete and are often difficult to interpret.^343^ Written questionnaires to assess SD have been developed for different age groups, self-administered and easy to apply, bear low cost and useful for use in large studies.^344^, ^345^

Questionnaire with seven domains (resistance to sleep, sleep duration, anxiety about sleeping, night waking, parasomnia, change in breathing during sleep and daytime sleepiness) was designed to check sleep habits in children (CSHQ, Children's Sleep Habits Questionnaire),^346^ validated for Portuguese and Spanish.^347^ This instrument was applied to children with asthma and/or allergic rhinitis in nine Latin American countries, including Brazil. A significantly higher CSHQ score was observed in children with asthma and/or rhinitis compared to healthy children, especially in those in whom the disease was poorly controlled.^348^

The same instrument was applied to children with AR of different intensities and greater sleep disturbances were documented among those with more severe forms.^349^

Other instruments have been applied to assess sleep disorders in patients with allergic rhinitis: Sleep Disorders Scale in Children (SDSC),^350^, ^351^ Pittsburgh Sleep Quality Index (PSQI) and Epworth Sleepiness Scale (ESS), both validated for the adult population.^352^, ^353^, ^354^

Among the instruments validated for Portuguese, the SDSC proved to be the most comprehensive in children under 18 years of age.^352^ It consists of 26 items distributed in six subscales: (a) Sleep initiation and maintenance disorders; (b) Sleep-disordered breathing; (c) Awakening disorders; (d) Sleep-wake transition disorders; (e) Excessive daytime sleepiness; and (e) Sleep hyperhidrosis. This scale was applied to patients with isolated allergic rhinitis and mixed rhinitis (allergic + non-allergic). Among the latter, there were more important values in relation to the total score and the subscales compared to those with isolated AR, reflecting greater severity of the former.

## Comorbidities

### Rhinoconjunctivitis

Rhinoconjunctivitis is characterized by simultaneous inflammation of the nasal and conjunctival mucosa. It is a prevalent condition that significantly affects an individual’s quality of life. The causes of rhinoconjunctivitis are diverse, and can be allergic (the most usual form, triggered by allergens such as pollen, dust mites and animal dander), viral (rhinovirus, adenovirus, influenzae virus), bacterial (rare, the most common agent is Staphylococcus aureus) and irritative (irritating agents such as smoke, chemicals and chlorine).

The most common cases are linked to allergic conjunctivitis. Brazilian data report a prevalence of rhinoconjunctivitis of 15%–28%.^355^ Up to 44% of asthmatic children under 14 years of age report at least one ocular symptom, although only a third of them have a medical diagnosis of allergic conjunctivitis.^356^ We can basically classify ocular allergy in 5 types:^357^

1 Seasonal and Perennial Allergic Conjunctivitis (SAC, PAC): SAC is the most prevalent form of ocular allergy, affecting 22% of the population. Its symptoms appear seasonally and last less than four weeks. PAC is characterized by signs and symptoms that persist for days, a week and for more than four consecutive weeks. Patients present with pruritus, hyperemia, papillary conjunctival reaction, tearing and eyelid edema. Chemosis and serous and mucous discharge may be present.
2 Atopic keratoconjunctivitis: It is severe and chronic, and it mainly affects men in the third to fifth decades of life. It is associated with atopic dermatitis in almost 100% of cases. Common signs are gelatinous hyperplasia of the limbus (Horner–Trantas nodules) and papillary hypertrophy, in the inferior tarsal conjunctiva. There may be complications such as eyelid changes and corneal opacities.
3 Vernal keratoconjunctivitis: Rare and severe form of ocular allergy that occurs in the first decade of life in approximately 80% of the patients, with a predominance of males. It is self-limiting, with significant improvement/cure until puberty. Typical findings include giant papillae, limbal Horner-Trantas nodules, and shield ulcers.
4 Giant papillary conjunctivitis: Is induced by mechanical irritation from contact lenses, ocular prostheses or ocular sutures.
5 Allergic contact conjunctivitis: Occurs after sensitization of the eye with some substances.

SAC and PAC are the forms most commonly linked to rhinoconjunctivitis and represent more than 90% of all cases of allergic conjunctivitis. Allergic rhinoconjunctivitis is caused by an IgE antibody-mediated hypersensitivity reaction, provoked by aeroallergens in most cases.^358^ The pathognomonic sign is pruritus. Treatment consists of non-pharmacological measures that seek to prevent or minimize contact between the allergen and the conjunctiva. Topical pharmacological treatment is indicated, starting with antihistamine eye drops or cell membrane stabilizing agents (mast cells), multiple action medications, non-steroidal anti-inflammatory drugs and corticosteroids. Immunotherapy can be used to suppress or regulate the response and slow the allergy progression.^359^ In summary, allergic rhinoconjunctivitis represents a common disease pattern that can be treated effectively. Once correctly diagnosed, targeted treatment quickly results in improved quality of life for patients.

### Mouth breather and apnea syndrome, obstructive sleep hypopnea

There is a consensus that rhinitis has a significant association with sleep disorders. Once again, this concept, widespread among healthcare professionals, is based on prevalence studies where the population with rhinitis has more sleep-related problems than individuals without this type of disorder.^353^ This correlation is also supported by the reasoning that the nose would be one of the main causes of cranio-facial changes, therefore causing mouth breathing and its obvious consequences in several disorders including Obstructive Sleep Apnea and Hypopnea Syndrome (OSAHS).^360^

However, it is especially important to make it clear, as stated in the American consensus, that inadequate growth of the craniomaxillofacial complex is multifactorial, as a result of genetic, functional and environmental factors. In other words, mouth breathing is multifactorial and OSAHS itself has a complex etiopathogenesis.^20^ That is, the nose is important in this context, but with some variations in terms of degree of impact depending on age (children would be more sensitive), it is not a factor alone or a main factor in the genesis of these problems. Although it is not the specific focus of this topic, it is worth noting that, sometimes, the nasal function is overvalued in relation to sleep disorders, especially snoring.

In any case, this warning does not change the fact that rhinitis is undoubtedly one of the causes of nasal obstruction and unsatisfactory airflow related, at the very least, to the worsening of sleep disorders as well as making it difficult to treat appropriately. Therefore, the correct diagnosis of rhinitis and therapeutic control is important in patients with OSAHS and other sleep disorders.^19^, ^20^ The literature since the last publication does not present any innovative findings in this context. Systematic reviews, always overly critical in relation to the quality of available studies, reinforce the correlation of AR not only with OSAHS, but also with other disorders such as: greater use of sleeping medications, insomnia, nocturnal enuresis and snoring. There are gaps in the search for other relationships between these nasal inflammatory disorders, atopic or not, and sleep disorders that do not exclusively worsen nasal function.

It is important to insist on guidance for the investigation of rhinitis among patients with sleep disorders, as well as always diagnosing and treating these problems, both because of the important impact on quality of life per se, and because of the possibility of minimizing and/or preventing worsening of nocturnal respiratory issues determined by other, more well-established factors, such as craniomaxillofacial changes, neuromuscular problems, genetic issues, among others.^361^

### Otitis media with discharge

Human hearing depends on the complex energy transduction function provided by the outer, middle and inner ears. The fundamental energy enhancement made possible by the middle ear depends on a tympanic cavity continuously filled with air and a balance of gaseous components physiologically maintained between the absorption of the mucoperiosteal lining of the Middle Ear (ME) and mastoid and its greater replacement through the auditory tube. Therefore, tubal dysfunction is one of the main etiological factors related to the development of chronic otitis, especially Otitis Media with Effusion (OME). Within this reasoning, any element that leads to worsening of nasal function is automatically related to poor tubal function and as a factor related to changes in the ME.

Based on this maxim, historically, allergic rhinitis and atopy have been considered as risk factors for the development of OME.^362^, ^363^, ^364^ This view, also proposed in the 2018 consensus, continues without being refuted by the new evidence published in recent years, as follows without assuming a main role in the pathophysiology of OME.^19^, ^364^, ^365^, ^366^, ^367^ There are much more complex issues than adequate tubal function in the genesis of this disease, among which there is still a need for studies understanding the role of inflammatory disorders caused by atopy, not only in the tube itself, as in the context of the function of the respiratory epithelium, gas exchange and mucus type. Only then there will be room to believe that the management of allergic processes alone can resolve the accumulation of fluid in the middle ear.

On the other hand, it is important to emphasize that the diagnosis of atopy among these patients continues to be indicated, as well as its appropriate management, due to all other well-defined factors that have a positive impact on the quality of life of these individuals.

### Acute rhinosinusitis

The role of AR in Acute Rhinosinusitis (ARS) is the subject of several studies. Assuming that inflammation of the nasal mucosa resulting from allergic rhinitis is a risk factor for the development of ARS is tempting and seems logical.^368^ Despite this, according to the EPOS-2020, there appears to be little evidence to define AR as a risk factor for the development of ARS.^369^ A systematic review published in 2014 found no evidence that correlated ARS with AR.^370^ In 2020, another systematic review in children correlated AR with a greater predisposition to viral infections in the airway, such as ARS.^371^

Allergy can cause inflammation of the nasal mucosa, impairing the function of the epithelial barrier as well as mucociliary clearance and, in addition, secondary impairment of the local immune response. The mucosa not only has a mechanical barrier function, but also an immunological barrier, modulating the innate immune response through the production of cytokines/alarmins. Furthermore, inflamed mucosa may have increased expression of Intercellular Adhesion Molecules 1 (ICAM-1), which is a receptor for 90% of rhinoviruses, and deficiency in the production of interferon, which has an apoptosis effect on infected cells and promotes phagocytosis of these infected cells.^371^

Fasce and colleagues demonstrated that children treated with cetirizine had a significant reduction in ICAM-1 expression in epithelial cells, thus preventing relapse of rhinovirus infections and decreasing the number and severity of recurrent respiratory infections in children.^372^

Barberi and colleagues demonstrated that children treated with sublingual immunotherapy had significantly fewer respiratory infections than children treated for their symptoms alone.^373^

According to the International consensus statement on allergy and rhinology: rhinosinusitis 2021, AR contributes to ARS, bearing a level of evidence C.^374^

One caveat is that these two diseases can coexist in the patient at the same time and, to confuse the diagnosis, some symptoms overlap (e.g., nasal congestion, hyposmia, pressure on the face).^374^

### Chronic rhinosinusitis (CRS)

The role of AR in CRS as a whole ‒ both in CRS without Nasal Polyps (CRSwoNP) and in CRS with Nasal Polyps (CRSwNP) ‒ is controversial.^375^ According to the International Consensus statement on Allergy and Rhinology: allergic rhinitis 2023 (ICAR-2023), the level of evidence for the association of AR and CRSwNP and AR with CRSwoONP is D.^20^

However, recent studies that used CRS subclassification criteria into phenotypes/endotype point to the CRSwNP phenotypes associated with the type 2 inflammatory response for the possibility of association with IgE-mediated allergy. Examples of this are Allergic Fungal Rhinosinusitis (AFRS) and Central Compartment Atopic Disease (CCAD).^369^, ^376^ For these subtypes, ICAR-2023 classifies the level of evidence as grade C.^20^

Since AR is also characterized by type 2 inflammation and this resembles eosinophilic CRSwNP, a comparison between the diseases is inevitable. However, despite this overlap of immunological pathways, the mechanisms by which allergy may influence CRS are not completely clear.^377^

### Central Compartment Atopic Disease (CCAD)

CCAD is a more recently described variant of CRS and is strongly associated with allergy.^20^ First described by White and colleagues in 2014 (at that time it was not yet known as CCAD, this variant includes polypoid degenerations of the middle turbinate.^378^ An etiological justification was that allergens adhered to the anterior surface of the middle turbinate during the respiratory process. In this study, with only 25 patients, all tested positive for aeroallergens. Such data were confirmed by Hamizan and collaborators in a subsequent study with a larger number of patients.^379^

Brunner and colleagues documented a greater association of sensitization to allergens in patients with isolated changes of the middle turbinate than in those with diffuse polyposis.^380^ Subsequently, DelGaudio, in 2017, published an additional clinical description of this disease, including more advanced forms demonstrating that other central structures, including the posterosuperior nasal septum, middle turbinates and superior turbinates, are involved.^381^ In this study, almost all patients demonstrated sensitization to inhalable allergens and the term: “central compartment atopic disease” was introduced.^374^

A study of radiological findings associated with CCAD documented that a central pattern of mucosal disease had the greatest association with allergy.^382^ Overall, this central pattern of inflammatory changes was shown to have a high association with allergy.

### Allergic fungal rhinosinusitis

Fungi are present in all nasal passages of healthy individuals without causing disease. In immunocompetent individuals, they can become pathogenic when mucociliary clearance is compromised, allowing them to multiply and form a fungal ball.^369^

The immune response to this fungal ball is similar to the foreign body reaction in the affected paranasal sinus (no specific IgE involved). However, another situation is possible in predisposed individuals: a hypersensitivity immune response may occur. Thus, fungi can induce a strong Th2 immune response and, consequently, lead to a polypoid inflammatory phenotype, known as allergic fungal rhinosinusitis (AFRS).^369^

AFRS is a non-invasive subtype of CRSwNP that is associated with specific IgE hypersensitivity to fungi.^383^ The most accepted criteria are the 5 elements established by Bent and Kuhn:^384^ (1) Presence of nasal polyps, (2) IgE-mediated hypersensitivity to fungi (skin or serum test), (3) Eosinophilic mucin (commonly there is intense eosinophilic degranulation with formation of Charcot-Leyden crystals), (4) Presence of non-invasive fungal structures and (5) radiological changes characteristic of the presence of fungi, such as compact hyperdensities in the paranasal sinuses.^376^

Therefore, according to the criteria mentioned above, all patients with AFRS are allergic to fungi.^383^ Atopy is a pre-defining condition and allergic diseases such as allergic rhinitis are quite common in this group.^369^ However, allergic rhinitis to fungi should not be confused with AFRS.^384^

### Adenoid hypertrophy and allergic rhinitis

Pharyngeal tonsil (adenoid) hypertrophy is considered pathological when it causes symptoms of nasopharyngeal obstruction.^385^ Because adenoids naturally shrink during adolescence, children aged 1–6 years are most commonly affected by adenoid diseases.^20^, ^385^

The causes of adenoid hypertrophy in children are not fully known.^386^ They are associated with immunological reactions, hormonal and genetic factors.^387^ Gastroesophageal reflux in newborns and younger children may contribute to adenoid hypertrophy as well as passive smoking.^387^, ^388^

The question that has been asked for a long time is: can allergies cause adenoid hypertrophy? Many studies evaluated the association between adenotonsillar hypertrophy and allergy, but often reached inconclusive results.^389^

In a systematic review, De Corso and collaborators found some flaws in previous studies, such as failure to differentiate adenotonsillar hypertrophy from recurrent tonsillitis, failure to discriminate adenotonsillar hypertrophy from isolated adenoid hypertrophy and isolated tonsillar hypertrophy.^389^ Correcting these flaws, this review systematically confirmed a link between allergy and adenotonsillar hypertrophy and isolated adenoid hypertrophy; while studies have described a mainly negative correlation between allergy and isolated tonsillar hypertrophy.

According to ICAR 2023, the correlation between allergic rhinitis and adenoid hypertrophy has grade C evidence.^20^

### Laryngeal disease and allergic rhinitis

AR and inhalant allergy have been associated with laryngeal diseases; however, understanding of its precise role in laryngeal disease is limited.^388^

Allergic laryngitis presents as laryngeal inflammation induced by allergens, manifesting with symptoms of cough, dysphonia, throat clearing, laryngeal edema and globus pharyngeus.^20^, ^388^

A South Korean national cohort study on the risk of laryngeal disease in patients with AR confirmed that AR is a risk factor.^390^

The correlation between AR and laryngitis can be attributed to the following mechanisms: (1) Direct inflammatory reaction in the larynx, (2) Increased mucus production and its passage through the larynx and (3) Secondary edema in the vocal folds.^390^

Allergic laryngitis associated with AA can be difficult to distinguish from other inflammatory diseases such as Laryngopharyngeal Reflux (LPR). Laryngeal findings in LPR and allergic laryngitis may be similar. Laryngeal edema and erythema and excessive thick mucus are often seen. However, thick endolaryngeal mucus may predict allergy.^20^

According to a recently published review article, the authors suggest that allergic sensitization should be considered in the differential diagnosis of patients with symptoms of chronic laryngitis and LPR should not be the only diagnosis suggested.^391^

According to ICAR-2023, the association of AR and allergic laryngitis is of grade Cevidence.^20^

### Asthma (associated with AR and non-allergic rhinitis)

The concept that the upper and lower airway are part of the same anatomical and functional unit is consolidated and scientifically supported.^153^

The concomitant presence of rhinitis and asthma diseases and characteristics of this relationship, were documented by the Allergic Rhinitis and Its Impact on Asthma (ARIA) initiative in an international guideline published for the first time in 2001.^153^ Among other objectives, the ARIA Guideline reinforces the concept of united airway and proposes the dissemination of this knowledge among generalists and specialists, in addition to a standardized therapeutic approach to rhinitis.^153^

More than 20 years after this hypothesis, the same group has recently discussed that rhinitis associated with asthma is different from rhinitis alone and postulated it as the “ARIA-MeDALL Hypothesis”. The “Epithelial Barrier Hypothesis” is a recent concept that proposes that diseases of the upper and lower airway, allergic skin, intestinal and neuropsychiatric diseases are multimorbidity that go together. This has been demonstrated by (a) Clinical observations that led to ARIA; b) New insights into polysensitization and multimorbidity; (c) Advances in mHealth for new definitions of phenotypes; (d) Confirmation in epidemiological studies; (e) Genomic discoveries, (f) Treatment approaches and g) New concepts about the emergence of rhinitis and multimorbidity. This hypothesis has defined new phenotypes, which include an extreme “allergic” phenotype (asthma) that combines asthma, rhinitis and conjunctivitis. Isolated rhinitis and the multimorbidity of rhinitis and asthma represent two distinct diseases with the following differences: (a) Genomic and transcriptomic background (Toll-Like receptors and IL-17 only for rhinitis as a local disease; IL-33 and IL-5 for allergic and non-allergic multimorbidity as a systemic disease), (b) Patterns of sensitization to allergens (mono- or pauci-sensitization vs. polysensitization), (c) Severity of symptoms and (d) Response to treatment.^392^

### Atopic dermatitis

The natural course of atopic diseases can be characterized by a progressive sequence of clinical signs that can begin in the first months of life with Atopic Dermatitis (AD), food allergy and eventually encompass the airway with asthma and AR, with a predominance of some symptoms more than others, the so-called atopic march.^393^

AD can be defined as a common chronic inflammatory skin disease, with multifactorial etiology, which manifests clinically with intense itching and recurrent eczematous lesions, xerosis and lichenification. It begins in children under two years of age, with a family history of atopy and is often the beginning of the atopic march.^394^ Most of the time, its diagnosis is clinical and with criteria that are often little used in clinical practice, such as the Hanifin & Rajka criteria. 1980 and 1994 UK working group diagnostic criteria, among others.^395^, ^396^

In its most severe and early-onset forms, AD can persist and reach adults, and it is often associated with food allergies, asthma and allergic rhinitis.^397^, ^398^

Many patients improve from AD and become asymptomatic in adolescence when AR already predominates, reaching prevalences of up to 60% of atopic adults followed after 15 years by Mortz and colleagues.^399^

In general, even with population and age group variations in diverse types of studies, the latest international AR guideline concluded, based on prospective, cross-sectional, population-based and observational studies, that there is an association between AR and AD with risk 2–4 times higher AR in patients with AD.^20^

### Food allergy

Suspected Food Allergy (FA) is a common situation in children, and it can be triggered by IgE-mediated or non-IgE-mediated mechanisms. In IgE-mediated cases, the history may often be suggestive, but the presence of IgE to the allergens is negative, or vice versa. In these cases, oral challenge with the suspected food(s) is necessary for the adequate diagnosis of FA, thus avoiding the exclusion of foods that may be nutritionally important for children.^400^

The presence of early allergic sensitization, before two years of age, has been related to the development of several allergic diseases, including AR in children. More recently, it has been observed in cohort studies that the finding of food sensitization associated with aeroallergens shows a greater risk in the development of asthma and AR.^401^, ^402^, ^403^, ^404^

Many cross-sectional and even longitudinal studies point to what is called the “atopic march”, which shows the appearance of new allergies throughout the child’s years of life. In general, this process begins with AD and/or FA and gradually develops into Asthma (A), AR and more recently, eosinophilic esophagitis has been incorporated into this evolution. The sequence does not always occur this way, there may be different initial diseases and evolutions, some allergic diseases gradually resolving or becoming less symptomatic and AR tending to be the symptomatic allergy that most predominates after adolescence.^405^

The ancient practice of excluding allergenic foods (cow's milk, eggs, peanuts and fish) in pregnant women, nursing mothers and even children at risk has not been shown to be beneficial in the development of allergies. It is currently believed that the early introduction of certain allergenic foods (peanuts, eggs, milk) may even reduce the progression to FA and other allergic diseases in childhood.^393^, ^406^

Family genetics, alone, is not capable of explaining the atopic march, and epigenetics, interference by environmental factors and the microbiota have been shown to be important in this evolution. The concomitant presence of AD and FA seems to be more related to the evolution of the atopic march to the respiratory tract with the skin barrier breakdown, the increase in alarmins (TSLP, IL-25 and IL-33), reduction of filaggrins and changes in the microbiome.^393^, ^407^ In the case of AR, older children and adolescents may often no longer express the AD or FA of early childhood.^393^, ^407^

Regardless of the atopic progression, we must remember that some foods can trigger symptoms directly in the oral and nasal mucosae, such as what occurs in Oral Allergy Syndrome (OAS) or pollen-food allergic syndrome. This syndrome begins with the production of IgE specific to pollens, which cross-react with several homologous proteins present in some fruits, vegetables and nuts. The best-known pollen-fruit cross-reaction is related to the presence of the major birch pollen allergen (Bet v 1) and the apple allergen (Mal d 1). Its prevalence varies depending on the geographic region of countries with a predominance of pollination, being between 4% and 20% among children, reaching up to 58% among adults in these areas. Anaphylactic manifestations are rarer, but can reach 2%–10%.^408^, ^409^ Although uncommon in Brazil, this reaction should be remembered when transient symptoms of oral and/or nasal itching, local angioedema and rarely systemic symptoms occur not only with fruits, but also with vegetables, legumes and nuts (carrots, celery, soybeans, peanuts, bananas, tomatoes, zucchini, among others) in patients living in pollinosis areas. In Brazil, with an almost exclusive predominance of sensitization to grass pollens, with little presence or absence of exotic trees with allergenic potential, the risk of OAS is lower than where birch is common. Questionnaire assessment among allergists in southern Brazil identified OAS that was repeated in diverse ways, on 57 occasions, among those studied. The top five foods responsible were: watermelon 9 (39%), banana and peach 6 (26%), pineapple 5 (22%), and avocado 4 (17%). Here 2 (9%) are associated with bee honey, related to the probable presence of pollen.

The diagnosis of OAS requires a typical and repetitive history, aided by the presence of specific IgE to pollens and fruits/foods (often using the “prick-to-prick” technique (due to the lack of allergenic extracts available to perform the prick test) and in some cases by oral provocation test. Cooking can change the conformation of several food allergens that no longer cause symptoms and can make diagnosis difficult if not used in their natural form. More recently, the search for specific components (CRD ‒ Component-Resolved Diagnostics) may be useful in diagnosing cross-reaction between these allergens.

The exclusion of food, greater care and intensification of AR treatment in the pollination season are indicated. Pollen immunotherapy can help reduce symptoms in specific cases but is not routinely recommended.^408^, ^409^

Cross-sectional and cohort studies show the association between FA and the progression to AR in atopic progress, especially with allergies to peanuts and fish, the persistence of which is more common.^410^, ^411^ IgE-mediated FA to various foods may present, within its clinical spectrum, symptoms of AR. The most common symptoms in FA include skin manifestations (acute urticaria, erythema, angioedema), gastrointestinal manifestations (diarrhea, nausea, vomiting, abdominal pain, oral itching), airway (runny nose, tearing, nasal congestion, ocular congestion, nasal/ocular itching, sneezing, wheezing, coughing, stridor, hoarseness) and more rarely, and associated with anaphylaxis, cardiovascular and neurological changes (hypotension, vertigo, syncope, tachycardia). Signs and symptoms appear within minutes to a few hours and FA is rarely manifested solely and exclusively by nasal or lower airway symptoms.^410^, ^411^

### Cough as a symptom of rhinitis

It is still debated whether rhinitis can be the cause, in isolation, of chronic cough in adult and pediatric patients.^412^ Epidemiological data are even more scarce, especially due to the difficulty in demonstrating rhinitis as a cause of cough in individuals where asthma may be a reality.^412^, ^413^

Data collected by the International Study of Asthma and Allergies in Childhood (ISAAC) showed the association between rhinitis and night cough, which was even more significant in children who did not have asthma (adjusted OR [95% CI]: 3.65 [3.36–3.97] in children aged 6–7 years, 3.05 [2.79–3.32] in those aged 13–14 years and 2.69 [2.51–2.88] in those aged 16–17 years), demonstrating a close association between rhinitis and nocturnal cough in young children and adolescents, and this effect was independent of asthma.^413^

The hypothesis, previously suggested, to explain the relationship between cough and rhinitis, through direct stimulation of the nasal mucosa, has been abandoned, since studies have shown that sensory activation directly to the nasal mucosa was not capable of initiating cough.^412^, ^414^ It should be noted that other mechanisms, other than local stimulation itself, such as post-nasal drip, microaspiration of inflammatory aerosol and deficits in nasal functions with cold and dry inhalation, may be involved in the genesis of cough in rhinitis.^412^, ^413^

The currently most accepted mechanism is the sensitization of the cough reflex, as an up-regulation of this reflex, where its activation threshold would be reduced and less intense stimuli would be sufficient to cause or intensify the cough.^415^, ^416^

The cough reflex sensitivity threshold is measured by inhalations of increasing concentrations of an aerosolized irritant, commonly capsaicin or acidic solutions,^414^, ^416^ and defined as the lowest concentration of the irritant required to elicit a predetermined number of coughs (2 and 5 episodes, C2–C5).^416^

Cough reflex sensitivity to inhaled capsaicin is increased in adult AR patients with normal lung function and increased further during the pollen season in pollen-sensitive patients^417^ and also in response to inhalation of warm humidified air.^418^

**Fig. 4:**
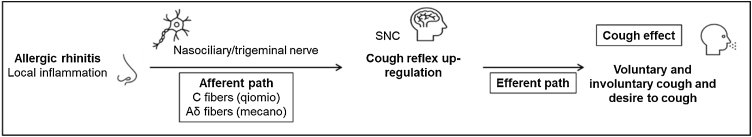
Simplified schematic representation of the coughing mechanism in allergic rhinitis.^415^

Repeated exposure to the allergen induces inflammation of the nasal mucosa, with the release of inflammatory mediators, which stimulate the neural pathway at multiple levels. It is not known exactly which afferent nerves mediate the sensitization of the cough reflex from the nose. It is believed that the cell bodies of these nerves are the primary afferent neurons located in the trigeminal ganglia and projecting to the nasal mucosa. This afferent pathway would be represented by C fibers (chemoreceptors, which express TRPV1) sensitive to capsaicin, histamine, and Aδ (mechanoreceptors), starting a circuit that will involve the CNS and the efferent pathway, culminating in the “coughing effect”, characterized by voluntary and involuntary coughing and the urge to cough (Fig. 4).^412^, ^415^, ^416^

This “neuroimmune communication” and its involvement in the pathogenesis of cough in RA has been highlighted in the literature. The idea would be that the development of nasal hyperresponsiveness is linked to the modulation and reflexes of immune cells, where the formation of “axonal reflexes” and “central sensitization” would promote the development of “neurogenic inflammation”. In the last decade, at the basic science level, cough research has been dedicated to studying “cough plasticity”, that is, the modulation of the reflex response at both peripheral and central levels.^412^, ^415^, ^416^, ^419^^,^^420^

With this knowledge clarified, studies have shown that treatment of nasal inflammation with local corticosteroids, cis-LT1 leukotriene receptor antagonists or oral rutinoscorbin significantly decreased the magnitude of nasal symptoms, leading to desensitization of the cough reflex to pre-illness values.^412^, ^415^, ^416^, ^419^^,^^420^

In practice, success in treating coughs associated with rhinitis would be achieved by “desensitizing” the cough reflex by controlling local inflammation. Local corticosteroids, antihistamines or cis-LT1 leukotriene receptor antagonists act on most nasal symptoms, reducing secretion and the extent of nasal obstruction, therefore influencing all the mechanisms discussed (Fig. 4).^412^, ^415^, ^416^, ^419^^,^^420^

## Treatment

### Non-pharmacological treatment

#### Exposure avoidance and environmental hygiene

AR affects approximately 400 million people worldwide and is a worldwide health concern.^421^ The development of AR in people with atopic tendencies is strongly influenced by their exposure to various internal and external environmental factors.^422^ Hence the concern about establishing effective measures to control environmental factors, especially within the home.

#### House dust mites and their allergens

The ubiquity of house dust mites and their allergens in dust particles on beds, carpets and upholstery highlights them as the most important source of household allergens. The most common mite species in Brazilian homes are: Dermatophagoides farinae, Dermatophagoides pteronyssinus and Blomia tropicalis.^423^ In addition to the predominance of HDM, there is a high prevalence of mite sensitization on a global basis.^424^

Like cockroach allergens, and unlike pet allergens, HDM allergens are carried in large particles that deposit quickly on surfaces (after 15 min), and therefore the use of air purifiers has limited effects on their control.^425^

HDM produce and excrete numerous allergens into the environment, including cysteine proteases such as Der p 1, Der p 2 and serine proteases such as Der p 3, 6 and 9. There is cross-reactivity between allergens from various dust mite species (Dp and Df), and because mites are members of the arthropod family, they contain tropomyosin (Der p 10), which is cross-reactive with other arthropods, such as crustaceans and cockroaches.^422^

The mechanisms by which HDM allergens can provoke allergic and inflammatory reactions or even facilitate sensitization to other allergens include the rupture of epithelial tight junctions, the activation of the innate and adaptive immune system and the promotion of a Th2 response.^426^, ^427^

Reducing the concentration of HDM and its allergens is the first line of measures that must be implemented, and the focus must be directed to mattresses and bedding. Mattresses are considered the largest reservoir of HDM allergens, as they are the place where individuals spend most of their time.^428^ Waterproof covers for mattresses and pillows serve to keep dust mites and their allergens away from contact with the airway. The best dust mite covers are made of fabrics with pores small enough to prevent the passage of HDM and its allergens (<10 microns), but which allow the passage of steam, providing greater comfort to the user. Covers with pores <6 microns are also capable of blocking the passage of cat allergens, Fel d 1.^429^

Washing bedding weekly in hot water removes new allergens and is acaricidal. Warm water already removes most HDM allergens, without the risk of burns.^430^ An effective acaricide strategy is drying clothes in a dryer.^431^

HDMs survive in humid environments as they are a source of water for them and reducing humidity to levels of 45% or less drastically reduces mite proliferation.^432^ If indoor humidity increases for at least 1.5 h per day, such as occurs during cooking or bathing, dust mites can survive. If high humidity in the home persists for 3 h/day, dust mites can lay their eggs in the environment. To determine indoor humidity, individuals must have a hygrometer in the room.^422^

The second line of interventions includes work on carpets and upholstery. Weekly vacuuming is essential in preventing the accumulation of HDM and other aeroallergens. Vacuuming does not remove all live HDM, but it does remove its allergens in the form of fecal particles.^433^

The use of acaricides and tannic acid has limited effects and is difficult to use. Miticides kill surface mites when applied to carpets, mattresses and upholstery surfaces. However, apart from a modest drop in the total amount of HDM allergens, the effects last for a maximum of three months.^422^

Physical measures such as freezing, heating and desiccation are only theoretical, without scientific proof.

#### Pets (furry pets)

The most common pets are cats and dogs, although the presence of hamsters, pigs, cockatiels, among others, is observed in some homes. Domestic animal allergens are carried into the environment in small particles and most remain suspended in the air, even with little movement in the environment. The main dog and cat allergens, Can f 1 and Fel d 1, respectively, in developed countries, are ubiquitous in homes and public environments, most likely due to the passive transfer of the clothing of the person in contact with the animal to other environments.^434^ Therefore, allergy to these proteins is considered a public health problem.^435^

Fel d 1 is produced in the sebaceous, anal and salivary glands and transferred to the hair by animal's licks.^436^ While Fel d 1 suspended in the air is mainly associated with large particles (>9 μm), around 23% of the suspended portion it is carried in small particles (<4.7 μm diameter) that remain in the air for days.^437^ In any case, homes where there is a domestic animal have much higher levels of allergens than homes without pets. There is great variability in Can f 1 levels according to the breed of the animal, but there is no evidence that a hypoallergenic dog breed exists.^438^

Among environmental interventions to control domestic animal allergens, the first recommendation would be not to keep the animal in the patient's home. This attitude significantly reduces the concentration of allergens, and it is associated with a drop in airway hyperresponsiveness, with a reduction in the dose of inhaled corticosteroid for control.^439^ The substantial reduction in the levels of pet allergens in the environment can take up to six months, for example: ability of the allergen in question to remain in suspension and also adhere to surfaces. The doctor must be sensitive when proposing the separation of the animal as this is often impossible. Therefore, other recommendations (with less effectiveness) follow, such as keeping the animal outside the patient's bedroom and the use of high-power air purifiers with HEPA filters.^425^ It is necessary to ensure that the purifier used does not cause the movement of particles already settled on surfaces, into the environment.^440^

The relationship between exposure to cat allergens and allergic sensitization is complex. Some variables in this case are related to the allergen itself, such as its biological activity, the moment of contact and the duration and intensity of exposure.^441^ A recent study found a connection between exposure to pet allergens in children and epigenetic modifications, with a lower probability of developing AR in preschool children.^442^ The main explanation regarding allergy prevention in children exposed to dogs and cats in the first years of life is that early exposure to large quantities of aeroallergens could train the immune system to become tolerant (to the allergen in question).^443^

#### Cockroaches

Germanic and American cockroaches are common in urban areas and produce the main allergens Bla g 1, Bla g 2 and Per a 1, respectively.

Measures in the integrated management of these insects include closing drains and gaps, eliminating sources of water and food and applying baits and products licensed for use.^444^

#### Fungi

The fungi most detected in homes are: *Cladosporium* spp, *Penicillium* spp, *Aspergillus* spp and some species of Alternaria spp.^445^ The genera Alternaria and Cladosporium are also found in external environments. Sensitization and exposure to fungi are associated with the development of asthma and rhinitis, as well as “epidemics” of asthma attacks.^446^

Measures to control fungi in homes involve good ventilation, sunny environments, cleaning visible fungi with chlorine and controlling indoor humidity. This control can be done with air purifiers with a filter.^446^

#### Pollutants

Climate change and pollutants can alter plant physiology, resulting in longer pollination duration and more allergenic pollens.^447^ These factors can increase patient sensitization and symptoms in allergic patients.

The most common air pollutants in urban areas are Nitrogen dioxide (NO_2_), Ozone (O_3_) and Particulate Matter (PM, while Sulfur dioxide (SO_2_) originates from industrial activities and traffic energy sources (combustion of coal and oil).

It is estimated that diesel exhaust represents up to 80% of the material to which humans are exposed.^448^ Exposure to environmental elements, considered as “the exposome”, greatly affects the balance of mucous membranes.^449^ Air pollution, particulate materials from Diesel Exhaust (DEP), related air pollutants (TRAPs), O_3_, NO_2_ and SO_2_ can cause irritation to the mucous membranes, increased mucosal permeability (barrier breakdown) and impaired mucociliary transport, facilitating the penetration of allergens and greater interaction with immune system cells.^450^

Individuals with AR exhibit nasal hyperreactivity, making them more responsive to inhaled irritants. Smoke generates a lot of Particulate Matter (PM) in the environment, as do gas stoves, which are the biggest sources of NO_2_ within the home.

Interventions in homes to reduce PMs include smoking cessation guidelines. When this is not possible, we advise you to use air purifiers with a HEPA filter. Reductions in NO_2_ can be achieved by placing gas ovens and stoves outdoors or switching to electric stoves.

Many controlled studies show the importance of environmental control of aeroallergens in previously sensitized individuals with asthma and/or rhinitis. There is evidence of lower exposure to allergens when multifaceted methods are used with a combination of techniques in environmental control. These techniques involve repetitive and sequential interventions and should be recommended for patients sensitized to HDM, with allergic manifestations. Combined interventions to reduce HDM allergens from environments include maintaining indoor humidity at 35%–50%, weekly washing of bedding to remove dust mites and allergens, regular vacuuming (HEPA filters), use of special covers in mattresses and pillows and air purifiers (HEPA filters), when necessary.^422^

#### Nasal Wash

Nasal lavage with saline solution has been used empirically for centuries to treat various nasal and sinus conditions. As it is a cheap, practical and well-tolerated method, it has become widespread, and in recent years there has been growing interest in studying its mechanisms of action. The main and clearest thing is cleaning the nasal cavities, facilitating the removal of pathological secretions and consequently promoting symptomatic relief to patients.^369^

In inflammatory and allergic rhinitis, nasal washing also promotes the removal of inflammatory mediators present in the nasal mucus and allergens present in the nasal cavity, thus reducing the allergic stimulus.^369^ The regular use of nasal saline solutions improves nasal symptoms, the duration of mucociliary clearance and the quality of life of patients with allergic rhinitis.^20^

Saline solutions for nasal use differ from each other in relation to osmolarity, and can be hypotonic (<0.9%), isotonic (0.9%), or hypertonic (>0.9%, the most used being those at 2% and 3%). Furthermore, the pH can be adjusted, ideally it should remain neutral or slightly alkaline (between 7 and 9). Saline solution (water and sodium chloride only, at 0.9%) has a pH of around 6, that is, slightly acidic.^20^, ^369^, ^374^

In general, the most widely used solution is isotonic, which guarantees a good cleaning, humidifying effect and a certain decongestant effect on the mucosa, being the most comfortable to use, as it does not cause nasal burning. Nasal lavage with hypertonic solution also causes an effect of increasing mucociliary clearance and some studies claim that this effect is superior to that of isotonic solution, but it is uncertain whether this in vitro superiority translates into clinical superiority. Hypertonic solutions also have a superior effect on mucosal humidification, as they cause the transport of water molecules from the cells of the nasal mucosa from the intracellular to the extracellular environment. For the same reason, the decongestant effect is more intense by reducing mucosal edema. On the other hand, use in patients without edema, and/or for a prolonged period of time, can cause dehydration and mucosal irritation. Therefore, the use of hypertonic solution is usually reserved for patients with intense edema (e.g., acute rhinosinusitis, rhinitis drug) or need for intense humidification (e.g., presence of crusts), and normally for a shorter period of time (days to a few weeks).^451^, ^452^

The major limitation in the use of hypertonic solutions is the patients' discomfort, burning, which becomes more intense as the sodium concentration increases. The age group of the patients is also a crucial factor, as children are generally less compliant with the use of the hypertonic solution. For the treatment of rhinitis, isotonic solutions with a neutral pH or buffered with a slightly alkaline pH have been shown to be better for nasal symptoms and mucociliary function than hypertonic solutions,^451^ especially for long-term use.

Regarding volume, nasal lavage can be low or high volume, with high volume being considered for adults when around 100 mL per nostril is used. To date, there are no randomized, placebo-controlled clinical trials that have compared the effects in the treatment of rhinitis when using spray washing or high-volume devices. Considering that the nasal secretion of patients with rhinitis is usually fluid and easy to eliminate, the most recommended wash is the one that the patient feels comfortable performing, facilitating treatment compliance.

A recent meta-analysis confirmed the benefits of nasal washing in the control of patients with rhinitis, including in association with drug treatment, as the combination of washing and corticosteroid sprays was superior to the isolated use of medication.^453^ Nasal washing with saline solution also plays an important role in prevention, as it can promote a substantial reduction in the use of medications to control the condition, and the improvement in symptoms can be similar to the use of antihistamines.^19^ Therefore, the regular use of nasal saline solutions can contribute to the reduction in episodes of rhinitis, preventing disease recurrence.

In summary, considering that to date there are several studies demonstrating the benefit of using nasal washes in patients with allergic rhinitis, their use is strongly recommended for these patients.^20^, ^451^, ^454^

#### Probiotics

According to the hygiene hypothesis, signals from environmental exposure (rural, farm environment) and microbiota containing more abundant and diverse bacteria would provide intense signals for the development of a regulatory response (tolerance), while the urban environment and/or a “Westernized” microbiota would not induce this type of response, directing the immune balance towards an inflammatory response. Tolerance promotes a healthy immune response, while in its absence an inflammatory profile develops that can lead to inflammatory airway disease, such as allergic rhinitis and asthma.^455^, ^456^

Probiotics induce immunomodulatory effects on gut-associated lymphoid tissue. The gut microbiome and the immune system interact through dendritic cells, regulatory T-cells, bacterial metabolites, and cytokines. Exposure to probiotics induces a Th1 response via IL-12, IFN-γ, with upregulation of Treg cells via IL-10 and TGF-β. Furthermore, the allergy-associated Th2 pathway is suppressed by downregulation of IL-4, tIgE, IgG1, and IgA.^457^

Numerous randomized controlled trials have examined the therapeutic role of probiotic administration for controlling AR symptoms. Several high-quality meta-analyses have been performed with aggregated data from randomized controlled trials. Results in children and adults have been mixed.

Systematic review with meta-analysis evaluated whether the use of probiotics in patients with AR was capable of modifying the Total Nasal Symptoms Score (TNSS), the quality-of-life score (Rhinitis quality of life questionnaire; RQLQ), the eosinophil count in the blood, the levels of specific and total IgE. A total of 2,708 patients were included in 30 randomized trials. The results of the meta-analysis showed significant improvement in RQLQ scores, RQLQ nasal scores and TNSS in the probiotic-treated group. However, there were no differences in serum eosinophil counts, RQLQ ocular scores, TNSS ocular scores, total and specific serum IgE levels.^458^ Thus, the authors conclude that, in comparison with the placebo group, quality of life and symptoms of patients with AR improved significantly in the probiotic group. However, due to limited evidence for study results, research heterogeneity, and differences in research results, more high-quality studies are needed.

Another systematic review with meta-analysis aimed to verify the efficacy and safety of probiotics in AR. Twenty-eight studies were included. The results showed that probiotics significantly alleviated the symptoms of AR, decreased the RQLQ score, increased the proportion of T helper cells, but without differences in serum levels of total and specific IgE.^459^ The authors concluded that supplementation with probiotics appears to be effective in improving AR symptoms and quality of life, but there is great heterogeneity in some results after subgroup analysis and clinicians should be cautious when recommending probiotics in the treatment of AR.

### Pharmacological treatment

#### Antihistamines (Anti-H1)

H1 anti-H1 drugs are considered first-line medications in the treatment of AR.^21^, ^28^, ^275^, ^460^ They interfere with the action of histamine on sensory nerve endings, the parasympathetic reflex stimulation of glandular secretions, vasodilation and the increase in post-capillary permeability, effectively relieving the main symptoms of the immediate phase of AR such as nasal itching, sneezing, rhinorrhea and associated eye symptoms. On the other hand, they have negligible effect on nasal blockage, a characteristic symptom of the late stage of the disease. In addition to these actions, they negatively regulate nasal allergic inflammation by reducing the expression of inflammatory cytokines, adhesion molecules and the activation of epithelial cells, eosinophils, basophils, mast cells and T cells.^461^, ^462^, ^463^

Due to their mechanism of action, they are defined as histamine reverse agonists, shifting the balance of their receptor to an inactive state and, as a consequence, reducing their constitutive activity.^464^

They have been used since the 1940s and numerous controlled clinical studies, meta-analyses and systematic reviews have established their effectiveness in children and adults for relieving the symptoms of AR, whether intermittent/persistent and/or mild/moderate.^327^, ^463^, ^464^, ^465^, ^466^, ^467^, ^468^, ^469^, ^470^, ^471^

H1 anti-H1s can be classified as classic or first generation (sedating) and non-classic, or second, or third generation (non-sedating) based on their passage through the Blood-Brain Barrier (BBB) and consequent activity on the central nervous system.^431^, ^432^, ^433^, ^434^, ^435^, ^436^, ^437^, ^438^, ^439^, ^440^, ^441^, ^442^, ^443^, ^444^, ^445^, ^446^, ^447^, ^448^, ^449^, ^450^, ^451^, ^452^, ^453^, ^454^, ^455^, ^456^, ^457^, ^458^, ^459^, ^460^, ^461^, ^462^, ^463^

Compared to first generation oral H1 anti-H1s (e.g., diphenhydramine, hydroxyzine and chlorpheniramine), the newer compounds have low passage through the BBB with few adverse effects on the central nervous system, high potency and long duration of action. Furthermore, they have a high affinity for H1 receptors, with little or no anticholinergic, antidopaminergic and antiserotonergic effects. The side effects of the first generation of H1 anti-H1s may be even more pronounced in the elderly, where polypharmacy represents an additional problem for their use.^461^, ^462^, ^463^, ^472^, ^473^, ^474^

Considering their excellent safety profile and therapeutic advantages in the treatment of rhinitis, different evidence-based guidelines recommend that new generation H1 anti-H1s should always be prioritized over older compounds, in all age groups.^20^, ^21^, ^275^, ^280^^,^^460^, ^475^, ^476^

In 2021, a regulatory instruction from the Brazilian Ministry of Health approved most new generation H1 anti-H1s as over-the-counter formulations, making them available without a prescription. This change could promote a reduction in the cost of these medications for patients and improve access as a treatment option for AR.^477^

The doses of the main second-generation H1 anti-H1s used in the treatment of AR in adults and children and their clinical and pharmacological characteristics are described respectively in Table 5, Table 6.

**Table 5:** Main second-generation antihistamines for oral use.

Anti-H1	Description	Dosage and administration	
		Children	Adults
Cetirizine	Oral solution 1 mg/mL	2–6 years: 2.0 mg/12 h	>12 years: 10 mg/day
	Drops 10 mg/mL	6 to 12 years: 5 mg/12 h	
	Pills. 10 mg		
Levocetirizine	Drops 5 mg/mL	2–6 years: 5 drops/12 h	>6 years: 20 drops or 1 pill 1×/day
	Pills. 5 mg		
Loratadine	syrup 1 mg/mL	Older than 2 years	≥30 kg: 10 mg/day
	Pills. 10 mg	<30 kg: 5 mg/day	
Desloratadine	Syrup 0.5 mg/mL	6 months and 2 years: 1 mg 1×/day (2 mL or 16 drops)	6–12 years: 2.5 mg 1×/day (5 mL or 40 drops)
	Drops 1.25 mg/mL		
	Pills. 5 mg		
		2–6 years: 1.25 mg 1×/day (2.5 mL or 20 drops)	>12 years: 5 mg/day (10 mL or 80 drops)
Fexofenadine	Oral suspension 6 mg/mL	6 months to 2 years: 15 mg (2.5 mL)/12 h	6–12 years: 60 mg/day
	Pill. 60, 120 and 180 mg	2–11 years: 30 mg (5 mL)/12 h	>12 years: 120 mg/day
Ebastine	Oral solution 1 mg/mL	2–6 years: 2.5 mg 1×/day	>12 years: 10 mg/day
	Pills 10 mg	6–12 years: 5 mg 1×/day	
Bilastine	Pills. 20 mg	≥12 years: 20 mg/day	1h before or 2 h after meals*
	Oral solution 4 mg/mL	>6 years and >20 Kg: 10mg/day	
Rupatadine	Pill. 10 mg		≥12 years: 10 mg/day

**Table 6:** Clinical profile and pharmacological characteristics of second-generation anti-H1 agents.

Name	Action onset (h)	Action duration (h)	½ life clearance (h)	Interaction with food	Interaction with drugs	Dose adjustment conditions	Contra-indications	Use in pregnancy^c^
Cetirizine	0.7	>24	6.5 + 10	NO	Unlikely	LF, KF	Severe RF	B
Levocetirizine	0.7	>24	7 + 1.5	NO	Unlikely	LF, KF	Severe RF	B
Loratadine	2	>24	7.8	NO	Unlikely	LF, KF	NO	B
Desloratadine	2	>24	27	NO	Potential (CYP3A4, CYP2D6)	LF, KF	NO	C
Fexofenadine	1‒3	>24	11‒15	May happen^a^	May happen^b^	NO	NO	C
Bilastine	1	24	14.5	May happen^a^	May happen^b^	NO	NO	Caution Limited data
Ebastine	2	>24	15‒19	NO	Caution	Caution LF, KF	Severe LF	C
Rupatadine	0.75	>24	6 (4.3–13.0)	NO	N/A	G, LF, KF	G, KF, LF	C

G, Geriatric Population; LF, Liver Failure; KF, Kidney Failure; N/A, Not Available.

a Fexofenadine and bilastine are substrates for P-glycoprotein (Pgp) and concomitant ingestion with some foods that serve as a substrate for Pgp (grapefruit, bitter orange juice) can modify its bioavailability.

b It is recommended to wait a period of approximately 2 h between administrations of fexofenadine hydrochloride and antacids containing aluminum and magnesium hydroxide. The concomitant use of ritonavir or rifampicin can reduce the plasma concentration of bilastine, and rupatadine may interact with ketoconazole and erythromycin.

c Pregnancy risk category according to the North American FDA. Adapted from references.^473^, ^474^

In addition to oral formulations, H1 anti-H1s for topical nasal and ocular use are therapeutic alternatives for the treatment of AR and associated ocular symptoms. Intranasal H1 anti-H1s (INS) have similar efficacy to oral compounds, but have the therapeutic advantage of a faster onset of action (up to 15 min) and greater effectiveness in controlling nasal obstruction, and may be beneficial in some types of non-infectious allergic rhinitis (e.g., drug-induced rhinitis, gustatory).^478^, ^479^, ^480^, ^481^

Most studies show that anti-H1 INS are superior to Intranasal Corticosteroids (INC) in controlling sneezing, itching, rhinorrhea and ocular symptoms, but less effective than the latter in reducing nasal obstruction. The reported adverse effects are mild and uncommon (e.g., drowsiness, headache, epistaxis) occurring in less than 10% of patients treated with azelastine or olopatadine. However, compliance to treatment may be compromised due to the bitter taste and altered taste (dysgeusia).^482^, ^483^, ^484^

The main representatives of this group of anti-H1 are azelastine, olopatadine and levocabastine. We currently do not have isolated intranasal antihistamine formulations on the Brazilian market, only azelastine hydrochloride in combination with fluticasone propionate (Table 7).

**Table 7:** Main second-generation H1 anti-H1s for topical nasal and ocular use.

Anti-H1	Description	Dosage	
		Children	Adults
Nasal topical Spray			
Azelastine^a^	1 mg/mL	≥6 years: 1 squirt in each nostril 12/12 h	1 squirt in each nostril 2/12 h
Levocabastine^a^	0.5 mg/mL	≥6 years: 1 squirt in each nostril 12/12 h	≥12 years: 2 squirts in each nostril 2/12 h
Olopatadine		6–11 years: 1 squirt in each nostril12/12 h	≥12 years: 2 squirts in each nostril 2/12 h
Fluticasone + azelastine association	50 mcg/FLU	≥6 years: 1 squirt in each nostril 12/12 h	1 squirt in each nostril 12/12 h
	137 mcg/AZE/dose		
Topical eye drops			
Ketotifen	0.25 and 0.5 mg/mL	>3 years: 1 drop on each eye 2 to 3×/day (maximum 6 weeks)	1 drop on each eye 2 to 3×/day
Emedastine	0.5 mg/mL	Older than 3 years: 1 drop on each eye 2×/day	1 drop on each eye 2×/day
Olopatadine	1 mg/mL	Older than 3 years: 1 drop on each eye 2×/day	1 drop in each eye 2×/day
Alcaftadine	2.5 mg/mL	≥2 years: 1 drop on each eye 1×/day	1 drop in each eye 1×/day

FLU, Fluticasone; AZE, Azelastine.

a Not available in Brazil.

#### Decongestants

Nasal decongestants are drugs belonging to the group of adrenergic or adrenomimetic stimulants, whose main action is vasoconstriction that produces rapid relief of nasal blockage in AR.^485^ According to the route of application, they are divided into two groups: oral (systemic) and nasal topical (intranasal).

#### Systemic

The most commonly used compounds are pseudoephedrine and phenylephrine. Both are vasoconstrictor sympathomimetics that differ in their selectivity for adrenoceptors. When acting systemically, they can lead to side effects such as insomnia, headache, nervousness, anxiety, tremors, palpitations, urinary retention, increased blood pressure and other adverse effects.^486^, ^487^

Pseudoephedrine belongs to the amphetamine family and should be used with caution due to its psychotropic action and potential cardiovascular side effects. In general, its use is not recommended for patients under four years of age, due to the greater risk of toxicity, and extended-release formulations with doses of 120 mg are not recommended for children under 12 years of age.^485^, ^488^ Study in adults with seasonal AR, the use of different doses of phenylephrine, associated with anti-H1 did not show a decongestant effect superior to placebo.^489^

In summary, although not recommended as a routine medication, pseudoephedrine may be effective in reducing nasal congestion in patients with AR. However, it should only be used as a short-term treatment or as rescue therapy after evaluating the risks and benefits for each patient (comorbidities), or as an alternative for those using intranasal decongestant therapy.^20^

In Brazil, systemic decongestants are only available in combination with first or second generation H1 antihistamines as shown in Table 8, Table 9.

**Table 8:** First generation H1 antihistamines associated with oral decongestants.

Association	Description	Dosage	
		Children	Adults
Azatadine + Pseudoephedrine	Tablets 1 mg azatadine +120 mg pseudoephedrine	‒	1 Pill every 12 h
	Syrup 0.5 mg azatadine +30 mg pseudoephedrine/mL	1‒6 years: 2.5 mL 12/12 h	10–20 mL every 12 h
		>6 years: 5 mL Every 12 h	
Brompheniramine + Phenylephrine	Syrup 5 mL c/2 mg brompheniramine + 5 mg phenylephrine	>2 years: 2.5–5 mL Every 6 h	15– 30 mL every 6 h
	Drops 1 mL c/2 mg brompheniramine + 2.5 mg phenylephrine	>2 years: 2 drops/kg Every 8 h	
	Pills: 12 mg brompheniramine + 15 mg phenylephrine	‒	1 Pill every 12 h
Brompheniramine + Pseudoephedrine	Syrup 1 mL c/2 mg brompheniramine + 3 mg pseudoephedrine	>6 months: 0.–0.30 mL/kg/dose every 6 h	20 mL every 6 h
	Tablets with 4 mg brompheniramine + 60 mg pseudoephedrine	‒	1 tablet Every 6 h
Triprolidine + Pseudoephedrine	Pill: 2.5 mg triprolidine + 60 mg pseudoephedrine	‒	1 pill Every 6 h

**Table 9:** Second generation H1 antihistamines associated with oral decongestants.

Association	Description	Dosage	
		Children	Adults
Cetirizine+ PSE	Tablets 5 mg/120 mg	‒	≥12 years: 1 tablet Every 12 h
Loratadine+PSE	Syrup 1 mg/120 mg/mL	Adults and children >6 years >30 kg: 5 mL Every 12 h.	≥12 years: 1 pill Every 12 h
	Pill 5 mg/120 mg		≥12 years: 1 pill Every 24 h
	Pills. 10 mg/240 mg		
Desloratadine + PSE	Tablet. 2.5 mg/120 mg	‒	≥12 years: 1 tablet Every 12 h
Fexofenadine + PSE	Pills.60 mg/120 mg	‒	≥12 years 1 tablet Every 12 h
Ebastine + PSE	Pills.10 mg/120 mg	‒	≥12 years: 1 pill Every 24 h

PSE, Pseudoephedrine.

#### Intranasal

Intranasal Decongestants (INDCs) such as oxymetazoline, xylometazoline and phenylephrine are α-adrenergic agonists that act as topical vasoconstrictors, reducing edema of the nasal mucosa. When used in the short term, they reduce nasal congestion/blockage, with little or no effect on other AR symptoms. The onset of action occurs within 10 min, and the duration of the effect, depending on the drug, can last up to 12 h.^490^, ^491^

These medications can cause important cardiovascular effects, as well as on the central nervous system, and are contraindicated in children under six years of age. They should also be avoided in the elderly, due to the higher incidence of hypertension and urinary retention with their use in this age group.^485^

Although effective for the short-term relief of nasal congestion in patients with AR during an acute attack, INDCs should be used for a maximum of five days as the risk of rebound medicinal rhinitis, which is often difficult to resolve, increases after longer use.^492^

#### Association of H1 antihistamines and oral decongestants

Antihistamines can be administered in combination with Systemic Decongestants (SD) when nasal obstruction control is not achieved. In general, these combinations are more effective than any oral H1 anti-H1 or SD alone, as they improve nasal congestion and total AR symptom scores.^493^, ^494^, ^495^, ^496^

On the other hand, the addition of a SD to an anti-H1, especially first generation, can cause or amplify side effects such as insomnia, headache, dry mouth and nervousness, hypertension, benign prostatic hypertrophy, and should be avoided in patients younger than 12 years or during pregnancy. Furthermore, it can induce tolerance through its chronic use.^497^

The main combinations of first and second generation H1 antihistamines and oral decongestants are listed in Table 8, Table 9.

#### Ipratropium bromide

It is a synthetic quaternary ammonium derivative related to atropine that has anticholinergic action on trigeminal secretomotor fibers, reducing hypersecretion of the nasal glands. In AR, its main indication is for those patients whose primary symptom is rhinorrhea, as it does not act on the other symptoms of the disease or on the nasal airwayresistance.^498^ Therefore, the IB formulation in intranasal spray can be used as an adjuvant medication to intranasal corticosteroids in AR patients with persistent rhinorrhea.^20^, ^499^ The administration of IB is also capable of reducing rhinorrhea after exposure to cold air and ingestion of hot or spicy foods, and may be indicated for other types of rhinitis, such as rhinitis of the elderly and gustatory rhinitis, where this is the predominant symptom.^38^, ^500^

IB is effective in adults and children with perennial rhinitis and the common cold.^3^, ^4^ It has a rapid onset of action and a short half-life; it can be administered up to six times a day. It has an excellent safety profile, with less than 10% dose absorption; however, it should be avoided or used with caution in patients with prostatic hypertrophy and angle-closure glaucoma.^20^ Currently, presentations for topical nasal use of IB are not available in Brazil.

#### Systemic corticosteroid

Glucocorticosteroids (GCS) are the oldest and most widely used anti-inflammatory therapy. Since their introduction in the 1950s, they have played a key role in the treatment of various diseases, both allergic and immunological.^501^ Years ago, it was not uncommon for seasonal AR to be treated with a short course of oral steroids or injectable depot formulations, but due to adverse events associated with Systemic Corticosteroids (CS), their use was gradually reduced, being proscribed in children and adolescents. Potential adverse effects of prolonged or recurrent use include infections, myopathies, osteoporosis, aseptic necrosis of the femur, thinning of the skin, hyperglycemia, weight gain, fluid retention, cushingoid appearance, neuropsychiatric disorders, cataracts, glaucoma, and hypertension.^502^

Regarding the use of systemic GCS in AR, current evidence is scarce. Hox and colleagues in a systematic review of the literature report on three studies that compared the effect of systemic GCS in patients over 15 years of age with AR and evaluated its efficacy and adverse events.^501^ Borum and colleagues in a randomized clinical trial demonstrated a beneficial effect of a single intramuscular dose of 80 mg Methylprednisolone (MP) compared to placebo for both nasal obstruction and ocular symptoms in 48 patients with AR, lasting four weeks.^503^ Subsequently, Brooks and colleagues investigated the efficacy of different doses of oral MP versus placebo in patients not treated with other medications. Oral GCS produced a reduction in all symptoms, and they were related to the dose used.^504^ Laursen and collaborators compared prednisone 7.5 mg, orally, for three weeks, with a single intramuscular injection of betamethasone dipropionate, both in patients not treated with other medications and demonstrated a therapeutic index in favor of depot corticosteroid versus oral treatment in AR.^505^

Recently, Skröder and collaborators evaluated the role of methylprednisolone in improving the symptoms of pollen-induced AR and the concomitant reduction in the use of medications considered standard in its treatment.^506^ The results demonstrated that the group treated with 80 mg, intramuscularly, in a single dose of Methylprednisolone performed little better than the placebo group, but no significant difference between groups was observed and recorded side effects were few and mild. However, the authors report that the limited beneficial effects of systemic steroids when added to standard treatment, in combination with their potential risk of side effects, speak against their use in the treatment of severe seasonal allergic rhinitis.^506^

In conclusion, Oral Corticosteroids (OC) produce a dose-related reduction in certain AR symptoms. The use of low doses of OC was able to significantly reduce nasal obstruction, secretion and ocular symptoms, but not pruritus, rhinorrhea and sneezing. However, it has been demonstrated that the plasma cortisol level measured at three weeks was significantly reduced after daily administration of oral prednisolone. The beneficial effects of oral or depot steroids on AR symptoms and quality of life are significant when compared with placebo and oral antihistamines. Systemic and topical nasal corticosteroids have similar efficacy in controlling AR symptoms, although ocular symptoms respond better to systemic corticosteroids. Despite certain benefits of systemic corticosteroids (oral and depot) in the treatment of AR, international guidelines strongly recommend against their use due to concerns about adverse effects.^20^, ^502^

#### Intranasal corticosteroid

These are the most effective medications for treating all AR symptoms. Its onset of action is variable and, depending on the compound, can occur between three and 36 h after the first dose. Clinical control of symptoms can be rapid/however, to suppress chronic nasal inflammation it must be used for a minimum period of 60–90 days. The systemic bioavailability of Intranasal Corticosteroids (ICS) is very low, especially in relation to fluticasone, mometasone and ciclesonide. Table 10 presents the pharmacokinetic and pharmacodynamic characteristics of IC.^507^

**Table 10:** Pharmacokinetic and pharmacological determinants of adverse systemic events.

Drug	Bioavailability (%)	GCR^a^ affinity	Half life	Power	Lipophilia	Distribution volume	Inactivation upon 1st passage through the liver	Systemic power
Triamcinolone	46	233	Short	Low	Low	Low	Intermediate/High	Low
Beclomethasone	44	1345	Intermediate	Low/Intermediate	Intermediate/High	Intermediate	Intermediate	Intermediate
Budesonide	32	855	Short	Low	Low	Low	High	Low
Fluticasone propionate	<1	1775	Long	High	High	High	Extensive	High
Fluticasone furoate	<0.5	2989	Long	High	High	High	Extensive	High
Ciclesonide	<0.1	1212	Long	High	Intermediate/High	Intermediate/High	Extensive	High
Mometasone furoate	<0.1	2244	Intermediate/Long	High	High	Intermediate/High	Extensive	High

GCR, Glucocorticoid receptor.

a In relation to dexamethasone.

They rarely present adverse effects, the most common being those of a local nature such as irritation, epistaxis, sneezing, dryness and burning which, in general, depend on the dose used and the application technique. Therefore, patients should be instructed not to direct the spray towards the nasal septum, pointing the spray towards the nasal wings in order to avoid local irritation and bleeding.^507^

In Table 11 we present the main formulations available in Brazil. We highlight the withdrawal from the Brazilian market, in 2023, of topical nasal ciclesonide 50 mcg.

**Table 11:** Intranasal corticosteroids available in Brazil.^19^

Drug	Description	Dosage
Beclomethasone	Spray 50 mcg/dose	6‒12 years: 1–2 spray/nostril every 12 h
		>12 anos: 2 sprays/nostril each 12 h
Budesonide	Spray 32/50 mcg/dose	Children >6 years: 1–2 sprays/nostril 1×/day
	32/50/100 mcg/dose	
	32/50/64 mcg/dose	
Fluticasone propionate	Spray 50 mcg/dose	4–11 years: 1 spray/nostril, 1–2×/day
		>11 years: 2 sprays/nostril, 1– 2×/day
Fluticasone furoate	Spray 27.5 mcg/dose	2– 11 years: 1 spray/nostril 1×/day
		>12 years: 2 sprays/nostril 1×/day
Mometasone furoate	Spray 50 mcg/dose	2– 11 years:1 spray/nostril 1×/day
		>12 years: 2 sprays/nostril 1×/day
Triamcinolone	Spray 55 mcg/dose	4– 12 years: 1 spray/nostril 1×/day
	Spray 50 mcg/dose	>12 years 2 sprays/nostril 1×/day

#### Antihistamine and topical nasal corticosteroid combination

The combination of antihistamine and topical nasal corticosteroid in the same device aims to obtain synergistic and complementary effects of the medications, inhibiting the release of inflammatory mediators in the immediate and late phases of the allergic response and presenting anti-inflammatory action.^508^

In Brazil, the only combination of antihistamine and topical nasal corticosteroid marketed is Fluticasone Propionate and Azelastine hydrochloride (PF + AZE), at a dose of 50 mcg of fluticasone and 137 mcg of azelastine per dose. This combination is approved for use in AR from the age of six, in a fixed dose of one spray in each nostril twice a day.

Randomized clinical trials demonstrated that the PF + AZE combination is clinically superior to placebo and to azelastine and fluticasone alone for the total nasal symptom score, for the main symptoms of allergic rhinitis alone (nasal itching, runny nose, sneezing and nasal obstruction) and for ocular symptoms.^508^, ^509^, ^510^ Data from clinical trials indicate that more than 70% of patients treated with the PF + AZE combination show clinically relevant improvement after two weeks of treatment and, in real-life studies, around 80% show improvement clinically relevant in one week of treatment.^489^ Faster onset of action than other drugs used in the treatment of AR is highlighted as one of the advantages of the combination of antihistamine and topical nasal corticosteroid. In a study with nasal provocation in an exposure chamber, the combination PF + AZE showed improvement in nasal symptoms after 5 min, significantly higher than the 150 min observed for the combination of nasal fluticasone and oral loratadine.^510^

The PF + AZE combination also demonstrated action on other outcomes related to allergic rhinitis, such as improvement in smell, nasal hyperreactivity and quality of life.^508^ Adverse events resulting from the use of the PF + AZE combination have been infrequent and the report of serious adverse event, non-existing. The most common adverse events have been: dysgeusia, nausea, sneezing, nasal discomfort and epistaxis.^511^

A second combination of antihistamine and topical nasal corticosteroid, containing mometasone and olopatadine, was developed, but is not yet marketed in Brazil.^51^, ^52^, ^53^, ^54^, ^55^, ^56^, ^57^, ^58^, ^59^, ^60^, ^61^, ^62^, ^63^, ^64^, ^65^, ^66^, ^67^, ^68^, ^69^, ^70^, ^71^, ^72^, ^73^, ^74^, ^75^, ^76^, ^77^, ^78^, ^79^, ^80^, ^81^, ^82^, ^83^, ^84^, ^85^, ^86^, ^87^, ^88^, ^89^, ^90^, ^91^, ^92^, ^93^, ^94^, ^95^, ^96^, ^97^, ^98^, ^99^, ^100^, ^101^, ^102^, ^103^, ^104^, ^105^, ^106^, ^107^, ^108^, ^109^, ^110^, ^111^, ^112^, ^113^, ^114^, ^115^, ^116^, ^117^, ^118^, ^119^, ^120^, ^121^, ^122^, ^123^, ^124^, ^125^, ^126^, ^127^, ^128^, ^129^, ^130^, ^131^, ^132^, ^133^, ^134^, ^135^, ^136^, ^137^, ^138^, ^139^, ^140^, ^141^, ^142^, ^143^, ^144^, ^145^, ^146^, ^147^, ^148^, ^149^, ^150^, ^151^, ^152^, ^153^, ^154^, ^155^, ^156^, ^157^, ^158^, ^159^, ^160^, ^161^, ^162^, ^163^, ^164^, ^165^, ^166^, ^167^, ^168^, ^169^, ^170^, ^171^, ^172^, ^173^, ^174^, ^175^, ^176^, ^177^, ^178^, ^179^, ^180^, ^181^, ^182^, ^183^, ^184^, ^185^, ^186^, ^187^, ^188^, ^189^, ^190^, ^191^, ^192^, ^193^, ^194^, ^195^, ^196^, ^197^, ^198^, ^199^, ^200^, ^201^, ^202^, ^203^, ^204^, ^205^, ^206^, ^207^, ^208^, ^209^, ^210^, ^211^, ^212^, ^213^, ^214^, ^215^, ^216^, ^217^, ^218^, ^219^, ^220^, ^221^, ^222^, ^223^, ^224^, ^225^, ^226^, ^227^, ^228^, ^229^, ^230^, ^231^, ^232^, ^233^, ^234^, ^235^, ^236^, ^237^, ^238^, ^239^, ^240^, ^241^, ^242^, ^243^, ^244^, ^245^, ^246^, ^247^, ^248^, ^249^, ^250^, ^251^, ^252^, ^253^, ^254^, ^255^, ^256^, ^257^, ^258^, ^259^, ^260^, ^261^, ^262^, ^263^, ^264^, ^265^, ^266^, ^267^, ^268^, ^269^, ^270^, ^271^, ^272^, ^273^, ^274^, ^275^, ^276^, ^277^, ^278^, ^279^, ^280^, ^281^, ^282^, ^283^, ^284^, ^285^, ^286^, ^287^, ^288^, ^289^, ^290^, ^291^, ^292^, ^293^, ^294^, ^295^, ^296^, ^297^, ^298^, ^299^, ^300^, ^301^, ^302^, ^303^, ^304^, ^305^, ^306^, ^307^, ^308^, ^309^, ^310^, ^311^, ^312^, ^313^, ^314^, ^315^, ^316^, ^317^, ^318^, ^319^, ^320^, ^321^, ^322^, ^323^, ^324^, ^325^, ^326^, ^327^, ^328^, ^329^, ^330^, ^331^, ^332^, ^333^, ^334^, ^335^, ^336^, ^337^, ^338^, ^339^, ^340^, ^341^, ^342^, ^343^, ^344^, ^345^, ^346^, ^347^, ^348^, ^349^, ^350^, ^351^, ^352^, ^353^, ^354^, ^355^, ^356^, ^357^, ^358^, ^359^, ^360^, ^361^, ^362^, ^363^, ^364^, ^365^, ^366^, ^367^, ^368^, ^369^, ^370^, ^371^, ^372^, ^373^, ^374^, ^375^, ^376^, ^377^, ^378^, ^379^, ^380^, ^381^, ^382^, ^383^, ^384^, ^385^, ^386^, ^387^, ^388^, ^389^, ^390^, ^391^, ^392^, ^393^, ^394^, ^395^, ^396^, ^397^, ^398^, ^399^, ^400^, ^401^, ^402^, ^403^, ^404^, ^405^, ^406^, ^407^, ^408^, ^409^, ^410^, ^411^, ^412^, ^413^, ^414^, ^415^, ^416^, ^417^, ^418^, ^419^, ^420^, ^421^, ^422^, ^423^, ^424^, ^425^, ^426^, ^427^, ^428^, ^429^, ^430^, ^431^, ^432^, ^433^, ^434^, ^435^, ^436^, ^437^, ^438^, ^439^, ^440^, ^441^, ^442^, ^443^, ^444^, ^445^, ^446^, ^447^, ^448^, ^449^, ^450^, ^451^, ^452^, ^453^, ^454^, ^455^, ^456^, ^457^, ^458^, ^459^, ^460^, ^461^, ^462^, ^463^, ^464^, ^465^, ^466^, ^467^, ^468^, ^469^, ^470^, ^471^, ^472^, ^473^, ^474^, ^475^, ^476^, ^477^, ^478^, ^479^, ^480^, ^481^, ^482^, ^483^, ^484^, ^485^, ^486^, ^487^, ^488^, ^489^, ^490^, ^491^, ^492^, ^493^, ^494^, ^495^, ^496^, ^497^, ^498^, ^499^, ^500^, ^501^, ^502^, ^503^, ^504^, ^505^, ^506^, ^507^, ^508^, ^509^, ^510^, ^511^, ^512^, ^513^ Studies with this combination have demonstrated superior action to placebo in perennial allergic rhinitis^513^ and superior to isolated drugs in rhinitis seasonal allergy.^514^, ^515^

#### Disodiumchromoglycate

Disodium Cromoglycate (DSC) is a mast cell stabilizer, which prevents the release of inflammatory mediators, such as histamine, and does not have bronchodilator, antihistamine or intrinsic anti-inflammatory activity.^20^

By stabilizing mast cells, DSC blocks actions triggered by them, such as bronchospasm induced by allergens. It is derived from the Amni visnaga plant and traditionally used by ancient Egyptians for its antispasmodic properties.^502^

DSC blocks the function of chloride channels, which serve to regulate cell volume and prevents the influx of extracellular calcium into the cytoplasm of mast cells. Reduces the release of inflammatory mediators by inhibiting the degranulation of sensitized mast cells. It has been demonstrated that DSC has anti-inflammatory properties unrelated to the activation of mast cells, which prevents the action of inflammatory mediators, specifically macrophages, eosinophils, monocytes and platelet-activating factor.^516^

In treating patients with seasonal AR, 4% DSC administered four times daily for four weeks significantly improved nasal symptom scores compared to placebo when used in patients with house dust mite allergy, as well as the influx of neutrophils and reduction of platelet activating factor.^502^

DSC is less effective than topical nasal corticosteroids, is safe in children and approved for use in the first year of age; viable as a 4% nasal spray solution, but with a short half-life that requires administration three to six times per day. There are no reports of major adverse effects reported. Minor adverse effects include nasal irritation, burning, sneezing, epistaxis, and unpleasant taste.^502^

#### Leukotriene receptor antagonists

Leukotrienes (LTs) are lipid mediators formed from the enzymatic metabolism of arachidonic acid. LTs that contain cysteine amino acids (LTC4, LTD4, LTE4) are called LT-cysteines (cis-LT1) and constitute important mediators of the inflammatory response in asthma and AR. They cause vasodilation, plasma exudation, mucus secretion, in addition to eosinophilic inflammation, with consequent nasal obstruction.^517^

Montelukast Sodium (MS) is currently the only compound of this class available in Brazil, and has recognized superiority to placebo in controlling symptoms and improving the quality of life of patients with AR.^518^, ^519^ A systematic review, with the inclusion of six randomized clinical trials, showed that the association of ALTs with topical nasal corticosteroids, compared to the latter as monotherapy, was superior in controlling ocular symptoms, showing no significant impact on nasal symptoms and the patient's quality of life.^520^

Other studies point to divergent results, with research showing that ALTs have comparable efficacy to oral antihistamines, while others do not confirm this superiority, similar to the results of studies comparing ALTs with topical nasal corticosteroids in the treatment of AR.^268^, ^521^

ALTs should not be used as the first choice in monotherapy.^20^

ALTs could be an option for patients who have difficulty adapting and adhering to topical nasal treatment. Furthermore, they can be considered in cases of chronic rhinosinusitis with nasal polyposis, in Aspirin-Exacerbated Respiratory disease (ASR), where the exacerbated expression of LTC4 synthetase leads to excessive production of leukotrienes and exacerbation of rhinitis and/or rhinosinusitis. Despite the limited level of evidence, ALTs improve symptoms and CT scores in these patients.^522^

MS is available in the following presentations: 4 mg (granulated powder sachet or chewable tablet) for children between 6 months and 5 years; 5 mg (chewable tablet) for children between six and 14 years old, and 10 mg (tablet) for those aged 15 and over. It is well tolerated, and adverse reactions are mild and do not require discontinuation of treatment.^518^, ^519^

Recently neuropsychiatric adverse reactions have been associated with the use of MS, despite conflicting results. Sleep disturbances are especially described, including nightmares, insomnia, sleepwalking; anxiety, agitation, aggressive behavior or hostility; depression; psychomotor hyperactivity, restlessness and tremors.^523^

#### Leukotriene receptor antagonists associated with oral antihistamine

On the market for a few years, the association of ATLs with antihistamines has shown good acceptance by clinicians and patients and, to date, is approved for use in people over 18 years of age.^524^

Studies show that combined therapy is superior to both medications when administered alone.^268^, ^525^

A systematic review with meta-analysis, comparing different combinations of drugs for the treatment of AR compared to the use of antihistamines alone, showed that monotherapy with antihistamines is not competent and that the association with ALTs can significantly improve ocular symptoms.^526^

We currently have on the Brazilian market, a presentation that combines montelukast 10 mg + levocetirizine 5 mg.

### Allergen immunotherapy

#### Efficacy in the treatment of allergic rhinitis

Allergen Immunotherapy (AIT) is a precision medicine strategy used for more than a century^527^ to treat atopic diseases such as rhinitis and asthma. The guidelines of the American Academy of Allergy, Asthma and Immunology (AAAAI), European Academy of Allergy and Clinical Immunology, and the World Allergy Organization (WAO) are classic documents that define the scientific standards for the use of AIT.^528^, ^529^, ^530^, ^531^, ^532^ The Allergic Rhinitis and its Impact on Asthma (ARIA) initiative,^532^ took place during a World Health Organization workshop in 1999 and established guidelines for the treatment of AR based on allergic tests and a therapeutic approach using Evidence-Based Medicine ‒ EBM strategies (GRADE approach, Grading of Recommendations, Assessment Development and Evaluation). The ARIA recommendations recommend that AIT represents one of the pillars in the treatment of AR with a high GRADE level. In Brazil, recently, the parameters of good clinical practices and the Brazilian AIT guidelines in the treatment of AR were established, guiding and adapting the use of this therapeutic modality to the reality of our country.^533^, ^534^

Specialized clinical evaluation and identification of allergic sensitization, through the immediate reading prick test and/or investigation of allergen-specific serum IgE, represent the pillars of this precision medicine strategy enables the personalization of AIT treatment. House dust mites (Dermatophagoides pteronyssinus, Dermatophagoides farinae and Blomia tropicalis) are the most important allergens associated with the etiology of AR in Brazil. However, sensitization to pollens, in the southern region, and animal dander is also observed in the Brazilian population.^533^, ^534^

The effectiveness of the treatment is only achieved in carefully selected patients; therefore, adequate professional training to perform and interpret allergy tests and treat allergic and immunological diseases is essential. We must always consider that patients with AR often have other associated atopic diseases, such as asthma and AD, which must be investigated and treated appropriately. Furthermore, the association between AR, recurrent upper airway infections and primary immunodeficiencies may occur. Therefore, in particular cases, the assessment of the immunological response through specific tests is necessary to carry out a careful assessment based on knowledge of clinical immunology.^531^, ^532^, ^533^, ^534^

Several studies using the concepts of Evidence-Based Medicine (EBM) have demonstrated the effectiveness of AIT, both Subcutaneously (SCIT) and Sublingually (SLIT).^529^, ^530^, ^531^, ^532^, ^533^, ^534^, ^535^, ^536^, ^537^ The Brazilian Guidelines for AIT in the treatment of AR were recently published.^534^ This systematic review included 25 Randomized Clinical Trials (RCTs), double blind, placebo-controlled, with a total of 4,518 patients with perennial AR with or without asthma that underwent SCAIT with house dust mites (Dermatophagoides pteronyssinus and D. farinae, in a 1:1 ratio) and 3,887 control patients treated with placebo. The effectiveness of the treatment was proven, with a high GRADE level of evidence. For SCIT with grass or tree-derived pollen allergens, 22 RCTs were included. The joint analysis of these clinical studies using the GRADE approach revealed a level of efficacy considered moderate GRADE.

The effectiveness of SLAIT was also verified in 21 clinical trials analyzed in the Brazilian Guidelines for AIT^534^ in the treatment of AR containing a proportional mixture of the Dermatophagoides pteronyssinus and Dermatophagoides farinaemites. All RCTs showed clinical efficacy by reducing symptom and/or medication scores relative to the placebo group. The effectiveness of the treatment was proven, with a high GRADE level of evidence being observed. In this same systematic review, SLIT with grass and tree pollens also had proven efficacy (moderate GRADE).

In conclusion, all consensus in the field considers AIT to be the only treatment capable of modifying the allergen-specific immunological response, promoting robust and long-lasting desensitization and a state of tolerance in patients with AR.^528^, ^529^, ^530^, ^531^, ^532^, ^533^, ^534^, ^535^, ^536^ Unlike the use of pharmacological therapy and biologicals, this immunomodulatory strategy can promote remission and control of allergic diseases for prolonged periods, even after its administration has ended. Additionally, the use of AIT in patients with AR has preventive potential for the development of asthma. AR control symptoms remain satisfactory in the long term, even after the end of AIT, reducing or even abolishing the use of drugs. Remission of the disease lasts for at least seven to 10 years and can last throughout the individual's life. Therefore, we can consider this therapy potentially capable of promoting total remission of the disease.^534^, ^535^

#### Allergen immunotherapy methods

Currently in our country^533^, ^534^ there are two forms of treatment administration: Sublingual Immunotherapy Drops (SLIT) and Subcutaneous Immunotherapy (SCIT). SCIT is the main type of treatment carried out in the United States of America (USA). However, from 1990 onwards, when SLIT was successfully introduced in Europe, American doctors became interested in this new form of application. Despite this interest, SLIT, as it was not approved by the North American FDA, was little used in that country for many years, exclusively off label. Since April 2014, when the FDA approved SLIT in pill form, American doctors have become more comfortable prescribing SLIT, which continues to be used off-label in drops.^535^, ^536^

The European Academy of Allergy and Clinical Immunology (EAACI) guidelines recommend both forms of application (SCIT and SLIT) for the treatment of AR or allergic rhinoconjunctivitis, perennial or seasonal, in children and adults. Allergic disease must necessarily be mediated by IgE antibodies to clinically relevant allergens, to one or more allergen groups, especially in patients with moderate or severe allergies, whose symptoms affect quality of life or nighttime sleep. The recommendations for good clinical practices in AIT from ASBAI (Brazilian Association of Allergy and Immunology) are in accordance with these EAACI guidelines.^533^, ^534^, ^535^, ^537^

For indication and better treatment efficacy, the etiological diagnosis of AR, responsible for sensitization mediated by IgE antibodies, is crucial, determining its clinical relevance. The appropriate choice and adequate management of allergenic extracts to be used in the personalized vaccine used in AIT is a fundamental condition for achieving the expected results in clinical practice. In Brazil, CFM RESOLUTION nº 2,215/2018, regulates the use of allergenic extracts for diagnostic and therapeutic purposes in allergic diseases.^538^ The specialist physician, holder of Specialist Certificate -EQR in allergy and immunology and/or area of expertise in pediatric allergy, must carry out the formulation of the components of the allergenic extracts and their use in different dilutions appropriately for the appropriate choice of the route of administration (SCIT or SLIT) and its application scheme, which must be personalized for each patient, according to the results of the allergic tests and clinical evaluation.^533^, ^534^ Also, it is fundamentally important to know the properties of allergens so that the specialist can choose whether or not to mix certain allergens in cases of polysensitized patients.^533^, ^534^, ^535^, ^536^, ^537^

Regarding age, AIT is indicated from two years of age for sublingual treatment and over five years of age for subcutaneous treatment, up to approximately 65 years of age, for both routes of application. The doctor must carefully analyze, using his technical knowledge, each case individually and, together with the patient or his guardian, choose and decide the best route of administration of immunotherapy with allergens.^528^, ^529^, ^530^, ^531^, ^532^, ^533^, ^534^

#### Immunological effects

Currently, several mechanisms of action are known to act together to promote immunomodulation and generate a state of specific allergen immunological tolerance.^36^, ^539^, ^540^, ^541^, ^542^, ^543^, ^544^, ^545^, ^546^, ^547^ This specificity of the AIT effects and the personalized indication based on the results of allergic tests interpreted by a specialist physician in allergy and immunology configure this therapy as a precision medicine strategy.^548^, ^549^

The initial response to both types of AIT application routes occurs through the induction of phenotypic changes in circulating dendritic cells, which contributes to the suppression of the inflammatory allergic response via the action of the population of allergen-specific regulatory T-cells (Treg), inhibiting type 2 inflammation. AIT reduces both the activity of type 2 Innate Lymphoid Cells (ILC2), in innate immunity, and of Th2 cells, in acquired immunity. The production of Interleukin 10 (IL-10) and Transformed cell Growth Factor (TGF-beta) by Treg cells are key events in the inhibition of type 2 inflammation. This immunomodulation promoted by Treg cells is observed between three and six months after the start of SCIT or SLIT.^533^, ^534^, ^543^, ^544^, ^545^, ^546^, ^547^

Accelerated SCIT regimens (cluster or rush) can induce immunological tolerance earlier. The shift towards allergen-specific responses with a Th1 profile, with increased production of gamma Interferon (IFN-γ) occurs approximately 12 months after the onset of AIT. In the antibody-mediated immune response, the effects observed are increases in allergen-specific IgA and IgG4. Regarding these mechanisms of induction of allergen-specific desensitization, no important differences were detected regarding the immunomodulation pathways observed in SCIT and SLIT.^533^, ^543^, ^545^, ^546^, ^547^, ^550^

The duration of treatment is three to five years, defined as the period necessary for the effects to be achieved and maintained in the long term, even after the end of the AIT.^528^, ^531^, ^533^, ^534^ The recommended treatment duration is counted from the maintenance phase, corresponding to the moment at which the effective dose capable of controlling symptoms was reached. Currently, studies with immunological biomarkers such as IgG4 and specific IgE still present conflicting results and are not used in clinical practice to monitor efficacy or even to suspend treatment, being restricted to the field of research.

#### AIT safety

Experimental studies, clinical trials and real-life experience reports have revealed a good safety profile of SCIT. Around 1% of injections may present adverse reactions, whether in children or adults. The most common are at the injection site, such as discomfort, erythema, edema, pain and itching; these are generally mild reactions.^529^, ^530^, ^531^, ^533^, ^534^, ^535^

However, systemic reactions may also occur, most often mild, including sneezing, itching, nasal congestion and/or hives, which are easy to control and do not impede the continuation of SCIT. Patients who present frequent and extensive local reactions should be evaluated with caution, as they may eventually be at greater risk of systemic reactions.

In patients with AR and concomitant asthma, it is always recommended to evaluate an acute exacerbation of asthma symptoms as well as measure the peak flow before applying SCIT and this should be suspended in the presence of an acute asthma exacerbation. The safety assessment carried out in the systematic review: Brazilian Guidelines for ITA in the treatment of AR^534^ revealed that SCIT with household dust mites is safe in the treatment of AR in children and adults (high GRADE). Additionally, SCIT with pollens also presented evidence of safety in its use in children and adults (moderate GRADE).

There can be serious systemic reactions, although rare. Anaphylaxis and even death have been reported in the literature. Therefore, SCIT applications require a location with appropriate infrastructure, in accordance with the Annex to Resolution CFM2,215/2018 (Federal Council of Medicine)^538^ and immediate medical care. In cases of anaphylaxis, the treatment of choice is the intramuscular application of epinephrine/millesimal adrenaline (1/1000). Antihistamines and systemic corticosteroids are considered secondary medications. The application of SCIT must always be carried out under medical supervision and the patient must remain under observation for a minimum period of 30 min. It is recommended that the SCIT be performed at the prescribing physician's unit.^528^, ^531^, ^533^, ^545^^,^^546^

Meta-analyses and systematic reviews have postulated that SLIT is safe and effective in children and adults. SLIT is well tolerated, even at high doses, with good clinical safety. Serious systemic reactions are exceedingly rare with few reports in the literature.^528^, ^529^, ^530^, ^531^, ^532^, ^533^, ^534^, ^535^, ^545^, ^546^ The safety assessment carried out in the review: The systematic Brazilian Guidelines for AIT in the treatment of AR^534^ revealed that SLIT with house dust mites is safe in the treatment of AR in children and adults (GRADE high). Additionally, SLIT with pollens is also safe. SLIT with pollens is safe in the treatment of AR in children and adults (moderate GRADE).

In most patients undergoing SLIT, the predominant adverse effects are mild or moderate oral reactions, such as itching and irritation in the mouth and throat. Many of these effects are observed at the beginning of treatment (in the induction phase). Also, tingling sensations (oral paresthesia), lip edema, tongue edema, glossodynia, dysgeusia, abdominal pain, diarrhea and headache have been reported. Cough and dyspnea are likely to occur in patients who present allergic rhinitis concomitantly with asthma.^534^, ^536^, ^538^, ^551^ Due to the excellent safety profile, SLIT can be administered at home. It is recommended that the first dose be administered under medical supervision, especially at the beginning of a new concentration. Most adverse reactions are mild (itching of the oral mucosa, lip edema, runny nose and nausea). However, even with a high safety profile, SLIT can cause systemic reactions, particularly in patients with asthma.^528^, ^531^, ^533^ In conclusion, the safety of SLIT depends on specific indications, careful evaluation of the clinical history and a specialized professional clinical approach.

#### Contraindications

The contraindications of AIT are:

• All patients with a history of asthma should be carefully evaluated and lung function measured by spirometry with bronchodilator testing. Poorly controlled asthma and patients with Forced Expiratory Volume in one second (FEV1) below 70% are absolute indications.
• Serious active diseases (especially immunological, infectious and neoplastic diseases) are absolute contraindications to the use of AIT. Controlled cardiovascular diseases, use of ACE inhibitors, beta blockers, controlled chronic diseases, mild psychiatric illnesses, are relative contraindications where the risk versus benefit must be assessed individually.
• Pregnancy and lactation are conditions that absolutely contraindicate the beginning of treatment, but not its continuation, when increasing AIT concentration is contraindicated if it is in the induction phase.
• Individuals with eosinophilic esophagitis have an absolute contraindication for the use of SLIT.

### Immunobiological agents

#### Anti-IgE

Patients with asthma have a high prevalence of type 2 immunological diseases, of which AR is the most prevalent comorbidity, but chronic rhinosinusitis and atopic dermatitis are also common.^551^

These inflammatory conditions that occur with high T2 are characterized by infiltration of eosinophils, basophils, mast cells and other inflammatory cells, and are recognized by the robust expression of cytokines associated with Th2 effector cells, specifically the Interleukins (IL) IL-4, IL-5 and IL-13, and alarmins TSLP (Thymic Stromal Lymphopoietin), IL-33 and IL-25. The term T2 high is considered the preferred term for this inflammatory phenotype, in recognition of the numerous additional sources of Th2 effector cytokines, such as Innate Lymphoid 2 Cells (ILC2s), eosinophils, mast cells, and many others.^552^, ^553^, ^554^

At least 80% of patients with CRSwNP (chronic rhinosinusitis with nasal polyps) in Western countries have type 2 inflammation in nasal polyps, and biologics are able to reduce polyps by blocking elements of this type of immune response.^552^

It has been shown by cluster analyses, biological assays and animal studies that eosinophils are elevated in at least two subtypes of asthma: one represented by allergic asthma with a strong adaptive immune response and the other with an intense response driven by ILC2. These analyzes have not been conclusively conducted on AR.^553^

Although only approved for the treatment of moderate to severe persistent asthma and chronic idiopathic urticaria, omalizumab has been extensively studied in the treatment of RA, both as direct therapy and as complementary therapy.^555^, ^556^, ^557^, ^558^, ^559^

With the aim of examining whether the response to omalizumab in terms of asthma control is associated with a greater probability of a good response to rhinitis in patients with both conditions, it was demonstrated that the probability of improvement in rhinitis was significantly greater among patients who used omalizumab. This study demonstrates that good asthma control after omalizumab therapy was associated with a greater likelihood of rhinitis improvement.^560^

Meta-analysis published in 2014 selected 352 citations, with 78 papers eligible for review. Of these studies, 11 were qualified for evaluation, with 2,870 patients randomized. In the nine studies that measured daily symptom scores, omalizumab significantly reduced these scores. The use of rescue medication also decreased in studies that evaluated this outcome. There were no significant differences in adverse effects when comparing omalizumab with placebo.^561^

Meta-analysis identified 83 papers on omalizumab for the treatment of AR and, of these, 16 were randomized clinical trials. The latter were the focus of the analysis and involved a total of 3,458 patients (1,931 omalizumab and 1,527 controls). The results showed statistically significant differences in favor of the group that used omalizumab, both in the daily score of nasal symptoms and ocular symptoms in the proportion of days using medication for attacks, quality of life questionnaires specific to rhinoconjunctivitis and global assessment by investigators. It is important to highlight that there was no statistically significant difference in terms of adverse events between the groups.^562^

#### Other biological agents

Other than omalizumab, none of the other biologics targeting T2 inflammation have been studied specifically in AR. However, several studies have evaluated the effectiveness of these medications in treating AR in the context of comorbid asthma. No convincing studies on biologics targeting IL-5/IL-5R have been performed in AR. In one study, although no specific analysis of nasal symptoms was reported, mepolizumab (humanized anti-IL-5 antibody) significantly reduced not only asthma exacerbations but also improved assessment of quality of life in patients with severe asthma and upper airway disease.^554^

Dupilumab is a fully human mAb that targets the IL-4 Receptor alpha subunit (IL-4Rα) and therefore inhibits the signaling of IL-4 and IL-13 that share this same receptor. In a phase 2b study subanalysis, the efficacy and safety of this biological product were examined in a subgroup of asthmatic patients with perennial AR, totaling 241 (61%) patients. In these, dupilumab 300 mg subcutaneously every two weeks versus placebo significantly improved the total SNOT-22 score and the four symptoms associated with AR assessed (nasal obstruction, runny nose, sneezing, and post-nasal secretion). Although promising, these results need to be confirmed in additional studies to evaluate the efficacy, safety and cost-effectiveness of dupilumab for the treatment of AR.^563^

A clinical trial involving adults with seasonal AR caused by grass pollen evaluated the hypothesis that the addition of dupilumab to SCIT would increase its efficacy and improve its tolerability in these patients. Participants underwent nasal allergen challenge before and after treatment with dupilumab 300 mg every two weeks for 16 weeks. Dupilumab improved the tolerability of SCIT but did not reduce post-allergen challenge nasal symptoms compared with SCIT alone.^564^

Post-hoc analysis evaluated the efficacy of Tezepelumab in PATHWAY trial participants with perennial allergy. Tezepelumab treatment reduced asthma exacerbations, improved lung function, and reduced type 2 biomarkers in patients with severe, uncontrolled asthma, with or without perennial allergy. However, around 50% of participants had rhinitis, the results of treatment in this specific group were not analyzed.^565^

TSLP inhibition increases the efficacy of SCIT in patients with cat allergy during therapy and may promote tolerance after one year of treatment verified by nasal challenge and skin testing. Transcriptomic analysis of nasal epithelial samples demonstrated that treatment with the SCIT/Tezepelumab combination caused persistent downregulation of a genetic network related to type 2 inflammation that was associated with improvement in nasal challenge responses.^1^

Blocking the main transcription factors involved in type 2 inflammation, GATA3 and Signal Transducer and Activator of Transcription (STAT) 6, is not a straightforward process to achieve with low molecular weight drugs.^553^

However, the development of a GATA3-specific DNase that inhibits type 2 inflammation and experimental allergic asthma in humans increases future prospects for treatment for allergic diseases.^567^

Janus Kinase (JAK)-STAT inhibitors are effective in atopic dermatitis and other immunological diseases. According to some authors, inhibition of this signaling pathway could be useful in chronic rhinosinusitis.^568^

Therapy with selective immunomodulators, such as biologic agents, allows for systemic treatment that could potentially cover multiple concomitantly occurring atopic/allergic conditions. Furthermore, personalized treatments have the potential to reduce side effects while improving therapeutic results and, in general, have an acceptable safety profile.^569^, ^570^ Because current AR treatments are satisfactory, the cost of biologics makes their use in the management of AR unfeasible. AR as a stand-alone medication unless indicated for comorbidities such as AD and asthma.

Table 12 presents the main perspectives and needs for the use of biologicals in AR.

**Table 12:** Main needs for the use of biologicals in allergic rhinitis.

IgE-mediated allergy diagnosis
Severe cases, uncontrolled with the recommended treatment
Better patient phenotype and endotype characterization
T2-inflammatory biomarkers – peripheral blood and nasal eosinophils, IgE serum levels
Having comorbidities
Accessibility and cost
Treatments approved by the National Health Surveillance Agency

### Alternative and non-traditional therapies

#### Acupuncture

Acupuncture is a traditional method of Chinese medicine, its basic principle being the insertion of fine needles into specific points that regulate the flow of vital energy (known as Qi) and which flow along meridians just underneath the skin. Once stimulated, they can cause the restoration of balance due to a disease or energy imbalance.^571^, ^572^ In Chinese medicine, it has been used in many diseases and in otorhinolaryngology since the 5th century BC, being recommended as a complementary treatment in international rhinitis guidelines since 2015.^20^, ^573^, ^574^

Several studies have shown that acupuncture can modulate some biomarkers such as: neuropeptides (substance P and vasoactive intestinal peptide VIP), in addition to other inflammatory mediators, regulating the Th1/Th2 imbalance and consequently the levels of total IgE and IL-10.^575^, ^576^, ^577^

Despite being a risk-free therapy with few side effects, the practice of acupuncture in AR appears to be very heterogeneous and with results that are often inconsistent. In this way, it has been tested in diverse ways, in particular using application points outside those recommended by traditional Chinese medicines, placebo, the so-called “Sham points”. Some systematic review studies, with or without meta-analysis, have shown improvements in symptom and quality of life scores and a reduction in the use of rescue medications,^576^, ^578^ when compared with conventional treatment.

He and colleagues conducted a recent Cochrane systematic review of 30 randomized studies with 4413 participants, mostly adults. The duration of the study varied between two and 12 weeks, comparing the use of acupuncture vs not using any medication, or vs the use of Sham points (acupuncture placebo) or vs antihistamines or associated with antihistamines vs just antihistamines.^579^ The results showed that acupuncture was superior to the non-use of medication and acupuncture placebo (Sham points) both in the intensity of nasal symptoms and quality of life and that the effects of acupuncture were comparable to the use of antihistamines. It was not possible to establish the effects of acupuncture on AR in children and adolescents due to the small number of studies in this age group.

The most recent data on the use of acupuncture in AR have sought to understand the best method for selecting Qi stimulation points to be used, the type of needles or even the use of other forms of stimulation (moxibustion, pressure on the ear, fine needles in the sphenopalatine ganglion, among others).^580^, ^581^

Studies show that care must be taken in terms of side effects when using electro acupuncture in patients with pacemakers and other devices, and in pregnant women where it can induce labor.

In conclusion, acupuncture has a role in the treatment of AR as an adjuvant treatment, especially in patients who refuse conventional treatment.^20^

#### Other complementary modalities

Some studies have shown that complementary and alternative therapies, such as moxibustion (Thunder Fire Moxibustion) can improve the clinical symptoms of patients with AR and reduce the incidence of adverse reactions. Moxibustion, a resource of Traditional Chinese Medicine (TCM), is a type of thermal acupuncture, made by burning the Artemisia sinensis and Artemisia vulgaris herbs, which involves burning these leaves for caloric stimulation of acupuncture points and meridians, to adjust functions, physiological and biochemical disorders of the human body, so as to achieve the objective of treating diseases.^582^

A systematic review carried out in 2022 demonstrated that moxibustion is a safe and effective treatment for AR. Due to the low quality of eligible trials and the low level of evidence, caution should be taken with conclusions. More high-quality, multicenter, randomized controlled clinical trials with larger sample sizes will be needed in the future to verify its clinical efficacy in the treatment of AR.^582^

#### Herbal therapy

Traditional Chinese herbal medicine has been practiced for over 80 centuries and continues to evolve. The Chinese pharmacopoeia has more than 13 thousand medicines and more than 100 thousand herbal combinations recorded in ancient literature. Although the prevalence and use of traditional Chinese herbal formulations increases globally, there are limited high-quality, large-scale multicenter trials validating their safety and effectiveness.^573^

Traditional Chinese medicine studies have revealed positive benefits in treating AR; however, many have small sample sizes, investigate different medications, and have potential methodological problems.^583^

Chinese herbal medicine has enormous potential in relieving symptoms, modulating the levels of immunological factors and reducing relapses in rhinitis. However, its efficacy and safety in treating rhinitis still need to be confirmed. More high-quality research is needed to provide reliable evidence for the clinical application of this therapy.^584^, ^585^

### Clinical control assessment

#### Visual Analogue Scale (VAS)

The VAS is a simple and widely used tool to assess the severity of symptoms and the impact of AR. The VAS had a significant correlation with other rhinitis severity indicators such as the Quality-of-Life Questionnaire Score (QLQS) and the rhinitis severity classification by the ARIA initiative. The VAS is usually graduated from 0 to 100 mm or 10 cm, with 0 being the absence of symptoms/discomfort. The VAS can be used for different questions, the most common being control of rhinitis symptoms in the last day or last week. In a validation study, VAS demonstrated to be sensitive in detecting changes in symptoms and quality of life and the smallest clinically important difference was 23 mm (scale from 0 to 100).^586^ Several medical societies recommend the use of VAS in monitoring symptoms of AR using the following classification: mild/controlled symptoms 0–20 mm; moderate/partially controlled symptoms 21–49 mm; intense/uncontrolled symptoms 50–100 mm.^587^, ^588^

### Questionnaires

Several questionnaires or assessment scores for rhinitis control have been proposed in recent years. In general, these questionnaires differ in the focus given to the concept of control, sometimes addressing more intensely the symptoms of the disease, sometimes valuing the impact of AR consequences on activities and daily life. Furthermore, there are specific questionnaires for the assessment of rhinitis and questionnaires that simultaneously addressing allergic rhinitis and asthma.

#### Rhinitis Control Assessment Test (RCAT)

The RCAT is a simple, self-administered questionnaire with six questions that assesses, in the last week, the intensity of nasal and ocular symptoms, the interference of rhinitis on sleep and activities and the patient's personal assessment of the control of their disease.^589^ This questionnaire was validated in adults, proving to be reliable and reproducible.^589^ Each question is scored from 1 to 5 and the questionnaire score varies from 6 to 30 points, with scores equal to or lower than 21 indicating non-control of rhinitis.^589^ The RCAT was translated into Portuguese (Brazilian culture) and validated in adolescents with good discriminatory power to separate patients with controlled and uncontrolled AR.^590^

#### Asthma and Allergic Rhinitis Control Test – Control of Allergic Rhinitis and Asthma Test (CARAT)

CARAT is a questionnaire that jointly assesses asthma and AR control. This questionnaire, originally developed in Portuguese (Portuguese culture), has 10 questions that address upper and lower airway symptoms, sleep changes, activity limitations and medication use. CARAT evaluates the period of the last four weeks, varies from 0 to 30 points and scores above 24 indicate good control of rhinitis and asthma.^591^ The minimum clinically significant difference is 3.5 points. The questionnaire has good psychometric properties, such as internal consistency, reliability and responsiveness.^592^

A pediatric version of this questionnaire, called CARATKids, was developed for use in children aged six to 12 years. This version has 13 questions with affirmative or negative answers, eight for children and five for parents. The CARATKids total score ranges from 0 to 13 points, a score equal to or greater than points indicating no control of asthma and rhinitis and the minimum clinically crucial difference is 3 points.^593^, ^594^ CARATKids was adapted and validated for Brazilian Portuguese.^595^

#### MASK-air

MASK-air is an application for mobile devices that contains daily questionnaires to record the impact of AR symptoms. This app was developed in 2015 and is available in many countries like Brazil. MASK-air uses the visual-analog scale (VAS, scale 0–100 mm) to measure several issues, such as the impact of nasal and ocular symptoms and sleep quality. In a validation study, MASK-air proved to be an accurate instrument for daily assessment of the AR impact.^596^ In recent years, this instrument has been used in several real-life studies on allergic rhinitis and in comparing the therapeutic action of medications.^596^, ^597^

#### Rhinitis control scoring system

The Rhinitis Control Scoring System (RCSS) is a questionnaire developed to quantitatively assess the control of allergic rhinitis over a week. In this questionnaire, five symptoms (sneezing, anterior rhinorrhea, nasal obstruction, nasal itching and signs of conjunctivitis) are evaluated individually both in intensity (in percentage) and frequency, each item has a score of 2%–10%. The sum of these scores provides a final score that represents the patient's percentage of control, ranging from 20% (worst control) to 100% (best control). This score was validated in adults, showing a strong correlation with a specific quality of life questionnaire and nasal symptoms score and a moderate correlation with the visual analogue scale.^597^

#### Allergic rhinitis control test

The Allergic Rhinitis Control Test (ARCT)^7^ is a self-administered questionnaire that assesses allergic rhinitis control based on symptoms over the last two weeks. It consists of five questions scored individually from 1 to 5, which are added together to obtain a score that varies from 5 (worst score) to 25 (best score).^598^

The questions consider impaired quality of life, irritability, impact on sleep, use of additional medications to control rhinitis and the patient's perception of disease control. Rhinitis is considered controlled when the sum reaches a score ≥20. This cutoff point has a sensitivity of 55%, specificity of 90% and a positive predictive value of 94%.^599^

The ARCT has already been used in prospective studies in adults, demonstrating that it is a good objective tool for directing pharmacotherapy in AR.^600^

## Other rhinitis

### Infectious rhinitis ‒ viral/bacterial/fungal

#### Viral and bacterial

For many authors and, in the daily practice of clinicians and specialists, viral rhinitis is synonymous with the common cold, and, in fact, they are ways of reporting the same condition.^1^ The term viral rhinitis should be used to describe the involvement of the nasal mucosa by virus, in the clinical illness of the common cold.^601^

The same difficulty in understanding occurs in bacterial rhinitis, which, given the lack of direct evidence of bacterial infection of the nasal mucosa, is defined as a bacterial complication of viral rhinitis, affecting the nasal cavity and adjacent compartments, such as the paranasal sinuses.^601^

These are common conditions, especially in children with seven to 10 episodes per year.^602^

It is worth remembering that the antiviral and antibacterial host defense mechanisms available to the nasal mucosa are competent. For this reason, nasal infection by extracellular bacterial pathogens is rarely established and infection by respiratory viruses is self-limited, with short-term morbidity and no mortality.^2^ However, for at-risk populations, these infections predispose to more serious complications involving the sinuses, the face, middle ear and lungs.^602^

In terms of etiology, viral rhinitis/common colds are caused by a diversity of viruses, including multiple strains of rhinovirus, coronavirus, influenza virus, parainfluenza virus, respiratory syncytial virus, adenovirus, enterovirus, among others. In total, it is estimated that more than 200 viral strains are involved.^603^, ^604^ Incubation typically lasts 2–4 days.^605^

Nasal symptoms may be related to the direct action of the virus on the mucosa, but also to an immune-mediated response,^606^, ^607^ and include typical nasal congestion, sneezing, runny nose, cough - sometimes productive, sore throat of varying intensity and malaise, which causes a negative effect on general well-being.^607^ Often, and depending on the etiological agent, symptoms are not restricted to the respiratory tract (Table 13).^602^, ^606^, ^607^

**Table 13:** Viral infectious rhinitis.

	Common viruses	Influenza	SARS-CoV- 2	Allergic
Symptoms duration	Gradual onset duration <14 days	Sudden onset duration 7–14 days	Variable evolution duration 7–25 days	Sudden onset duration: many weeks
Sneezing	Common	No	No	Common
Nasal secretion and obstruction	Common	Sometimes	Rare	Common
Sore throat	Common	Sometimes	Sometimes	Sometimes (mild)
Cough	Common (humid)	Common (dry)	Common(dry)	Rare (dry)
General pain	Common	Common	Sometimes	No
Loss of smell and taste	Sometimes	Sometimes	Common	Rare
Fever	Rare	Common	Common	No

It has been demonstrated that upper airway infections caused by rhinoviruses play an important role in the etiology of acute rhinosinusitis, due to decreased mucociliary transport, mucosal edema and obstruction of paranasal sinus ostiae.^602^, ^608^ A possible complication of acute viral rhinitis should be suspected for post-viral rhinosinusitis if, in the course of the common cold, symptoms worsen after the fifth day of illness or symptoms persist for more than 10 days.^602^, ^608^

Infection with the influenza virus (type A) is related to complications in children under two years of age, elderly patients, patients with heart disease, nephropathy, liver disease, hematological diseases, lung disorders, metabolic disorders or immunodeficiency.^609^

Seeking to reduce the morbidity and mortality of these complications, effective prevention and treatment measures have been sought. As the viruses that cause rhinitis are transmitted through interpersonal contact, the most appropriate prophylactic measures are good hygiene and avoiding symptomatic contacts. The prophylactic efficacy of vaccination and passive immunoglobulin therapy has been demonstrated for influenza, SARS-CoV2, and RSV infections, respectively.^601^, ^602^

Existing pharmacological treatments for viral rhinitis are palliative, while antiviral treatment has theoretical efficacy and has not been shown to reduce the risk of complications.^602^

Studies have not shown significant evidence of benefit in using antibiotics for the common cold (viral rhinitis) or even for acute purulent rhinitis (bacterial rhinitis) even with persistent symptoms in children or adults (four studies with 723 participants – RR = 0.73, 95% CI 0.47–1.13).^610^ Given the evidence that these drugs cause significant adverse effects in adults and children, their use is not routinely recommended.

#### Fungal

As fungi are present throughout the environment, human exposure is inevitable and normal breathing will routinely deposit fungal elements in the nose and paranasal sinuses, which, usually, have no relevant consequences.^611^

In certain situations, fungal species can cause sinonasal disease, with clinical results ranging from mild to severe symptoms, such as invasive intracranial infection and even death.^611^, ^612^

The involvement of the nose and paranasal sinuses by fungi is categorized based on the degree of fungal invasion in local tissues.^611^, ^612^

It is worth mentioning that epidemiological studies, comparing patients with rhinosinusitis and normal controls, showed high rates of fungi identification in both groups, stressing the idea that the simple identification of fungus does not determine the disease, but it is rather a result of the host's immunological status.^611^, ^612^, ^613^

#### Allergic fungal rhinosinusitis

Individuals with suspected fungal AR present symptoms typical of other chronic rhinosinusitis, including nasal congestion, facial pain/pressure, nasal discharge and decreased sense of smell. From an epidemiological standpoint, patients with allergic fungal rhinosinusitis are younger, more likely to be male, and more likely to be of African descent.^614^, ^615^ Most patients have an intact immune system and often a history of atopy, including allergic rhinitis and/or asthma. The diagnosis of allergic fungal rhinosinusitis is often suspected based on radiographic characteristics.^614^, ^615^

More recently, the Working Group of the International Society of Human and Animal Mycology clarified the pathophysiology of fungal allergic rhinosinusitis, where the condition would be a subtype of chronic rhinosinusitis with nasal polyps.^616^ Histologically, the patient with chronic rhinosinusitis with nasal polyps presents eosinophilic mucin, in cases where fungal hyphae are detectable within the mucin, the diagnosis of fungal rhinosinusitis is considered, fungal allergic rhinosinusitis, if there is proven type 1 hypersensitivity to fungi, if there is no involvement of IgE mediation, it is characterized by a eosinophilic fungal rhinosinusitis (Table 14).^611^, ^612^, ^617^

**Table 14:** Clinical and laboratory characteristics of allergic and eosinophilic fungal rhinosinusitis.^611^, ^612^, ^616^

Fungal allergic rhinosinusitis	Eosinophilic allergic rhinosinusitis
Polyps	Polyps
Eosinophilic mucin	Eosinophilic mucin
Fungal hyphae (+)	Fungal hyphae (+)
(+) allergic test	(−) allergic test
IgE-mediated inflammation	Non-IgE mediated inflammation
Immunotherapy may be effective	Non-effective Immunotherapy

From the point of view of the clinical approach, this distinction is only important to the extent that immunotherapy (subcutaneous or sublingual) could be considered in allergic fungal rhinosinusitis, whereas it would not be indicated in eosinophilic fungal rhinosinusitis.^616^ Apart from immunotherapy, the clinical treatment of the two conditions is not distinguished.

#### Non-allergic eosinophilic rhinitis

Non-Allergic Eosinophilic Rhinitis (NAER) is one of the most common types of inflammatory non-allergic rhinitis, first described in 1981.^20^ The presence of eosinophils in the nasal respiratory mucosa and mucus identifies the cause of inflammation in this location. It is characterized by symptoms consistent with perennial allergic rhinitis, without atopy, but with the presence of local eosinophilia observed on nasal cytology. The pathophysiology of NAER is not well understood, but a key component involves ongoing chronic local eosinophilic inflammation with non-specific release of histamine.^20^

Patients with NAER report symptoms similar to those of perennial AR: nasal congestion, abundant rhinorrhea, sneezing, and nasal and ocular itching. A prominent feature of NAER is olfactory dysfunction. Patients with NAER demonstrate significantly higher thresholds on smell tests than patients with seasonal and perennial AR.^20^

The diagnosis is made based on a careful history. The findings on physical examination are not quite different from those found in patients with perennial AR (pale and hypertrophied turbinates), and negative skin or in vitro tests for allergy. Nasal cytology is characterized by prominent eosinophilia, 3%–25% in nasal smears, depending on the studies. Furthermore, nasal biopsies from these patients commonly show an increase in the number of mast cells with prominent degranulation.^20^

Studies have proven the role of chronic inflammation in the development of NAER with an increase in trans-endothelial migration of eosinophils in nasal lavage, which are attracted and activated by chemokines and cytokines. Specifically, NAER is characterized by elevated levels of tryptase and eosinophilic cationic protein in nasal fluid. Elevated levels of Interleukin (IL)-1β, IL-17, Interferon (IFN)-γ, Tumor Necrosis Factor (TNF)-α, Monocyte Chemoattractant Protein (MCP)-1, and RANTES (expressed and presumably secreted after normal activation of T cells) in nasal fluid were found in NAER compared to controls.^20^, ^618^, ^619^, ^620^, ^621^, ^622^, ^623^

NAER may occur in isolation but may be associated with or be a precursor to Aspirin-Associated Respiratory disease (ASR), a risk factor for the induction or exacerbation of obstructive sleep apnea, and a greater tendency for lower airway hyperreactivity.^20^

NAER is primarily treated with intranasal corticosteroids, which decrease neutrophil and eosinophil chemotaxis, reduce mediator release from mast cells and basophils, and result in reduced mucosal edema and local inflammation.^624^ A pooled analysis of three prospective, double-blind studies randomized, placebo-controlled trials of 983 patients, 309 of whom were classified as NAER, demonstrated a positive treatment effect using ICS with improvement in symptoms of nasal obstruction, post-nasal drip and rhinorrhea.^625^ Additionally, the intranasal antihistamine azelastine and Leukotriene Receptor Antagonists (LTRA) have been shown to reduce symptoms of rhinitis, including postnasal drip, sneezing, rhinorrhea, and congestion.^626^

### Drug-induced rhinitis

Drug-induced rhinitis is one in which symptoms are secondary to systemic medications. Classically it can be classified into three types:^20^

- Local inflammatory: Occurs when the use of a medication causes a direct change in inflammatory mediators in the nasal mucosa;
- Neurogenic: Occurs after the use of a medication that systemically modulates neural stimulation, leading to subsequent changes in the nasal mucosa; and
- Idiopathic: Applied when a well-defined mechanism has not been elucidated.

Local inflammatory type: Systemic ingestion of Non-Seroidal Anti-Inflammatory Drugs (NSAIDs) in specific patients can cause respiratory symptoms and be associated with nasal polyposis and asthma due to abnormal metabolism of arachidonic acid. NSAIDs inhibit Cyclooxygenase (COX)-1, leading to decreased production of prostaglandin E2 and increased production of Leukotrienes (LT) due to an imbalance towards the lipoxygenase pathway. Reduced PGE2 and increased production of LTC4, LTD4, and LTE4 contribute to eosinophilic and mast cell inflammation in the upper and lower respiratory tract.^20^, ^627^

Neurogenic type: Non-allergic rhinitis of the neurogenic type is caused by drug-induced modulation of the autonomic nervous system. Antihypertensives and vasodilators are among the many classes of drugs that cause drug-induced non-allergic rhinitis of the neurogenic type. Other non-specific drugs, such as psychotropics and immunosuppressants, have unknown direct mechanisms and are categorized as idiopathic, but they can also cause neuromodulatory effects of these same drugs. Examples are alpha and beta-adrenergic modulators, phosphodiesterase inhibitors, and angiotensin-converting enzyme inhibitors.^2^, ^627^

a) Alpha and beta-adrenergic modulators: They are indicated for various cardiovascular and respiratory diseases. The mechanism of action is due to the direct action of the drugs on the sympathetic and parasympathetic innervation, which influences nasal physiology during the use of these medications. Alpha and beta-adrenergic antagonists and presynaptic alpha agonists cause decreased sympathetic tone and parasympathetic stimulation, producing mucous edema, nasal congestion and rhinorrhea.^2^, ^627^
b) Phosphodiesterase Inhibitors (PDI): Prevent the enzymatic degradation of cyclic nucleotides. This inhibition has diverse effects, including smooth muscle relaxation, vasodilation, and bronchodilation, making these agents useful for the treatment of numerous diseases. PDE-3 and PDE-5 inhibitors are commonly used to treat intermittent claudication, heart failure, pulmonary hypertension, lower urinary tract symptoms, and erectile dysfunction. Nonselective PDE-3 inhibitors inhibit the hydrolysis of cyclic adenosine Monophosphate (cAMP), which eventually prevents platelet aggregation and encourages vasodilation with increased blood flow to the extremities.^2^, ^627^, ^628^
c) Angiotensin-Converting Enzyme Inhibitors (ACEIs): inhibit the conversion of angiotensin I to angiotensin II in the lungs and are commonly used for heart and kidney diseases. ACE inhibitors increase the production of bradykinin, an inflammatory peptide that causes vasodilation and smooth muscle contraction. Bradykinin B1 and B2 receptors have been demonstrated in the nasal mucosa, and application of bradykinin to the nasal mucosa has resulted in increased sneezing, as well as coughing, rhinorrhea, and nasal congestion.^2^, ^627^

### Drug-induced rhinitis

It is drug-induced and resulting from prolonged use of Topical Intranasal Decongestants (TIDs). Topical TIDs are readily available without a prescription and often lack appropriate warnings about prolonged use, which can result in overuse and dependence.^20^

Although there are no consensual diagnostic criteria, drug rhinitis was originally associated with the triad of prolonged use of TIDs, persistent nasal obstruction and rebound edema of the nasal mucosa. Patients experience nasal congestion, often without rhinorrhea or sneezing, and may notice reduced efficacy, or tachyphylaxis, with additional use of TIDs. The physical examination is variable, but often reveals edema, erythema and hyperemia of the nasal mucosa.^629^

Drug rhinitis can cause mucous edema, vasodilation and production of inflammatory mediators. Vasoconstriction and mucosal damage frequently accompany the use of these medications, and we cannot fail to emphasize this, especially in the use of illicit topical drugs. Drug-induced rhinitis differs from AR in that it is not induced by allergens or dependent on IgE mechanisms, although symptoms may be similar.^20^

Stimulation of α-adrenergic receptors results in vasoconstriction with a consequent increase in nasal permeability due to decreased blood flow and increased sinusoid emptying. The two most commonly used classes of nasal decongestants are imidazolines and sympathomimetic amines. Imidazolines are α-2 receptor agonists, while sympathomimetic amines stimulate the presynaptic release of norepinephrine. Norepinephrine stimulates α-adrenergic receptors and weakly stimulates β-adrenergic receptors. Both classes of medications are rapid-onset, potent, and long-lasting.^2^, ^629^

The exact pathophysiological mechanism that causes drug rhinitis is unclear, although there are several hypotheses:^20^ (1) Chronic vasoconstriction causes hypoxia and recurrent nasal tissue ischemia, which can cause interstitial edema; (2) Changes in endothelial permeability can result in increased edema; and (3) Continuous use of TIDs can decrease endogenous norepinephrine and negatively regulate α-receptors, through negative neural feedback, causing a decrease in adrenergic response.

Inflammatory cells, local inflammatory mediators, uninhibited parasympathetic stimulation, and increased mucus production also contribute to symptoms.

Histological changes in the mucosa after prolonged use of TIDs include ciliary injury and loss, epithelial cell injury, epithelial metaplasia and hyperplasia, dilated intercellular spaces, goblet cell hyperplasia, and edema.

Benzalkonium chloride, an antimicrobial preservative used in some nasal sprays, has been implicated in the mechanism of rhinitis drugs. Studies have shown that benzalkonium chloride is toxic to the nasal epithelium and induces mucous edema, propagating drug-induced rhinitis, although the data are inconclusive.^630^

Neither the duration nor the cumulative dose of TIDs required to initiate rhinitis drugs are known. Rebound congestion may develop after three to 10 days of taking the medication but may not occur until after 30 days. Other studies have demonstrated a lack of rebound congestion after eight weeks of continuous use. Furthermore, doubling the dose of intranasal imidazoline did not increase the extent of rebound edema. Although inconclusive, studies suggest that use of TIDs should be stopped after three days to prevent rebound congestion.^2^, ^629^

Treatment of drug-induced rhinitis, despite the lack of formal treatment guidelines, discontinuation of TIDs is essential. Patients should be educated about over-the-counter products containing decongestants, as information on packaging may be inadequate.^20^

Several treatments have been tested, including nasal cromolyn, nasal saline solutions, oral/intranasal antihistamines, steroid injections into the turbinates’, and oral/intranasal corticosteroids. Intranasal corticosteroids are the most common drug-treatment for rhinitis. Many start intranasal corticosteroids while reducing TID use.^625^, ^626^, ^631^

Often there is an undiagnosed underlying rhinitis and/or an anatomical problem that initiated decongestant use, and this must be addressed to alleviate the patient's impulse to use TIDs. For refractory cases, oral steroids and inferior turbinate reduction may be considered.^20^

Rhinitis drug is typically associated with repeated exposure to TIDs, with an increase in symptoms when the medication is discontinued. In contrast, AR is classically associated with an allergic trigger, with similar symptoms and increase following allergen exposure and depending on IgE-mediated inflammation. It is possible that both may coexist, and a careful history must be taken regarding these triggers to obtain an accurate diagnosis and provide appropriate treatment.^632^

### Idiopathic rhinitis

It is a chronic rhinopathy diagnosed by exclusion of other rhinitis. It can be described as a chronic non-inflammatory non-allergic rhinitis, defined by some authors as non-allergic rhinopathy.^633^ Clinical features include primary symptoms of nasal congestion and rhinorrhea, post-nasal drip, throat clearing, cough, Eustachian auditory tube obstruction, sneezing, hyposmia, facial pressure and headache. These symptoms may be perennial, persistent, or seasonal, and are typically triggered by defined stimuli such as cold air, weather changes, temperature changes, humidity, barometric pressure, strong odors, tobacco smoke, changes in sex hormone levels, environmental pollutants, physical exercise and alcohol. Notably, the lack of a defined trigger does not exclude the diagnosis of idiopathic rhinitis.^20^

The prevalence of idiopathic rhinitis, the second most common form of rhinopathy, is between 7% and 9.6% in the adult population in the United States and Europe and is found in 71% of cases, occurring with a female:male ratio of 2:1–3:1 and appears after 20 years of age. It is defined by the absence of an IgE-mediated immune response. The term idiopathic rhinitis has been suggested to replace vasomotor rhinopathy, as allergic inflammation is absent in the pathogenesis, and the vasomotor cause does not explain the pathogenesis of all cases.^20^, ^633^

The nasal mucosa of patients with idiopathic rhinitis may exhibit erythema and clear rhinorrhea. Allergy testing can be used to differentiate between idiopathic rhinitis and AR. Idiopathic rhinopathy has been associated with autonomic dysfunction and attributed to an imbalance between the parasympathetic and sympathetic systems.^20^, ^633^

Neurosensory abnormalities are considered important in the development of idiopathic rhinitis. In previous reviews of central responses to olfactory stimuli, individuals with idiopathic rhinitis underwent functional magnetic resonance imaging after exposure to different odors (vanilla and walnut smoke). Findings included increased blood flow to the olfactory cortex, leading to the hypothesis of an altered neurological response.^20^, ^633^

Clinical treatment of idiopathic rhinitis includes topical nasal sprays with variable responses, used alone or in combination. The most commonly used are IC, topical azelastine and ipratropium bromide. Additionally, additional treatments include saline nasal sprays or washes, especially when there is significant postnasal drip.^624^, ^626^

For symptomatic patient’s refractory to medical treatment, surgical approaches targeting the Vidian nerve, and its branches have shown symptom control. These include botulinum toxin injections, endoscopic vidian neurectomy, endoscopic posterior nasal neurectomy, and posterior nasal nerve cryosurgery. We must emphasize that these procedures are not free from undesirable adverse effects. Posterior nasal neurectomy is associated with lower rates of dry eye complications than vidian neurectomy. Recent studies show that in-office cryotherapy can achieve improvement in rhinorrhea and congestion for up to 1 year.^20^

### Irritant rhinitis

Also known as chemical rhinitis, it occurs due to exposure of the nasal mucosa to irritating agents, or irritating chemicals. Exposures to chemicals and environmental pollutants increase every day, and patients may experience rhinitis symptoms that do not necessarily fit a traditional allergy profile.^20^

Chemicals can cause neural irritation, generating typical symptoms of irritation, such as congestion, sneezing, runny nose, nasal discomfort, post-nasal drainage, headache, olfactory dysfunction and epistaxis, and conjunctival irritation. These upper symptoms may be associated with lower airway symptoms. The diagnostic differentiation of irritative rhinitis is wide, including occupational rhinitis, but not all irritative rhinitis is occupational. Typically, differentiation should include causes of both AR and non-allergic rhinitis, as well as mixed rhinitis, recurrent acute rhinosinusitis, and chronic rhinosinusitis.^20^

Exposures at home and at work are essential elements to obtain in the clinical history. There are many chemicals with which specific occupations are strongly associated, and household chemicals may also play a role.^20^

Volatile organic compounds such as benzene, toluene and the secondary production of formaldehyde can be found in cleaning products, furniture, plastics, flooring and can cause barrier dysfunction and inflammation in both the upper and lower airway. Chemicals known to cause respiratory inflammation and, in some cases, allergic sensitization, include diisocyanatos, acid anhydrides, some platinum salts, reactive dyes, and many cleaning products used in hospitals and in the pandemic era, including glutaraldehyde, quaternary ammonium compounds and chloramine. In general, asking about exposure to vapors, smoke, and dust can be helpful in determining whether a patient has contact with irritants.^634^

Larger chemical particles with a diameter greater than 10 μm are deposited in the upper airway and agents such as ammonia, formaldehyde, nitrogen dioxide or sulfur dioxide, among others, can easily alter the respiratory epithelial barrier.^20^ These changes are generally not mediated by IgE, but by a reflex response often called neurogenic inflammation. A subset of these individuals involved in single exposure incidents may develop persistent and chronic symptoms. This phenomenon has been described as reactive upper airway dysfunction syndrome when only rhinitis symptoms are present and reactive airway dysfunction syndrome when asthma-like symptoms are present.^20^

There is still debate about the exact mechanism behind sensitization to these chemicals. However, smaller chemical compounds must associate with larger protein molecules to induce an immune response. As a result, assessment of sensitization through skin testing and/or IgE assessment may be useful and, in the future, immunoassays based on cellular responses may serve as better biomarkers of chemical exposure.^20^

The treatment aims at environmental hygiene, avoiding irritating and/or chemical agents. The use of nasal lavage helps to clean the nasal mucosa and consequently reduce contact with agents. ICs are indicated for their potent anti-inflammatory action on the nasal mucosa.^626^

### Gustatory and food-associated rhinitis

It is characterized by watery rhinorrhea, unilateral and/or bilateral, a few minutes after ingesting food, spicy foods such as peppers and others that contain capsaicin. Rhinorrhea lasts as long as food is ingested.^20^

Gustatory rhinitis may be mistakenly confused with IgE-mediated allergy, but there is no sneezing, itching, or facial pain, and the course of rhinorrhea is self-limiting. There is no associated disturbance of smell or taste. Gustatory rhinitis occurs more frequently in patients with AR and patients who have a history of smoking, but not in those with asthma or food allergies.^20^, ^635^

Its pathophysiology was confirmed by pharmacological observations and immunohistology studies, where symptoms occur due to activation of a neural reflex arc initiated after stimulation of afferent sensory nerves. This leads to stimulation of the parasympathetic efferent nervous system to the submucosal glands of the nasal mucosa. It is also possible that interactions between the sympathetic and parasympathetic nervous systems may lead to uninhibited activity of the parasympathetic system with resultant rhinorrhea. E.g., the chemical capsaicin is known to cause gustatory rhinitis. The capsaicin receptor is a Transient Receptor Potential Vanilloid subtype 1 (TRPV1) present in neuronal and non-neuronal cells throughout the nasal mucosa and oral epithelium. A direct effect on goblet cell secretion can be triggered when capsaicin is ingested.^635^, ^636^

The treatment of gustatory rhinitis consists of avoiding the triggering food. Topical anticholinergic medications, such as ipratropium bromide, are used when contact occurs.^625^ The use of topical capsaicin and Posterior Nasal Nerve (PNN) resection have been proposed as a last resort for intractable gustatory rhinitis.^635^

### Alcohol-induced rhinitis

Exacerbation of respiratory symptoms after alcohol ingestion occurs in 3%–4% of the general population. Among nasal symptoms, obstruction is the most common and can be accompanied by rhinorrhea, sneezing and lower airway symptoms. This is reported in patients with AR, asthma, chronic obstructive pulmonary disease, and emphysema. Up to 75% of patients with AERD experience exacerbations of respiratory symptoms when they consume alcohol. Symptom exacerbations occur soon after alcohol ingestion, are often associated with ingestion of small volumes, and appear to correlate with peak blood alcohol levels. Such symptoms can appear regardless of the type of alcohol consumed. These reactions to alcohol consumption are more prevalent in patients with chronic rhinosinusitis with nasal polyps who suffer from severe and recurrent disease and are related to the severity of upper airway inflammation. In patients with AERD, the severity of aspirin-induced respiratory symptoms is positively related to the severity of alcohol-induced reactions. Exacerbations of respiratory symptoms in response to alcohol have been shown to be decreased after aspirin desensitization in patients with AERD. Patients with AERD have elevated baseline levels of cysteine leukotrienes, which are proposed to mediate upper and lower airway reactions to aspirin. Cardet and colleagues propose that cysteine leukotrienes are also mediators of the response to alcohol in these patients, although this mechanism is not yet fully understood. High alcohol consumption is observationally and genetically associated with high serum IgE levels, although not with allergic disease.^636^, ^637^, ^638^

Two possible mechanisms have been proposed as the etiology for this observation:^636^, ^637^ (1) Alcohol shifts the balance of Th1 and Th2 responses toward a Th2 immune response with a direct effect on B cells; (2) Alcohol induces increased uptake of endotoxins from the intestine, resulting in elevated levels of IgE.

### Hormonal and gestational

Hormonal changes that occur during the menstrual cycle, puberty, pregnancy and menopause have been considered as potential triggers of non-allergic rhinitis. Although the association between certain endocrine conditions, such as hypothyroidism and acromegaly, and this subtype of rhinitis is classic, the evidence in favor of this link is still limited.^638^

The pathophysiology of hormonal rhinitis is not yet completely understood, but some of the mechanisms by which different hormones act are known. Estrogens, by inhibiting the activity of acetylcholinesterase, increase the production of acetylcholine by the parasympathetic nervous system, resulting in vascular engorgement that manifests as nasal obstruction and/or rhinorrhea. Both beta estradiol and progesterone not only increase the expression of histamine H1 receptors in the nasal epithelium and microvascular endothelial cells, but also play a role in the migration and/or degranulation of eosinophils.^639^ On the other hand, testosterone decreases the activation and eosinophil survival.^638^

The most generic form of hormonal rhinitis is known as “pregnancy rhinitis” or “vasomotor rhinitis of pregnancy”. This condition is characterized by the presence of rhinitis without an identified cause such as allergic, infectious or medication-related, and presents with watery rhinorrhea and nasal congestion as predominant symptoms.^638^

The symptoms present in the patient are edema of the nasal mucosa, congestion and sneezing and the optimization of local vasodilation due to the increase in circulating blood volume. Such manifestations remain for a period of six weeks or more during pregnancy and disappear completely within two weeks after the birth.^638^

The diagnosis of this form of rhinitis is clinical, while investigation of the causes can be delayed in most cases until the postpartum period.^638^

Pregnant rhinitis in its milder forms can be treated with non-pharmacological measures, such as physical exercise, elevating the head of the bed and nasal irrigation with saline solution. However, in moderate or severe cases, it may be necessary to use courses of nasal corticosteroids or apply nasal decongestants for short periods.^638^

### Emotional rhinitis

Emotional rhinitis is a condition that manifests itself in susceptible individuals in times of stress, such as psychological, physical, intellectual and emotional distress. This disorder can also appear in other circumstances, such as during sexual intercourse, due to stimulation of the parasympathetic autonomic nervous system. Psychiatric disorders can resemble medical conditions such as asthma, stridulous laryngitis, and even panic syndrome.^19^

The pathophysiology of these symptoms may be related to a response from the autonomic nervous system, specifically to the activation of the parasympathetic branch, which can cause vasodilation and increased production of nasal mucus.^19^

In situations of emotional stress, the body can release chemicals such as histamine and other inflammatory mediators, which can lead to inflammation of the nasal mucous membranes and nasal congestion. Additionally, emotional stress can increase the central nervous system's sensitivity to perceptions of nasal discomfort, making symptoms even more prominent.^19^

The main symptom is nasal obstruction due to mucosal congestion. Other symptoms may include a clear nose, problems with smell, anxiety and depression.^19^

Diagnosis is usually made by complete medical evaluation, which may include a detailed medical history, physical examination and, in some cases, additional tests such as allergy tests or sinus imaging studies.^19^

If there is no identifiable underlying medical cause for nasal symptoms associated with emotions, treatment may focus on managing emotional distress and taking steps to relieve nasal congestion, such as nasal rinsing with saline, using nasal decongestants, or in some cases, cognitive behavioral therapy to address the emotional component of symptoms.^19^

The objective of the treatment is to promote a better balance between work and leisure, incorporating physical exercises and activities to relax and improve self-esteem. In some cases, the use of appropriate medications and psychiatric counseling may be necessary.^19^

### Ozonous atrophic rhinitis

Atrophic rhinitis is characterized by the progressive loss of the upper airway lining, accompanied by the formation of nasal crusts, bone destruction, mucosal atrophy and paradoxical nasal congestion.^638^

In the pathophysiology of atrophic rhinitis, histological changes are noted that include the transformation of pseudostratified columnar epithelium into squamous epithelium, changes in blood vessels, loss of sensitivity, loss of goblet cells and mucus-producing cells. This leads to stagnation and a poor mucociliary barrier, often resulting in colonization by pathogenic organisms.^638^, ^639^

Clinically, atrophic rhinitis presents with nasal obstruction, purulent posterior rhinorrhea, crusting, epistaxis, loss of smell and sensation of facial pressure. It is common for it to be associated with sinusitis. There are two specific clinical presentations identified: the dry form, which occurs in long-lasting cases, characterized by nasal dryness and pale and atrophic nasal mucosa, with adhesion of crusts; the wet form, which occurs during active inflammation and is characterized by the presence of mucopurulent rhinorrhea. It is common for these two forms to coexist in the nasal mucosa, although there is a tendency for them to evolve into the dry form.^637^

Presentation at rhinoscopy may vary depending on the duration of the disease and the presence of inflammation or active technology employed. In terms of imaging, it is common to observe thickening of the paranasal sinuses lining, loss of definition of the ostiomeatal complex, hypoplasia of the maxillary sinus, enlargement of the nasal cavities and destruction of the inferior and middle turbinates.^638^

The diagnosis is established by clinical criteria, including the presence of at least two of the following symptoms: epistaxis, anosmia, purulent rhinorrhea, formation of nasal crusts, chronic inflammatory disease of the airway and two or more nasal surgeries, lasting at least six months. It can be confirmed by biopsy of the nasal mucosa and imaging tests.^640^

Treatment of atrophic rhinitis aims to control symptoms and involves maintaining nasal hygiene with regular use of saline solutions and nasal hydration. Topical or systemic antibiotic therapy may be necessary in cases of purulent secretions, as indicated by microbiological results. In patients with conventional response to medical treatment, surgical therapy may be considered.^638^

### Secondary atrophic rhinitis

Secondary Atrophic Rhinitis (SAR) is the most prevalent form of atrophic rhinitis in developed countries, being clinically distinct from the primary form. This is frequently observed in clinics specializing in otorhinolaryngology and immune allergology. SAR is less severe than primary atrophic rhinitis, resulting in less impact on patients' quality of life.^641^

The association of SAR with chronic nasal inflammation or other forms of injury to the nasal mucosa (such as trauma, surgery, antiangiogenic therapies, among others) suggests that the development of SAR is a common consequence resulting from various forms of aggression to the nasal mucosa. Histopathological studies have shown that in SAR there is gradual loss of the nasal ciliated pseudostratified columnar epithelium, with metaplasia to a squamous epithelium devoid of cilia, loss of goblet cells and of mucus-producing cells.^641^

Therefore, the pathophysiology of SAR is believed to be based on the change of normal respiratory epithelium, with transition to a non-ciliated squamous epithelium, which reduces mucociliary clearance capacity and leads to the subsequent accumulation of stagnant mucus. These changes create a favorable environment for chronic bacterial superinfection.^641^

The main symptoms of SAR may include persistent nasal obstruction; nasal discharge, usually thick and with an unpleasant odor; decreased sense of smell (hyposmia) or changes in the sense of smell (parosmia or cacosmia); dry or moist crusts inside the nose; feeling of nasal dryness; occasional epistaxis (nosebleed); feeling of nasal congestion, even without evidence of mucus and chronic coughing, especially at night, due to post-nasal drainage.^641^

Diagnosis of SAR usually involves a complete clinical evaluation, which may include:^641^

• Clinical history: The doctor will ask about the symptoms the patient is experiencing, their duration and severity, as well as any triggering or aggravating factors;

• Physical examination: The doctor will examine the inside of the nose using an otoscope or nasal endoscope to evaluate the nasal mucosa, looking for signs of atrophy, crusts, secretions or other changes;

• Assessment of smell: Smell tests can be performed to assess the patient's olfactory function, as loss or alteration of smell is common in secondary atrophic rhinitis, and
• Complementary tests: In some cases, additional tests may be necessary to confirm the diagnosis or evaluate complications. This may include allergy testing, nasal secretion cultures to identify bacterial or fungal infections, or sinus imaging to assess the extent of nasal changes.

SAR treatment aims to alleviate symptoms, reduce complications and improve the patient's quality of life. Therapeutic approaches may include:^641^

• Nasal hydration: The use of saline solutions for nasal irrigation can help moisten and clean the nasal passages, reducing the formation of crusts and improving nasal breathing;
• Nasal lubrication: The use of nasal gels or ointments based on mineral oil or other lubricating agents can help relieve the sensation of nasal dryness and reduce discomfort;
• Topical medications: Using topical nasal corticosteroids can help reduce nasal inflammation and relieve symptoms. In some cases, the doctor may prescribe topical antibiotics to treat secondary infections;
• Treatment of infection: If there is evidence of bacterial or fungal infection, treatment with antibiotics or antifungals may be necessary;
• Vitamin and mineral supplementation: In some cases, supplementation with vitamins and minerals, such as vitamin A, zinc and omega-3 fatty acids, can help promote the health of the nasal mucosa;
• Avoid nasal irritants: Avoiding factors that can irritate the nasal mucosa, such as cigarette smoke, air pollution and irritating chemicals, can help reduce symptoms; and
• Medical follow-up: It is important to have regular follow-up with a doctor specializing in otolaryngology to monitor the response to treatment, adjust as necessary and detect and treat complications early.

### Rhinitis secondary to structural anatomical variations

Rhinitis secondary to structural anatomical variations refers to a form of rhinitis that is caused by specific anatomical characteristics of the nose and paranasal sinuses. These anatomical variations may include deviated nasal septum, enlarged turbinates, nasal polyps or narrowing of the nasal passages.^642^

The pathophysiology of rhinitis secondary to structural anatomical variations involves a complex interaction between the anatomical characteristics of the nose and paranasal sinuses, and the physiological processes that occur in these structures. These anatomical variations can interfere with normal airflow through the nostrils, resulting in nasal obstruction, difficulty breathing, and chronic inflammation of the nasal mucosa. Additionally, obstruction of the nasal passages can predispose to secondary infection and chronic inflammation, contributing to persistent rhinitis symptoms.^642^

Symptoms of rhinitis secondary to structural anatomical variations may be similar to those of allergic rhinitis and include nasal congestion; runny nose; difficulty breathing; facial pressure; sneezing; itchy nose; loss of smell; snoring; dry mouth; chronic cough and sleep apnea.^642^

The diagnosis of rhinitis secondary to structural anatomical variations involves a comprehensive medical evaluation, which may include:^642^

• Clinical history and physical examination: The doctor will review the patient's symptoms, including the nature of the nasal obstruction, the presence of congestion, runny nose, facial pain, and breathing difficulties. The physical examination may include inspection of the nostrils and palpation of the nasal region and paranasal sinuses in search of signs of inflammation or anatomical variations;
• Nasal endoscopy: This procedure enables the doctor to directly examine the inside of the nose and sinuses using a flexible endoscope. This may reveal the presence of nasal polyps, deviated septum or other anatomical variations that contribute to rhinitis symptoms;
• Computed tomography of the paranasal sinuses: This exam may be requested to evaluate the nasal and paranasal sinus structures in more detail, especially if significant anatomical anomalies are suspected;
• Allergy testing: Although rhinitis secondary to structural anatomical variations is not caused by allergies, some patients may have concomitant allergies that exacerbate symptoms. Therefore, allergy testing can be performed to determine whether allergens are contributing to symptoms; and
• Nasal sensitivity assessment: In some cases, doctors may perform nasal sensitivity tests to evaluate the response of the nasal passages to different stimuli, such as chemical irritants or changes in air temperature and humidity.

Based on the results of these tests and general clinical assessment, the doctor can diagnose rhinitis secondary to structural anatomical variations and recommend an appropriate treatment plan, which may include conservative measures, medication and, in some cases, surgical intervention to correct the underlying anatomical variations.

Treatment of rhinitis secondary to structural anatomical variations generally involves a multifaceted approach, which may include:^642^

• Medication: Taking medication can help relieve symptoms such as nasal congestion and inflammation. This may include nasal decongestants, antihistamines, nasal corticosteroids, and saline nasal sprays for irrigation;
• Treatment of anatomical variations: In cases where anatomical variations, such as a deviated nasal septum, enlarged turbinates or nasal polyps, are causing significant nasal obstruction, surgical correction may be necessary. This may involve procedures such as septoplasty (correction of a deviated septum), turbinectomy (removal of part of the nasal turbinate) or polypectomy (removal of nasal polyps);
• Complementary therapy: In addition to medication and surgery, some people may benefit from complementary therapies, such as saline nasal irrigation, which can help clear the nasal passages and reduce congestion. Breathing therapies and relaxation techniques can also be helpful in relieving symptoms; and
• Medical follow-up: It is important to have regular follow-up with a doctor to monitor treatment effectiveness and adjust as necessary. If symptoms persist or worsen, it may be necessary to reevaluate the diagnosis and consider other treatment options.

## Special considerations

### Local allergic rhinitis

Over the last few decades, studies have demonstrated that a considerable number of patients with rhinitis and negative responses to systemic sensitization tests present an exclusively nasal allergic inflammatory response,^643^ confirmed by the specific Nasal Provocation Test (sNPT) with pollens and/or dust mites.^644^, ^645^ Based on these findings, the concept of a new rhinitis phenotype was created, called Local Allergic Rhinitis (LAR).^646^

In adults, LAR is a stable phenotype^647^ that predominantly affects young, healthy, non-smoking women with a family history of atopy, in addition to being associated with conjunctivitis and asthma.^648^ It affects around 25% of the population with rhinitis and may correspond to Up to 50% of adult patients previously diagnosed with Non-Allergic Rhinitis (NAR), approximately one third of whom present with onset of symptoms in childhood.^649^

We know that although the inflammatory response is only local, this phenotype does not represent an initial or transitional stage for Allergic Rhinitis (AR)^650^ and presents a severity similar to that of other rhinitis phenotypes, including those in the pediatric range. Although there are few studies in this age group, a recent review^651^ demonstrated a wide variation in the prevalence rates of LAR (3.7%–83.3%) in patients previously classified as NAR. Markedly lower rates were found in Eastern countries (3.7%–16.6%), when compared to Western countries (22.3%–83.3%), but without identifying relevant clinical characteristics that could explain this discrepancy or differentiate between the different phenotypes of childhood rhinitis.

After a positive sNPT, patients with LAR present local production of IgE, immediate activation of mast cells and eosinophils with local release of pro-inflammatory mediators such as tryptase and eosinophilic cationic protein.^652^ The presence of local IgE also occurs during periods of non-exposure to the allergen, demonstrating persistent local production of the antibody. Several possible mechanisms to explain the local production of IgE have already been reported;^652^ however, it is still unknown whether there are differentiating factors in the local synthesis of IgE in patients with AR and LAR.^652^ Despite these pathophysiological changes, to date, due to limitations in carrying out specific IgE measurement in nasal secretion, this is not yet considered essential for the diagnosis of LAR. Therefore, sNPT continues to be the diagnostic gold standard for this condition.^64^, ^298^

Regarding treatment, as in AR, LAR symptoms tend to decrease with the use of topical nasal corticosteroids and administration of systemic H1 anti-H1s.^648^, ^653^ Another possible therapeutic approach would be the institution of specific immunotherapy, since this type of treatment has already demonstrated effectiveness in adults.^653^, ^654^, ^655^

Thus, it is possible to state that LAR presents a frequency and intensity of symptoms similar to other rhinitis phenotypes in all age groups, and differentiation between them is only possible by monitoring nasal responses after performing sNPT.^20^

### Mixed rhinitis

Mixed rhinitis can be considered in those patients who present symptoms both after exposure to allergens and after non-specific stimuli (chemical or physical), that is, they present an overlap of allergic rhinitis and different phenotypes of non-allergic rhinitis. In mixed rhinitis, in addition to chronic allergic inflammation, other neurovascular mechanisms contribute to nasal hyperreactivity^273^ and the consequent triggering of symptoms after exposure to nonspecific stimuli. It is estimated that around 30%–50% of patients with chronic rhinitis present this overlap in diagnoses,^25^ and clinical history, the presence of subjective symptoms and systemic sensitization tests are essential to correctly make the diagnosis.

### Dual rhinitis

**Fig. 5:**
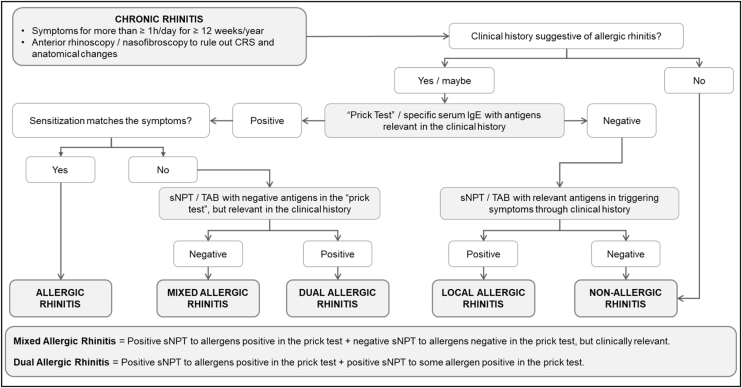
Chronic rhinitis diagnostic algorithm. Prick Test, Immediate Hypersensitivity Skin test; CRS, Chronic Rhinosinusitis; sNPT, Specific Nasal Provocation Test; TAB, Basophil Activation Test. Adapted from Eguiluz-Gracia I.^656^

Recent evidence suggests that local and systemic sensitization to different aeroallergens can coexist in the same patient. This phenotype is described as dual rhinitis (concomitant presence of AR and LAR) and can be diagnosed, for example, in patients who present perennial symptoms with seasonal exacerbation, being systemically sensitized only to seasonal allergens, but who, when subjected to sNPT, present results positive also for perennial allergens.^23^ Its relevance has been demonstrated in both adults and children and its diagnosis should be considered in atopic individuals with systemic sensitization to a certain aeroallergen, but who do not present adequate clinical-laboratory correlation to the pattern of nasal symptoms reported after exposure to it.^24^ Fig. 5 shows the approach to the differential diagnosis of the various types of chronic rhinitis

### Children

The term rhinitis in the pediatric age group has some particularities that make the infectious aspects stand out, with viral infections being the most common causes of nasal symptoms in the first two years of life, due to greater immunological immaturity and greater contact in daycare centers and schools.

Symptoms of obstruction and secretion in children may be due to other differential diagnoses including: anatomical changes such as cleft palate, unilateral choanal stenosis or atresia, adenoid enlargement, laryngopharyngeal reflux, ciliary dyskinesia, presence of polyps that lead us to the diagnosis of cystic fibrosis, and even a foreign body retained in one of the nasal passages with unilateral purulent secretion.^657^ There may be coexistence of infectious rhinitis with AR, which often makes the initial diagnosis of AR difficult.

If we consider the progression of the atopic march and allergic sensitization, the characteristic signs and symptoms of Allergic Rhinitis (AR) become more frequent with high prevalence of AR in adolescence and young adults.^393^

The cohort study in Germany (Multicenter Allergy Study ‒ MAS) provided evidence that sensitization for at least two consecutive years to aeroallergens, especially pollens, increased the prevalence of new cases of AR between three and 12 years of age by 2% per year.^240^, ^658^

Typical symptoms of AR: sneezing, anterior and/or posterior secretion, nasal obstruction and itching become more frequent with age and may be associated with snoring, throat clearing, coughing, oral breathing, eye itching, infraorbital darkening, elongated and pale facies, dental malocclusion, arched and high palate, double infraorbital fold (Dennie-Morgan lines), transverse nasal groove, in addition to the presence of other signs and symptoms of allergic diseases such as atopic dermatitis and asthma.^476^ Anterior rhinoscopy may show hypertrophy and pallor of turbinates in addition to clear secretion, independent of viral symptoms.

The diagnosis of AR is based on clinical history and tests that can identify allergic sensitization due to the presence of specific IgE, especially to mites, domestic animals, cockroaches and pollens in areas where pollination exists. Rarely, food allergies alone cause AR symptoms. The search for nasal eosinophils is not performed routinely, in addition to being difficult to obtain and analyze due to the high number of viral infections in this age group.

Other rhinitis endotypes such as idiopathic rhinitis and non-allergic eosinophilic rhinitis are less common in childhood, but Local AR (LAR) has been demonstrated in up to 47% of pediatric cases previously diagnosed as non-allergic rhinitis. Some authors associate environmental changes such as temperature, humidity and greater exposure to pollutants as risk factors for LAR.^20^, ^659^, ^660^, ^661^, ^662^, ^663^, ^664^, ^665^, ^666^, ^667^, ^668^, ^669^, ^670^, ^671^

All of these symptoms may be underappreciated in children and often lead to changes in sleep, sleep apnea, fatigue, irritability in the morning, reduced school performance, interfering with the quality of life of the child and their families.

Treatment before the age of two is limited and often only includes the use of second-generation antihistamines approved for that age range. Intranasal corticosteroids such as mometasone furoate, triamcinolone acetonide and fluticasone furoate can now be used in preschool children. In indicated cases, allergen immunotherapy can be useful and even reduce the risk of progression to respiratory symptoms such as asthma.^476^

### Elderly

Population aging has resulted in an increase in the prevalence of chronic diseases in the elderly. The prevalence of nasal symptoms, including rhinorrhea, congestion, sneezing, nasal/ocular itching and post-nasal drainage affects up to 32% of elderly people and can have a significant impact on quality of life. Several factors associated with aging may contribute to the pathogenesis of rhinitis in the elderly.^662^, ^663^

The physiological aging process causes changes in nasal anatomy and physiology that impact the presence and worsening of rhinitis symptoms. The weakening of connective tissue and atrophy of collagen fibers in the nasal cartilages cause a decrease in nasal flow and symptoms of nasal congestion. Changes also occur in the nasal epithelium, which becomes atrophic and dry. The decrease in nasal flow contributes to the reduction of humidification and heating of the inspired air, leading to dryness, crusting and nasal irritation. Nasal mucus changes and becomes thicker, which, associated with a decrease in mucociliary function, causes increased nasal drainage, post-nasal drainage and coughing. Aging is also associated with increased cholinergic activity and reduced olfaction. Furthermore, immunosenescence also contributes to rhinitis symptoms, increasing vulnerability to infectious rhinitis.^662^

It is important in the elderly, especially in cases of late-onset AR, to conduct the diagnosis carefully and consider several conditions in the differential diagnosis, including non-allergic rhinitis, hormonal rhinitis, drug-induced rhinitis, atrophic rhinitis, inflammatory immunological disorders (e.g., granulomatous diseases, amyloidosis), nasal leakage of cerebrospinal fluid and malignancies in the nasopharynx. The elderly due to several comorbidities, are subject to “polypharmacy” and several medications widely used in this age group cause or contribute to the worsening of rhinitis, such as non-steroidal anti-inflammatory drugs, alpha-blockers, beta-blockers, ACE inhibitors, calcium channel blockers, diuretics, oxymetazoline, phosphodiesterase inhibitors and psychotropic drugs.

Comorbidities of special interest include depression, which is associated with anosmia, rhinitis and chronic rhinosinusitis disease; gastroesophageal reflux disease, with a strong association with the development of rhinitis; and sleep apnea syndrome, with a high prevalence in the elderly. It is important to highlight that several psychiatric medications can cause dry nasal mucosa and rhinitis.^662^, ^664^, ^665^

The treatment of AR in the elderly is carried out in an analogous way to that in the adult population, with intranasal corticosteroids, antihistamines (topical and oral) and immunotherapy with allergens. In cases of rhinorrhea associated with age and idiopathic rhinitis, the recommended therapy is nasal lavage with saline solution and topical anticholinergics. It is important to highlight that the elderly may be more susceptible to adverse events from some medications. First-generation antihistamines should be avoided due to the risk of adverse events, especially urinary retention, dry eyes/mouth, constipation and arrhythmias. Intranasal corticosteroids are well tolerated, although there is a theoretical risk of osteoporosis with high doses for a prolonged period of time. The patient must be monitored for glaucoma, especially if they are simultaneously using inhaled corticosteroids or taking courses of oral corticosteroid therapy. The impact of intranasal corticosteroids on the risk of cataracts is not clear and defined.^20^, ^662^, ^666^

Finally, it is important to remember that treatment compliance may be compromised by memory loss, cognitive decline and motor changes. It is estimated that memory loss affects 37% of elderly people over 85 years of age. Considering this aspect, it is recommended to adopt strategies to maximize compliance, which include the following: prescribing the smallest number of medications possible, adjusting the dosage schedule to the patient's daily habits and coordinating the administration of all medications for the patient(s), whenever feasible. In addition to memory loss, other factors can impair compliance, related to low motor coordination, such as Parkinson's disease, stroke sequelae, arthritis and weakness in the hands, which can compromise the ability to use nasal sprays.^662^

### Pregnant and nursing mothers

Gestational rhinitis affects around 20% of pregnant women and it is defined as a nasal obstruction that typically occurs in the second or third trimester of pregnancy in women with no history of rhinitis prior to pregnancy. This nasal obstruction lasts for six weeks and has no allergic cause or signs of upper airway infection. Symptoms cease two weeks after delivery.

The pathogenesis suggests that placental trophoblastic hormone can induce hypertrophy of the nasal mucosa and progesterone increases the expression of H2 receptors in the nasal epithelium and microcirculation endothelium, promoting eosinophilic migration and degranulation.^31^, ^639^

The diagnosis is clinical, characterized by nasal obstruction. Mouth breathing may be associated with worsening sleep quality with snoring and obstructive apnea.^639^

In treatment, non-pharmacological measures must be implemented, such as nasal washing with saline solution, nasal dilators, postural guidance and physical exercises. Regarding the use of medications, FDA regulations for pregnant women must be followed, observing risks from A to D, as well as category X. In 2015, new rules describing potential drug effects on pregnancy and lactation were implemented.

During pregnancy, topical nasal corticosteroids are considered safe, including fluticasone, mometasone and budesonide. As for oral medications, notably in the first trimester, they are related to the risk of increased cleft lip, with or without cleft palate.

Second generation antihistamines are the most recommended (e.g., loratadine, cetirizine, fexofenadine, azelastine). First generation drugs should be avoided due to their anticholinergic effects.

Intranasal chromones have no contraindication, as they are not absorbed by the nasal mucosa (category B).^667^

Oral decongestants should be avoided in the first trimester, due to the risk of congenital malformations.^667^

Few antihistamines have been studied in nursing mothers, trying to evaluate their transfer into breast milk and possible adverse effects on infants. Second generation antihistamines such as loratadine and cetirizine are the most recommended, with lower transfer levels and a higher safety profile.

Newborns, particularly premature ones, eliminate medications more slowly, due to liver and kidney immaturity; therefore, the half-life of the medication and the length of time the nursing mother uses the medication must always be considered.^668^

### Athletes

Rhinitis is the most common nasal condition in athletes, with an estimated prevalence between 27% and 74%.^669^ This wide variation occurs due to differences between the populations studied and/or the diagnostic criteria used. Systematic review that evaluated the prevalence of rhinitis in athletes, according to the sport environment (land, water and cold air), concluded that swimmers are the most affected population (40%–74%), followed by snow skiers (46%) and track athletes (21%–49%).^670^

The acute effects of exercise on the nose are well defined – vasoconstriction of the capacitance vessels, resulting in an increase in nasal volume. Under normal circumstances, there is no rebound effect and vasoconstriction lasts for about one hour after exercise. The impact on nasal physiology of continuous physical training is not well established. Several environments in which athletes practice their physical activities have the potential to harm the nasal mucosa. For example, sports that are practiced in environments with chilly air (snow skiing, snowboarders and ice hockey) or in chlorinated water (swimming, diving and water polo) expose the nasal mucosa to local chemical and physical irritants. Outdoor aerobic exercise may also result in inhalation of large volumes of aeroallergens or air pollutants due to the increased minute ventilation required to support intense physical activity.^670^

Physical exercise can be a trigger or aggravating factor for rhinitis. Exercise-Induced Rhinitis (EIR) has been identified with progressive frequency and it is characterized by rhinorrhea, congestion and sneezing during intense exercise such as running, cycling and winter sports. However, the underlying pathophysiology remains unclear. EIR in athletes with and without allergies is associated with aggression of the nasal epithelium, influx of neutrophils, increased release of vasoactive mediators (histamine and leukotrienes) and reduced mucociliary clearance, but the trigger for the release of these inflammatory markers in athletes remains undefined.^671^, ^672^ However, it has been reported that EIR is more common among elite athletes and high-level athletes compared to individuals who participate in recreational sports, which suggests that the pathophysiology may be related to increased oxygen demand and the need corresponding to greater inspiratory air flow.^673^

Athlete performance requires optimal levels of health and fitness. Rhinitis has a negative impact on an athletes' sleep, well-being, performance and quality of life. Furthermore, rhinitis has a strong association with bronchial hyperresponsiveness and asthma, implying potential impairment of the athlete's lung capacity. Sleep impairment negatively affects athletes' performance, including deficits in aerobic capacity, isometric strength, cortisol levels, and sprinting.^674^

Doctors who care for athletes with rhinitis, at any level of competition, must take several factors into consideration when prescribing therapy; including the profile of adverse events, effectiveness for the preponderant symptom(s), individual response, route of administration, costs and restrictions imposed by the World Anti-Doping Agency (WADA) regarding the use of medications during competitions (Table 15). Special attention should be given to nasal decongestants and oral corticosteroids.^674^

**Table 15:** Therapeutic options for the treatment of rhinitis in athletes and the restrictions established by the World Anti-Doping Agency (WADA).^674^, ^675^

Medication	WADA rules	Remarks
Saline solution	Allowed	Widely available and of low cost.
Oral and nasal antihistamine agents	Allowed	Choose second Generation anti-H1 with less sedative effects.
Decongestants	Allowed: phenylephrine, phenylpropanolamine, synephrine and oxymetazoline	Mainly indicated for rhinosinusitis.
		No indication for allergic and non-allergic rhinitis.
	Allowed with limited urinary concentrations:	
	Cathine <5 mcg/mL	
	Ephedrine <10 mcg/mL	
	Pseudoephedrine <150 mcg/mL	
Intranasal corticosteroids	Allowed	First line of treatment for persistent and/or moderate/severe allergic rhinitis
Oral corticosteroids	Allowed in competitions only with exemption for therapeutic use (Therapeutic use exemption ‒ TUE)	Use indicated only for severe allergic rhinitis resistant to standard therapy
Disodium cromoglycate	Allowed	Effective mainly for histamine-induced symptoms (itching, sneezing and rhinorrhea)
Montelukast	Allowed	Risk of psychiatric adverse events (anxiety, depression)
Intranasal anticholinergics	Allowed	Effective for histamine-induced symptoms (itching, sneezing and rhinorrhea)

## Surgical treatment

Surgery is recommended, in particular, for patients with nasal obstruction refractory to drug treatment and those with inferior turbinate hypertrophy.^20^, ^573^, ^676^, ^677^ The following surgical treatment options are considered: septoplasty/rhinoseptoplasty for patients with obstructive deviation of the nasal septum. In patients with significant and persistent rhinorrhea, resistant to clinical treatment, treatment options include vidian neurectomy or posterior nasal neurectomy, and cryoablation or radiofrequency of the posterior nasal nerves.^20^

Many patients require a combination of surgeries, such as septoplasty and turbinectomy.^678^, ^679^ Various surgical techniques are used, from simpler procedures, such as submucosal cauterization associated with lateral dislocation of the inferior turbinate, to procedures with more sophisticated equipment, such as radiofrequency, coblation, laser and microdebrider.^680^ However, there are few randomized comparative studies, with longer follow-up, that allow defining a technique as the gold standard for nasal turbinate surgery.^680^ From a practical point of view, experience of the surgeon, the available materials, the cost, and the local anatomy of the nose, end up defining the technique to be used.^681^ Factors such as the anatomy of the inferior turbinate (if the hypertrophy is more bony and/or more mucosal) and the middle turbinate, the extent of the hypertrophy (if more anterior and/or posterior), and the response to previous interventions, if performed, must be considered when choosing the technique to follow.

Regardless of the technique and equipment, creating more space for air passage and minimizing complications are the desired goals.

It is important to highlight those aggressive procedures, such as extended or even total turbinectomies, do not provide better results. Complications related to nasal turbinate surgery are usually nasal bleeding and temporary crust formation. Techniques that preserve the nasal mucosa are usually preferred by surgeons, due to the lower potential for crust formation (Table 16). More radical procedures or those with large mucosal resections should be avoided, due to cases of empty nose syndrome described in the literature.^682^

**Table 16:** Shows the main techniques used to perform inferior turbinate surgery.

With mucosa preservation	Conventional turbinoplasty
	Turbinoplasty with microdebrider
	Radiofrequency
	*Coblation*
Without mucosa preservation	Conventional turbinectomy (partial or total)
	Turbinectomy with electrocautery
	Laser turbinectomy
	Cryoturbinectomy

Nasal turbinate surgery, when well indicated, improves the quality of life of patients with allergic rhinitis, enables better distribution of topical medications in the nasal cavity and control of the disease.^573^

Regardless of the surgical technique used, patients must be alerted to the need to continue monitoring and clinical or immunotherapy treatment.^683^

## Treatment compliance

Compliance is defined as the extent to which a person's behavior (taking medication, following a diet, and/or making lifestyle changes) matches a healthcare provider's recommendations and involves patient decisions about their treatment. Compliance to AR treatment recommendations is variable and factors related to AR treatment compliance are not well characterized in the literature.^684^

When approaching this topic there is a difficulty in defining it due to (1) Many treatments are carried out as needed; (2) Many patients treat themselves with medicines sold without a prescription, without consulting a doctor; and (3) Researchers more interested in life-threatening conditions may consider non-compliance to AR treatment as a less important topic to study. For these reasons, understanding AR treatment noncompliance and effective interventions to resolve it often lag behind other conditions.^685^, ^686^

Regarding established clinical treatments, Intranasal Corticosteroids (ICS), as they are a prescribed daily treatment, have received greater attention regarding compliance. It is reported that between 28% and 77% of patients with AR report compliance to the recommended ICS treatment regimen all or most of the time. Twenty percent of patients report compliance only for a brief period or only when they experience symptoms.^685^

Rejection of ICS treatment has been attributed to the aftertaste, irritation to the nose and oropharynx, side effects such as nosebleeds and the cost of the medication.^685^, ^686^

Compliance to Allergen Immunotherapy (AIT) has received greater attention than other treatments targeting AR, both because it is prescribed at specific intervals and because benefits depend on long-term compliance.^685^ AIT compliance is variable and depends on the route of administration, Subcutaneous Immunotherapy (SCIT) versus Sublingual (SLIT), frequency/dosage regimen, patient characteristics, in addition to characteristics and adverse events associated with the immunotherapy.^20^

Literature data secondary to various publications on SCIT and SLIT report variable overall compliance rates. Studies examining SCIT compliance have shown mixed results, with compliance rates ranging between 23% and 89%. For SLIT, rates varied between 64% and more than 95%.^687^

A German study evaluated compliance to SCIT and SLIT in 330 patients, including children and adults, enabling a complete three-year cycle of treatment to be considered for each patient investigated. The dropout rate for the entire patient cohort was 34.8%. Overall, patients with SLIT had a slightly higher dropout rate (39.0%) than those with SCIT (32.4%), but with no statistically significant difference between the two groups. The majority of patients who discontinued AIT did so during the first year of treatment.^688^

Another single-center study evaluated 325 children who underwent SCIT or SLIT, according to their parents' wishes. They reported compliance rates as well as adverse events throughout the treatment. The compliance rate was higher in the SCIT group, despite the greater occurrence of adverse effects in this group.^689^

A study followed treatment with AIT for three years in children, adolescents and adults. There was a gradual year-on-year decline in compliance to SCIT and SLIT and compliance at the end of the third year of treatment was 57% and 53% for SCIT and SLIT, respectively.^690^

The reasons why one type of AIT overlaps the other are still controversial: SCIT would provide greater compliance due to regular visits to the doctor's office to perform it, while others assume that SLIT would result in better compliance due to its greater convenience.^20^

To improve compliance there are four points considered to be of particular importance, described as follows: (1) Improving patients' knowledge about the treatment and their disease; (2) Strengthen the partnership between doctors and patients; (3) Provide reliable data in the form of real-life studies and multicenter studies with large numbers of participants; and (4) Standardization of measurement methods.^688^

## Final remarks

This consensus brings together the most up-to-date information on rhinitis, especially allergic rhinitis. Despite the great strides made in recent years, anamnesis remains the fundamental point in the suspected diagnosis of AR and the identification of the phenotype involved, enabling an appropriate treatment plan.

Patients with AR require an initiative-taking and individualized assessment, combining accurate etiological diagnosis with individualized therapy. It is especially important to recognize comorbidities that can negatively impact the patient's AR early on.

One aspect that impacts adequate treatment is low patient compliance, as well as self-medication. A complete approach to one’s disease, the importance of learning about its control, making the best use of medications in relation to the correct technique and time of use will increase patient compliance, guaranteeing better results.

## Conflicts of interest

The authors declare no conflicts of interest.
